# Relating the cortical visual contrast gain response to spectroscopy-measured excitatory and inhibitory metabolites in people who experience migraine

**DOI:** 10.1371/journal.pone.0266130

**Published:** 2022-04-07

**Authors:** Yu Man Chan, Rebecca Glarin, Bradford A. Moffat, Stefan Bode, Allison M. McKendrick

**Affiliations:** 1 Department of Optometry and Vision Sciences, The University of Melbourne, Parkville, Victoria, Australia; 2 Department of Medicine and Radiology, Melbourne Brain Centre Imaging Unit, The University of Melbourne, Parkville, Victoria, Australia; 3 Melbourne School of Psychological Sciences, The University of Melbourne, Parkville, Victoria, Australia; Linköping University, SWEDEN

## Abstract

**Objective:**

This study aimed to determine whether the visual response to flickering checkerboard patterns measured using electroencephalography (EEG) relate to excitatory or inhibitory metabolite levels measured using ultra-high (7Tesla/7T) magnetic resonance spectroscopy (MRS).

**Background:**

Electrophysiological studies have shown altered visual cortical response amplitudes and contrast gain responses to high contrast flickering patterns in people with migraine. These contrast response anomalies have been argued to represent an imbalance between cortical inhibition and excitation, however the specific mechanism has not been elucidated.

**Methods:**

MRS-measured metabolite levels were obtained from the occipital cortex of 18 participants with migraine and 18 non-headache controls. EEG contrast gain response functions were collected on separate days from a subset of 10 participants with migraine and 12 non-headache controls. Case-control outcome measures were statistically compared between groups both before and after checkboard exposure.

**Results:**

No significant difference in GABA and glutamate levels were found between groups nor checkerboard timepoint. Glucose levels were significantly reduced after checkerboard exposure in both participant groups. There was no metabolic signature in visual cortex in response to high-contrast flickering checkboards that distinguished those with migraine and without. There was also no correlation between MRS and EEG measurements in response to the flickering checkerboard.

**Conclusion:**

Our findings suggest that the mechanisms driving contrast-flickering stimulus aversion are not simplistically reflected by gross changes in metabolic activity in the primary visual cortex.

## Introduction

People who experience migraine have heightened sensitivity to light and anecdotally report that temporally modulating stimuli are particularly aversive. For example, when asked to view a flickering screen, migraine sufferers report finding the screen aversive at significantly lower contrasts than control observers [1]. Electrophysiological responses to such high-contrast flickering stimuli, such as a black-white checkerboards, have been studied extensively using electroencephalogram (EEG) by quantifying visual evoked potentials (VEPs) in people who experience migraine [2–5]. Such visual evoked responses in people who experience migraine have previously been reported to be of larger amplitudes than in controls [4]. With prolonged exposure to a flickering checkerboard, VEP amplitudes are expected to decrease in healthy controls (referred to as habituation or adaptation in the literature); however, in those with migraine, VEP amplitude can remain unchanged, or in some cases increase [6]. This observed lack of habituation is proposed to be indicative of an abnormal balance between excitation and inhibition in the visual pathways; however, the specific mechanisms underpinning this observation are not fully elucidated [4, 7, 8].

Exposure to a flickering checkerboard alters the contrast response gain control in the visual system that is proposed to be regulated by the relative balance of cortical excitatory and inhibitory function [3, 5, 6, 9]. Gamma aminobutyric acid (GABA) is the main inhibitory metabolite, while glutamate is the main excitatory metabolite responsible for regulating neuronal inhibition and excitation throughout the brain, respectively. Dysregulation of the expression of these metabolites is therefore a candidate signature for an imbalance between inhibition and excitation. These and other brain metabolites have unique magnetic resonance spectroscopic signatures that can be detected and quantified in an MRI acquisition using magnetic resonance spectroscopy (MRS) techniques [10].

Most prior work investigating cortical metabolites in migraine has used either 1.5T or 3.0T MRI systems, where it is difficult to differentiate the spectral peaks for glutamate and glutamine, therefore they have been reported as a combined concentration (glutamine-glutamate complex, Glx) [11–14]. MRS studies in migraine have previously found increased Glx levels in the visual [13] and anterior paracingulate cortex [12], and during visual stimulation in migraine with aura in the visual cortex [11]. Increased Glx levels in the cerebral spinal fluid and plasma have also been reported interictally (migraine symptom-free time period) [14]. One study using 7T reported glutamate separate from glutamine in the occipital cortex and noted an increased level of glutamate in migraine participants without aura but not in those with aura [15]. Occipital glutamine was not found to be significantly different between migraine and healthy controls [15] thus contributing to the argument that hyperexcitability in the migraine condition is primarily driven by increased glutamate, not glutamine. Elevated cortical Glx has been presumed to be a key indicator of increased excitability of the migraine brain and a contributor to the cortical spreading depression mechanism in migraine aura and to the activation of trigeminovascular pain pathways [16–18]. Additionally, there have been some reports of reduced levels of cortical GABA in migraine measured interictally in the occipital lobe [11, 19]. Reduced inhibition may increase susceptibility to excitatory inputs, or facilitate an apparent increase in excitatory drive; however, these studies do not permit interpretation of whether reduced GABA is a primary feature or secondary to alterations in other metabolites.

In this study, using 7T MRS we first investigated if the levels of GABA and glutamate in the occipital cortex differ between migraine and non-headache sufferers, and then whether further differences were triggered by exposure to a flickering checkerboard. We were specifically interested in relationships between the regulation of the inhibitory and excitatory neurotransmitters due to the intervention of checkerboard exposure. Earlier work using lower field strength MR-spectroscopy has mostly reported excitatory function in terms of the Glx complex, due to difficulty differentiating the spectral peaks [11–13, 20]. Here, we report our results for these two individually as well as in the form of a summed-complex for comparison to previous work. Secondly, we investigated the visual response to flickering contrast using EEG in a subset of the observers. Electrophysiological responses were subsequently correlated with metabolic measures obtained using 7T MRS. We predicted a correlation between EEG-measured contrast gain response and MRS-measured excitatory and inhibitory metabolites (i.e. glutamate and GABA, respectively). We further predicted that persistent visual stimulation would trigger a high metabolic demand in the visual cortex, and therefore alter the concentration of metabolites such as glucose, lactate, aspartate and glutamate. We specifically tested the migraine group interictally because the majority of prior perceptual studies have tested between migraine events when asymptomatic, in order to reveal underlying differences in brain responsivity to visual input.

## Methods

### Participants

Eighteen observers with migraine (11 with aura (26–47 years, mean±sd:33±8 years), 7 without aura (24–40 years, 32±6 years)) and 18 non-headache controls (19–46 years, 31±9 years) participated in the MRS experiment. From the same sample, a subset of 10 observers with migraine (7 with aura (26–42 years, 32±9 years), 3 without aura (30, 26 & 36 years old)) and 12 non-headache controls (19–46 years, 30±9 years) participated in the EEG experiment. The EEG experiment was conducted on a separate day due to the length of each experimental session being prohibitive to combine, and to avoid cumulative interaction effects between responses to the checkboard during the imaging session and the EEG session. For both experiments, testing was required to be conducted interictally (defined as at least 3 days post an acute migraine event, with no subsequent migraine in the 3 days post the test session). Participants were recruited from a laboratory database of individuals interested in research participation and via advertisements posted in The University of Melbourne.

Observers with migraine were diagnosed by a general practitioner or neurologist and had signs and symptoms fulfilling the criteria for migraine with aura or without aura according to The International Classification of Headache Disorders 3-beta [21]. Observers classified under migraine with aura experienced aura (e.g., flashing lights and scintillating scotoma) prior to their headache phase in at least one of their migraine events, and those classified under migraine without aura never experienced an aura. Migraine participants completed the Migraine Disability Assessment (MIDAS) questionnaire that assesses impact severity of migraine on daily lives [22]. Participants also provided details about attack frequency and number of days post-migraine at the time of testing (Table 1). Observers with chronic migraine were excluded.

**Table 1:** Participant demographics including aura (with:MA; without:MO), age in years, gender (M:Male, F:Female), participation in the electrophysiology (EEG) study (Y:Yes, N:No) and Migraine Disability Assessment Severity (MIDAS) questionnaire data collected from the migraine cohort.

ID	Age (years)	Gender	EEG (Y/N)	MIDAS grade	Pain level (0–10)	Attack frequency (per year)	No. of days since last migraine
**MA-01**	29	F	Y	2	8	10	90
**MA-02**	47	M	Y	1	2	6	30
**MA-03**	26	M	Y	4	8	3	100
**MA-04**	36	F	Y	3	8	18	3
**MA-05**	26	F	N	4	7	5	60
**MA-06**	28	F	Y	2	6	20	14
**MA-07**	27	F	N	3	4	30	10
**MA-08**	42	F	Y	2	7	3	180
**MA-09**	42	M	N	3	7	20	18
**MA-10**	28	M	Y	3	5	6	50
**MA-11**	29	F	N	3	8	20	3
**MO-01**	30	M	Y	3	7	25	100
**MO-02**	30	F	Y	3	7	5	3
**MO-03**	26	F	N	2	8	2	40
**MO-04**	40	F	N	2	9	12	30
**MO-05**	24	F	N	1	8	2	110
**MO-06**	35	M	N	2	10	10	3
**MO-07**	36	M	Y	1	6	5	20

Control observers had never experienced a migraine or a migraine aura. They also were not permitted to have experienced more than four spontaneous headaches in the past year, all of which needed to be explicable by known factors such as dehydration or illness such as influenza.

All participants were screened by an optometrist to ensure normal vision for their age and to exclude the presence of any ophthalmic disorder. All had uncorrected or corrected visual acuity of 6/7.5 or better with refractive correction between ±5 dioptres spherical and less than 2 dioptres cylindrical. All participants were also free from medical conditions, other than migraine, and regular medications known to affect cognition and visual function, including migraine prophylactics.

Prior to formal data collection, all participants provided written consent in accordance with project ethics approval by the Human Research Ethics Committee of the University of Melbourne (UMHREC 1443394.4) and protocols defined in the Declaration of Helsinki. Recruitment was conducted between December 2017 –December 2018.

### Checkerboard stimulus

In the MRS experiment, a high contrast flickering checkerboard stimulus (97% Michelson’s contrast, 0.8 degree checks, 53 cd/m^2^ mean luminance, total size of stimulus 31 x 31.7 degree) with a central red fixation square (0.5 degree) was used to stimulate the visual cortex. The checkerboard reversed from light to dark every 60 ms, or one complete cycle from light to dark to light every 120 ms. The stimulus temporal frequency was chosen to elicit a steady-state response at 8.33 Hz. The visual stimulus was presented on an MR-compatible LCD panel (32-inch width, 120Hz frame rate, 1920x1080 pixel resolution) (Cambridge Research Systems, UK) and viewed binocularly from 1.5m.

The same checkerboard stimulus was used during EEG acquisition, but contrast was varied at 6 contrast levels (0, 4, 9, 18, 37, 73, 97% Michelson’s contrast) in separate runs and viewed binocularly from 68 cm. The stimulus was presented on a cathode-ray-tube monitor (mean luminance 115 cd/m^2^, 120 Hz frame rate, 1024x768 pixel resolution, Sony, Trinitron Multiscan G420, Tokyo, Japan). Behavioural responses in the form of catch trials were collected via a Cedrus response button box (RB 540, CA, USA).

### MRS acquisition and data processing

**Fig 1:**
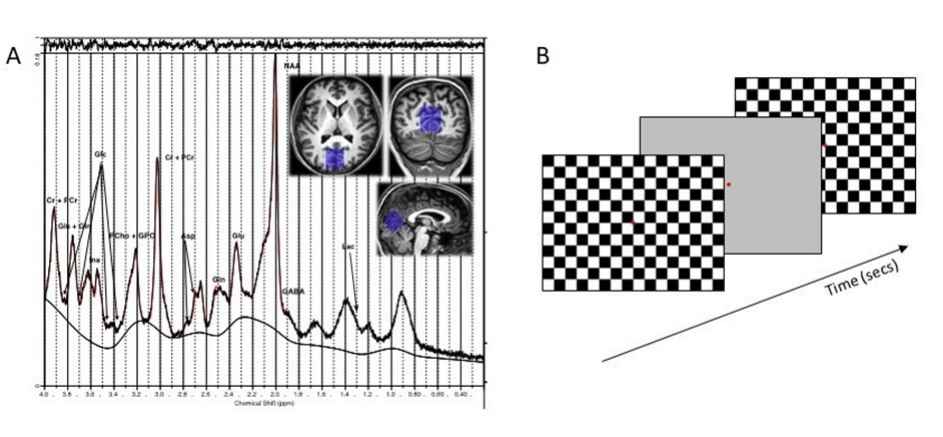
Stimulus example and exemplary spectra from a typical control participant. (A) An example MRS spectrum with approximate peak assignments for known brain metabolites from a 25 year old female participant before stimulation. The FWHM of the peaks were 12 Hz and the SNR was 56, typical of the MRS quality before and after stimulation. The location of the MRS voxel is shown overlaid on the 3D T1 weighted images (inset). (B) Diagram of the flashing checkerboard frames shown during visual cortex stimulation.

MRS data acquisition was performed on a 7T MRI research scanner (Siemens Healthcare, Erlangen, Germany) with a 32-channel head-coil (Nova Medical Inc., Wilmington MA, USA), including the T_1_-weighted whole brain image (MP2RAGE, repetition time [TR] = 4.7s; echo time [TE] = 2.89 ms, 1 mm^3^ isotropic voxels) and single voxel STEAM spectroscopy data (TR = 8.5 s; TE = 6ms; mixing time = 30 ms; voxel size = 30x25x20mm^3^). The visual cortex voxel placement was individually adjusted to be centred midline on either side of the calcarine sulcus and with 6 mm anterior to the dura while avoiding major blood vessels, ventricles and meninges (Fig 1A). The voxel placement varies between individual due to anatomical variance but is placed so that it constitutes mainly V1 plus some associated visual areas. A STEAM sequence [23] with 2.6 ms RF excitation pulses, VAPOR water suppression and outer volume signal suppression was used to acquire the spectra (32 transients, total scan time = 4.5 minutes). The excitation pulse powers for 90 degree tip angles were computed based on the vendor supplied B1 mapping tools. MRS data were eddy-current corrected using the unsuppressed water signal from the same voxel (8 transients).

Metabolite concentrations were estimated by fitting basis sets of alanine (Ala); aspartate (Asp); creatine (Cr); phosphocreatine (PCr); γ-aminobutyricacid (GABA); glucose (Glc); glutamine (Gln); glutamate (Glu); glutathione (GSH); inositol (Ins); lactate (Lac); phosphoethanolamine (PE); scyllo-inositol (sIns); taurine (Tau); N-acetyl-aspartate (NAA) and Acetyl moiety of N-acetylaspartylglutamate (NAAG) to the MRS data using LCModel [24, 25] between 0.2 and 4.0 ppm. The default LCModel macromolecular (14 Voigt lines) and baseline spline (knot separation factor or dkntmn of 0.15) fitting parameters were also used. Metabolites were quantified as institutional units (i.u.) with reference to the unsuppressed water estimate obtained from the same voxel. Spectra were visually inspected by an experienced spectroscopist (BAM) for acceptable quality based on FWHM, SNR, baseline fluctuations and CLRB estimates of key metabolites. Only those estimates of Glu, Gln and GABA with a CLRB of < 30% were reported. Individual spectra are provided in supporting information S1 File and raw data for each individual is provided in S1 Dataset.

The metabolite concentrations (*C*_0_) were then adjusted for tissue composition of the voxel obtained using the FSL version 6.0 FAST [26, 27]. The T_1_-weighted images were segmented into grey matter (*GM*), white matter (*WM*) and cerebrospinal fluid (*CSF*). Metabolite values were then corrected (*C_corr_*) using the following equations [28]:

$$V_{CSF}=\frac{CSF}{\left(GM+WM+CSF\right)}$$ (Eq 1)

$$C_{corr}=C_{0}\times \left(\frac{1}{1-V_{CSF}}\right)$$ (Eq 2)

### EEG acquisition and data preprocessing

The EEG experiment was conducted in a dark room on a 64-bit personal computer (Shuttle, Taiwan) using custom written software using the Psychtoolbox-3 in Matlab (version 2018b). Participants were seated in front of the monitor with their head positioned on a chinrest to maintain a stable binocular viewing distance of 68 cm. Continuous EEG recordings were acquired using a 64-channel BioSemi Active Two system (Amsterdam, Netherlands), using the standard 10/20 system for placing electrodes, at a sampling rate of 512 Hz, and online bandpass filtered from 0.1 to 70 Hz. The vertical and horizontal electrooculogram was recorded from electrodes infraorbital and at the outer canthi of the left eye. The active Ag/AgCl electrodes (actiCAP, Brain Products).

The collected data was first re-referenced against the average of the left and right mastoids and then subjected to a standard EEG preprocessing procedure. The EEG recording was time-locked to the onset of the flickering checkerboard and epoched from 0 ms to 15s post-stimulus onset, capturing the entire stimulation period. A standard 45–55 Hz notch filter was applied to remove 50 Hz electrical noise. Then, a 1–70 Hz bandpass filter was applied. The data set was then subjected to an independent components analysis as implemented in the EEGlab-Toolbox to identify and remove components related to eye movements and eye-blink artefacts [29]. Response amplitudes at the second harmonic (2F, 16.7 Hz) were extracted post-hoc by Discrete Fourier Transformation using the Letswave-6 open-source toolbox (https://www.letswave.org/) in MatLab.

Individual contrast gain functions were then plotted as the extracted 2F response amplitude against the 7 contrast levels. Then, the functions were individually modelled with best fit curves based on the following equation, as per previous work by Nguyen et al. (2016) [4], a modification of the standard hyperbolic (Naka-Rushton) function:

$$R(c)=R_{max}∙\frac{c^{n}}{c^{sn}+{c_{50}}^{sn}}+R_{0}$$ (Eq 3)

where R_0_ is the response at 0% contrast, *n* is the excitatory exponent, and *s* is the suppressive exponent. All parameters in the equation were floated and that all parameters were positive, and that c_50_ (semisaturation constant) must not exceed the maximum Michelson contrast (100%). Best fit functions were optimised by minimising the sum of squares.

### Correlations

We correlated the EEG and MRS measurements in the subgroup of observers who participated in both parts of the study to investigate the general relationship between EEG-measured contrast response amplitude and MRS-measured metabolite metabolism (Control n = 12, Migraine n = 10). This is limited to an exploratory analysis since the two types of measures were acquired on separate days for each individual. Given there were no observed group differences in either the MRS and EEG measures, correlations were conducted on the pooled data across controls and people with migraine, which also increased the statistical power for this approach (total n = 22). As a single summary measure of EEG response to high contrast, we first computed the response amplitude to 97% contrast checkerboard (R97) based on individual best fit curves on the raw data. The difference between R97 and R0 (response amplitude to 0% contrast checkerboard, i.e. baseline) was computed for each individual and then compared to the difference between post- and pre-checkerboard metabolite levels.

### Procedure

Participants were invited to participate in both the MRS and EEG test sessions which ran for 2 hours and 3 hours respectively, on separate days. Some participants only participated in the MRS study, mostly due to the 3 hour commitment required for the EEG session. It was not feasible to run both test sessions on the same day due to pragmatics of the extended test duration, requirement of participants to refrain from caffeine for the day, and because of possible cumulative aversive effects of the high contrast flickering stimuli in the migraine group. All participants who attended both the MRS and EEG sessions were tested on separate days between 1 week to 2 months apart, with both sessions being scheduled for the interictal phase for those with migraine. Participants attended either test session first, whichever was available.

In the MRS session, a baseline measure of metabolites was acquired while participants passively viewed a constant grey background at mean luminance (53 cd/m^2^) with a red fixation square (0.5-degree visual angle) at the centre of the screen. This was followed by the checkerboard phase in which the flickering checkerboard was presented for 15 sec which then reverted to a 5 sec grey background before commencing on another 15 sec checkerboard stimulation (see schematic diagram in Fig 1B). Five second grey background intervals between each 15 sec checkerboard recording was included to minimise adaptation effects. Participants were instructed to maintain fixation on the red fixation square at the centre of the screen at all times. The checkerboard phase lasted for a total of 10 minutes. To maintain alertness, participants had to indicate that they perceived the onset of the background interval via a button press (catch trials). A post-checkerboard measure of metabolites was then acquired immediately after this checkerboard phase while passively viewing a constant grey background and red fixation square identical to that at baseline.

In the EEG session, continuous EEG recordings were measured in response to flickering checkerboards at Michelson’s contrast levels of 4, 9, 18, 37, 73 and 97% in separate runs. Participants completed one run at each of the six contrast levels. Test order was always fixed from the lowest to the highest contrast level to minimise any possible effects of contrast adaptation. Each run started with a 1 min baseline measurement to a constant grey background at mean luminance (53 cd/m^2^) with a red fixation square (0.5 degree visual angle) at the centre of the screen. This was immediately followed by the onset of the flickering checkerboard at the predefined fixed contrast level at the same timing profile as in the MRS recording. Each run terminated at the end of 12 x 15 sec checkerboard to give a total of 180 sec EEG recording to the flickering checkerboard contrast response. The total test time to complete all contrast levels was approximately 40 minutes allowing for brief breaks between runs. Behavioural responses to catch trials were collected in the same format as described above in the MRS procedure.

### Statistical analysis

Statistical analyses were performed using IBM SPSS Statistics 26 (New York, USA). The metabolites of interest were first assessed for normality using the Shapiro-Wilk test. Then the data for each metabolite was analysed in separate RM-ANOVAs, with checkerboard timepoint as the within-subject variable (*pre-checkerboard or post-checkerboard*) and group as the between-subject variable (*control or migraine*). Raw EEG response amplitudes and normalised amplitudes (normalised to individual response amplitude at 97% contrast) were assessed in 2 separate RM-ANOVAs, each with contrast as the within-subject variable and group as the between-subject variable. Pearson’s correlations were used to determine the relationships, if any, between the MRS-measures and EEG-measures. Bayes factors are reported calculated using the BayesFactor package in R: https://cran.r-project.org/package=BayesFactor.

## Results

### Voxel segmentation outcomes

As described in the methods, individual metabolite levels were corrected for voxel proportions. The mean proportion of CSF, grey and white matter in the voxel were 0.113, 0.619, 0.268 in the control group and 0.109, 0.616, 0.276 in the migraine group. Individual voxel proportions are provided in the supporting information S1 Dataset.

### Did the inhibitory and excitatory MRS measures change with checkerboard exposure?

**Fig 2:**
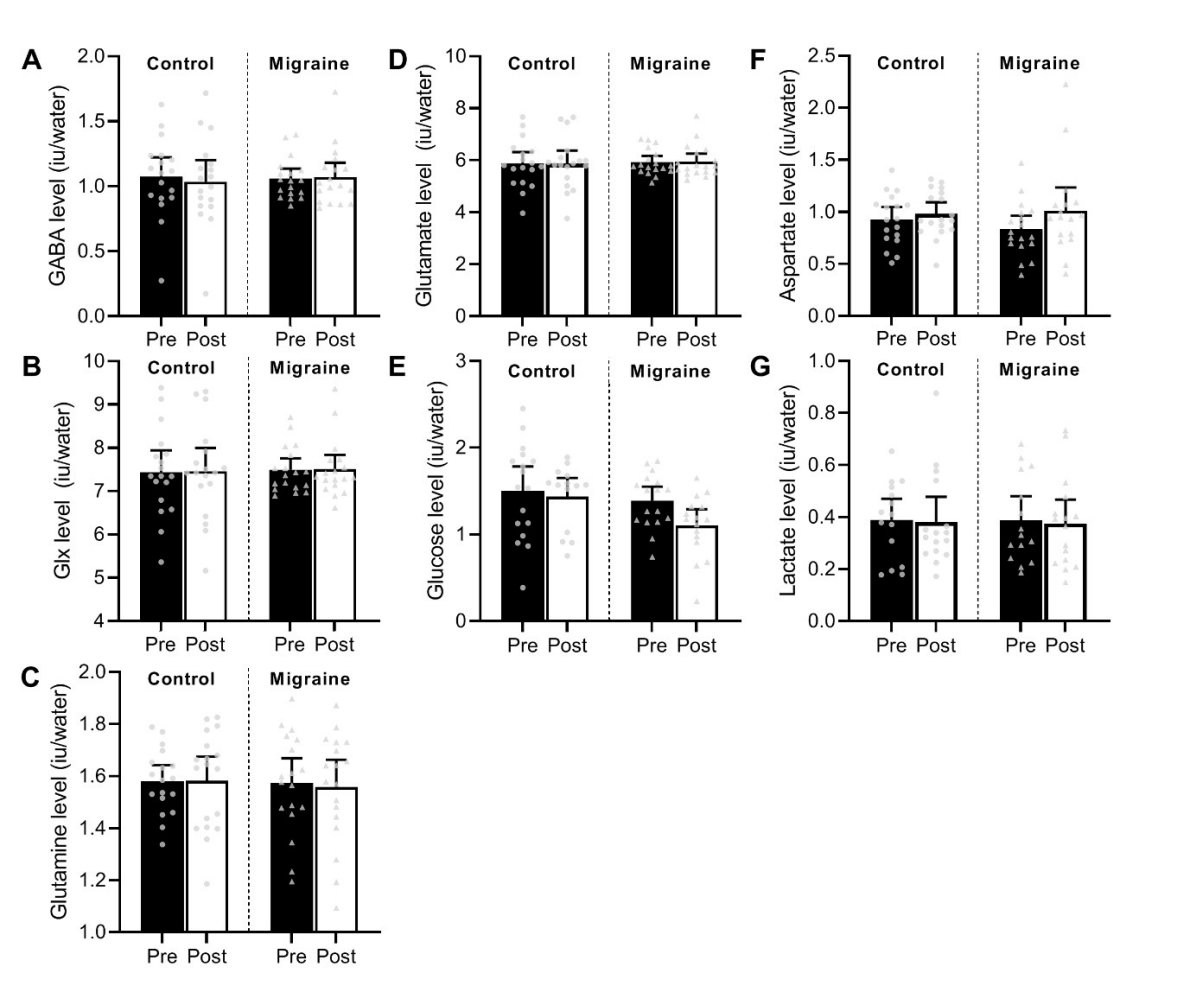
Group averaged metabolite levels referenced to water. Group averaged metabolite levels (referenced to water) for (A) GABA, (B) Glx, (C) Glutamine (Gln), (D) Glutamate (Glu), (E) Glucose (Glc), (F) Aspartate (Asp) and (G) Lactate (Lac) pre- and post-checkerboard. Group averaged data and individual data are shown here (pre-checkerboard: dark bars, controls: circles; post-checkerboard: light bars, migraine: triangles) with error bars as 95% confidence intervals of the mean.

Firstly, we assessed if the main excitatory and inhibitory metabolites differed between groups (control vs migraine), and if they were altered by the exposure to the flickering checkerboard (timepoint: pre and post exposure to checkerboard). GABA levels did not differ between groups (no main effect of group: F(1,34) = 0.02, p = 0.90) and there was no significant interaction between group and timepoint: (F(1,34) = 0.72, p = 0.40). There was also no main effect of timepoint on GABA levels (F(1,34) = 0.21, p = 0.65) (Fig 2A). Similarly, Glx levels did not differ between groups (no main effect of group: F(1,34) = 0.04, p = 0.85) and there was no significant interaction between group and timepoint: (F(1,34) = 0.001, p = 0.97). There was also no main effect of timepoint on Glx levels: (F(1,34) = 0.06, p = 0.81) (Fig 2B).

Next, we assessed the excitatory complex as separate metabolites: glutamate and glutamine. Glutamine and glutamate levels were not different between groups (no main effect of group: F(1,34) = 0.08, p = 0.78; F(1,34) = 0.07, p = 0.80) and the interaction between group and timepoint (pre- and post-checkerboard) was also not significant (F(1,34) = 0.16, p = 0.69; F(1,34) = 0.01, p = 0.92). These two metabolites were also not significantly changed comparing post- and pre-exposure to the flickering checkerboard in both groups (no main effect of timepoint: F(1,34) = 0.10, p = 0.75; F(1,34) = 0.16, p = 0.69) (Fig 2C & 2D).

### Did metabolites related to neural energy demand change with exposure to the flickering checkerboard?

As the next step, we analysed the MRS data to determine if exposure to the flickering checkerboard altered indicators of neural energy consumption. Glucose levels did not differ between groups (no main effect of group: F(1,34) = 0.60, p = 0.44), and there was no significant interaction between group and timepoint: (F(1,34) = 0.03, p = 0.87). However, comparing pre- to post-checkerboard, glucose levels were significantly reduced (main effect of timepoint: F(1,34) = 8.78, p = 0.01) (Fig 2E).

The levels of the remaining key metabolites relating to neural energy demand, aspartate, lactate and glutamate, did not differ between groups (no main effect of group: F(1,34) = 0.41, p = 0.53; F(1,34) = 0.002, p = 0.97; F(1,34) = 0.07, p = 0.80) and timepoint (F(1,34) = 2.88, p = 0.10; F(1,34) = 0.08, p = 0.79; F(1,34) = 0.16, p = 0.69). There was also no significant interaction between group and timepoint for any of these three metabolites (Asp: F(1,34) = 0.38, p = 0.54; Lac: F(1,34) = 0.10, p = 0.75, Glu: F(1,34) = 0.01, p = 0.92).

### Did EEG-measured contrast gain response functions differ between controls and migraine observers?

**Fig 3:**
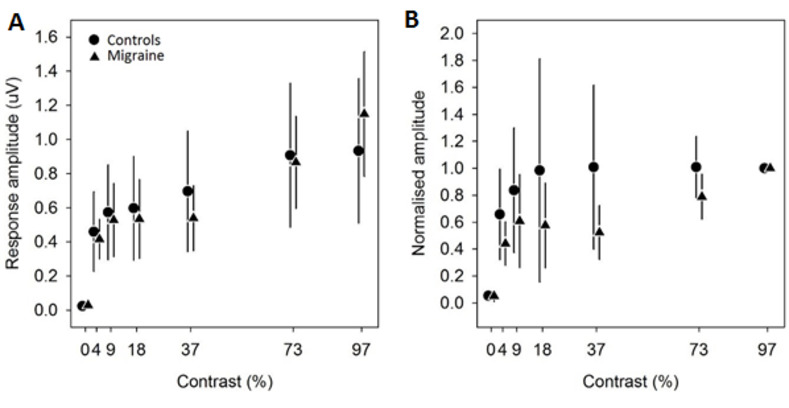
Contrast response gain functions. Contrast response gain functions shown in terms of the raw EEG response amplitude (A) and normalised to individual responses at 97% checkerboard (B). Symbols represent group averaged data (controls: circles, migraine: triangles) with error bars as 95% confidence intervals of the mean.

Next, we evaluated if the EEG-measured contrast gain response functions differed between controls and migraine observers. There was a main effect of contrast (F(6,120) = 15.31, p < 0.0001) but no main effect of group (F(1,20) = 0.95, p = 0.47) nor a significant interaction between contrast and group (F(6,120) = 0.96, p = 0.45) (Fig 3A).

To account for possible effects of supersaturation in some observers (5), response amplitudes were normalised to individual amplitudes measured at 97% contrast (Fig 3B). Normalised amplitudes were significantly different between contrasts (main effect of contrast: F(6,120) = 5.71, p < 0.0001) but not different between groups (F(1,20) = 0.41, p = 0.53). There was no significant interaction between contrast and group (F(6,120) = 0.61, p = 0.73).

### Did the individual change in pre-post checkerboard levels of MRS-measured metabolites correlate with EEG contrast gain estimates?

Upon correcting for multiple comparisons (Bonferroni correction; p<0.008), no significant correlation was present between the change in EEG response amplitude (R97-R0) with GABA, glutamine, glutamate, glucose, aspartate nor lactate levels (see Table 2 for detailed statistics). For Lactate, there was a weak-to-moderate support for the alternate hypothesis (a correlation between the change in lactate and the change in EEG amplitude) over the null hypothesis.

**Table 2:** Pearson’s correlation and Bayes factors for the correlations between the change in MRS-measured metabolites with the change in EEG response amplitude (R97-R0).

	GABA	Glutamine	Glutamate	Glucose	Aspartate	Lactate
**Pearson’s r**	0.08	0.01	0.02	0.02	-0.02	0.45
***p*-value**	0.74	0.98	0.95	0.94	0.93	0.04
**Bayes Factor (BF_10_)**	0.46	0.46	0.47	0.46	0.49	3.01

## Discussion

Using 7T imaging, we did not find any group differences between those with migraine and those without in occipital cortex GABA nor Glx. This finding is in contrast with differences previously reported using the lower field strengths 1.5T and 3T [11–14, 16–19, 30]. The distinct glutamate and glutamine peaks that are measurable at 7T were also not different between groups. We had a relatively small sample size (18 non headache controls and 18 with migraine); however, we note that previous MRS studies examining migraine cohorts also did not include large samples (González de la Aleja et al., 2013 [12]: 27M, 19C; Bridge et al., 2015: 11M, 11C). We powered our study based on assumptions for the occipital GABA levels reported by Bridge et al. (2015) [11] (power of 80% and alpha rate of 0.05). We also note that previous studies have normalised metabolite estimates to creatine instead of water. However, when we re-analysed our MRS data referenced to creatine instead of water, we still obtained no differences, as in the reported findings (Supporting information S1 Appendix). Given that 7T imaging provides better sensitivity and precision than lower field strength machines in quantifying weakly represented metabolites such as glutamate in small volumes of interest [31], the negative findings here suggest that if there is an effect of visual stimulation or an effect of migraine condition, it is most likely to be weak. Indeed, calculated Bayes Factors in support of the null hypothesis (BF_01_) for our data for the GABA and Glx between group baseline comparisons are 5.86 and 5.80, respectively (i.e. the observed data is approximately 5.8 times more likely under the null hypothesis (no difference) than the alternative hypothesis [31]).

Migraine is a highly heterogeneous condition, hence there is the potential for study results to differ depending on the migraine sample included. Perceptual and neurophysiological measures in migraine patients can vary due to age, attack frequency, age of first migraine onset, pain level, days since last attack and migraine aura, to mention a few. Although most studies have ensured that migraineurs were tested interictally (when they were asymptomatic), data from our lab have shown that visual function such as contrast processing varies with the number of days pre-migraine within individuals [32]. Our experiment was specifically designed to explore interictal differences between those with migraine and those without, rather than the active migraine phase, because people with migraine report aversion of high-contrast patterns throughout the migraine cycle [32]. We deliberately did not tightly control the duration of time post-migraine in this study to be comparable to previous studies, including reporting of aversion to flickering stimuli [32–38]. We explored whether there was any correlation between the spectroscopy measures relative to the number of days post-migraine but found no significant correlation in our dataset (all p>0.05, data not shown). However, caution in interpretation is required because our study was not a priori designed to investigate this question (thus recruitment was not intentional to include migraineurs evenly spread over a range of days post-migraine). There may also potentially be a sampling bias due to the fact that participants were aware that they were signing up for an experiment that required them to view flickering checkerboards. Consequently, people who find these stimuli highly aversive may have avoided participating.

We did not find a correlation between our EEG-measured visual response to the flickering checkerboard and spectroscopy-measured GABA and glutamate. It should be noted that the spectroscopy and EEG measures were obtained on separate sessions on separate days, but both were within the interictal period (more than 4 days post-migraine). Interpretations of negative findings require care; however, it seems fair to conclude from our study that if there was a significant relationship between EEG responses and MRS metabolites that we could not detect, it is likely to be weak. For future work, a more directed design whereby continuous EEG and MRS are collected within the scanner before, during and after exposure to the flickering checkerboard in the same test session in the same group of individuals will provide more directly comparable measurements between the two techniques.

Furthermore, it should be noted that MRS has limitations for inferring mechanistic relationships between measured metabolites and both perceptual and EEG responses. Importantly, MRS does not quantify synaptic neurotransmitter activity, which may be more likely than overall metabolite concentration to be directly related to perception and EEG responses. Previous studies that have investigated the link between MRS-measured GABA (resting state) and evoked potentials have focussed on gamma band oscillations. Studies conducted in the visual [39] and auditory [40] domains consistently reported a null relationship between the MRS and electrophysiological measures. However, the motivation for the current study was based on studies that have reported a significant correlation between MRS-measured metabolites and perceptual analogues of visual inhibition such as binocular rivalry [41–43], surround contrast suppression [44, 45], motion suppression [42] and orientation discrimination [46].

In this study we have used STEAM to acquire the MR spectra. This sequence has advantages because very short echo times can be achieved because during the time between the 2^nd^ and 3^rd^ RF pulses the magnetization stays in the longitudinal direction is subject only to T1 relaxation. This means that with a long TR, as in this study, relaxation processes do not need to be corrected for. A disadvantage is that there is generally a loss of 50% of SNR. Also important for MRS of GABA is the spectral resolution whereby the GABA resonances are very close to other metabolite resonances. We [47] and others [48] have shown though that at 7T lcmodel on STEAM acquired MRS, GABA can be successfully resolved. Another approach for quantifying GABA is to use subtraction of spectral edited sequences [48, 49] (where some peaks are inverted). While this makes GABA resonances more obvious to the human eye on the spectra, it does not appear to improve the quantification via model fitting [48] over STEAM spectra.

A more recent study in 2017 by Zielman et al. [15] reported an increase in glutamate levels in participants with migraine without aura compared to healthy controls but not in those with aura. They used a different acquisition approach–semi-LASER while the current study used the STEAM sequence. They also had their participant keep their eyes closed throughout the scanning, whereas we presented our participants with the flickering checkerboard. This could mean that glutamate levels at resting state may be elevated in people with migraine without aura as compared to controls (as shown by Zielman et al. 2017), but in the presence of visual stimulation, glutamate levels reach a similar concentration between groups (as shown in the current study). In addition, although we tested people with and without aura, the current study was not aimed to explore metabolite differences between the two group, and the sizes of our sub-groups would not be sufficiently large to identify differences between aura groups. To make a conclusive statement on this, a future study will need to be specifically designed to measure glutamate levels in non-headache controls, migraine sufferers with aura, and without aura, under two conditions: resting state and with visual stimulation.

In terms of cellular metabolism and energy demand resulting from the persistent visual stimulation, metabolites of interest were lactate, glucose, glutamate and aspartate. We found a significant decrease of approximately 25% in spectroscopy measured glucose levels post-exposure to the flickering checkboard in both participant groups. This is in line with earlier work by Mangia et al. [50] who reported a trend for a decrease, but only a 12% change, in occipital glucose concentration in healthy human observers after 10 minute exposure to a flickering radial checkerboard [51]. However, the results from the current study indicate that exposure to the flickering checkerboard did not alter lactate, aspartate nor glutamate levels significantly. Based on the cellular glycolysis cycle, it is expected that decreasing glucose would contrast with increasing lactate, and a reduction in aspartate with an increase in glutamate [50, 52–54]. An increase in lactate level is often interpreted as an increase in oxidative metabolism to generate energy to fuel activated neurons. The aspartate and glutamate change are indicative of an active transamination reaction between the two compounds that similarly implies an increase in oxidative metabolism to fuel energy to activated neurons [55]. Despite not finding a significant change in lactate, aspartate and glutamate post- than pre-checkerboard, the increased consumption of glucose (reduced glucose post- compared to pre-checkerboard) supports the notion of heightened cellular metabolism after viewing the checkerboard stimuli.

## Conclusion

Using ultra-high magnetic field MRS, we were unable to demonstrate a metabolic signature in visual cortex in response to high-contrast flickering checkboards that distinguishes those with and without migraine. Our data shows increased consumption of glucose post-checkerboard indicating the presence of heightened cellular metabolism after watching these aversive stimuli, but this did not differentiate those with migraine, nor did the electrophysiologically measured contrast gain response. While we report no significant difference in interictal measures of excitatory and inhibitory markers post-exposure to visual stimulation, further work may be warranted that specifically target observers with migraine during their pre-ictal period and those who specifically find flickering stimuli aversive.

## Supporting information

S1 File Individual spectra (DOI: 10.26188/19083596). (PDF)

S1 Dataset Raw data including voxel segmentation for individual participants. (XLSX)

S1 Appendix Results based on metabolite levels referenced to creatine. (DOCX)
