# Supplementary material for: Relating the cortical visual contrast gain response to spectroscopy-measured excitatory and inhibitory metabolites in people who experience migraine
Source: PLoS One. 2022 Apr 7;17(4):e0266130. doi: 10.1371/journal.pone.0266130 (PMC8989360; doi:10.1371/journal.pone.0266130)
Supplement: S1 File — (PDF) [file pone.0266130.s001.pdf]

MIGRAINE WITH AURA-29yo-Female-Pre-checkerboard

LCModel (Version 6.3-0B) Copyright: S.W. Provencher. *Ref.: Magn. Reson. Med. 30:672-679 (1993).* *30-July-2018 15:16*

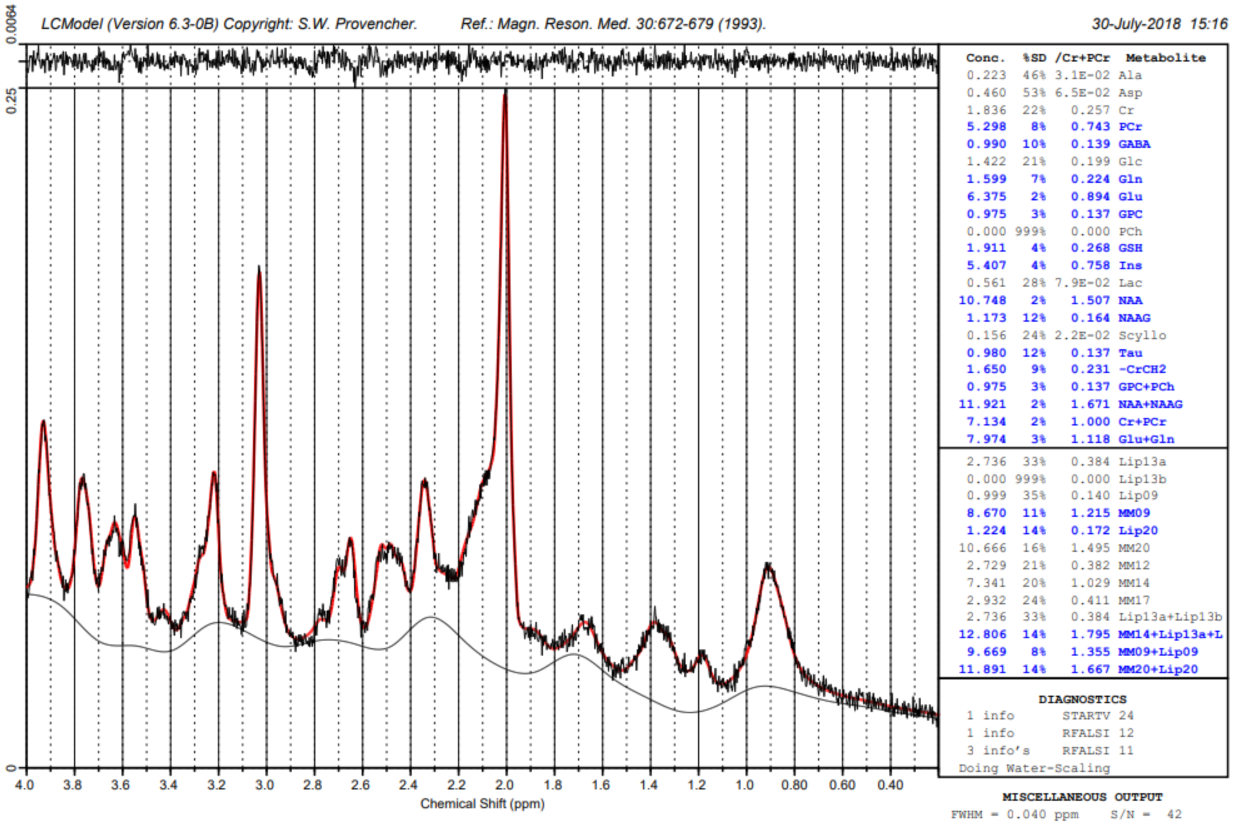

Conc.	%SD	/Cr+PCr	Metabolite
0.223	46%	3.1E-02	Ala
0.460	53%	6.5E-02	Asp
1.836	22%	0.257	Cr
**5.298**	**8%**	**0.743**	**PCr**
**0.990**	**10%**	**0.139**	**GABA**
1.422	21%	0.199	Glc
**1.599**	**7%**	**0.224**	**Gln**
**6.375**	**2%**	**0.894**	**Glu**
**0.975**	**3%**	**0.137**	**GPC**
0.000	999%	0.000	PCh
**1.911**	**4%**	**0.268**	**GSH**
**5.407**	**4%**	**0.758**	**Ins**
0.561	28%	7.9E-02	Lac
**10.748**	**2%**	**1.507**	**NAA**
**1.173**	**12%**	**0.164**	**NAAG**
0.156	24%	2.2E-02	Scyllo
**0.980**	**12%**	**0.137**	**Tau**
**1.650**	**9%**	**0.231**	**-CrCH2**
**0.975**	**3%**	**0.137**	**GPC+PCh**
**11.921**	**2%**	**1.671**	**NAA+NAAG**
**7.134**	**2%**	**1.000**	**Cr+PCr**
**7.974**	**3%**	**1.118**	**Glu+Gln**
2.736	33%	0.384	Lip13a
0.000	999%	0.000	Lip13b
0.999	35%	0.140	Lip09
**8.670**	**11%**	**1.215**	**MM09**
**1.224**	**14%**	**0.172**	**Lip20**
10.666	16%	1.495	MM20
2.729	21%	0.382	MM12
7.341	20%	1.029	MM14
2.932	24%	0.411	MM17
2.736	33%	0.384	Lip13a+Lip13b
**12.806**	**14%**	**1.795**	**MM14+Lip13a+L**
**9.669**	**8%**	**1.355**	**MM09+Lip09**
**11.891**	**14%**	**1.667**	**MM20+Lip20**

DIAGNOSTICS

1 info STARTV 24
1 info RFALSI 12
3 info's RFALSI 11
Doing Water-Scaling

MISCELLANEOUS OUTPUT

FWHM = 0.040 ppm S/N = 42
Data shift = 0.040 ppm

MIGRAINE WITH AURA-29yo-Female-Post-checkerboard

LCModel (Version 6.3-0B) Copyright: S.W. Provencher. *Ref.: Magn. Reson. Med. 30:672-679 (1993).* *30-July-2018 14:49*

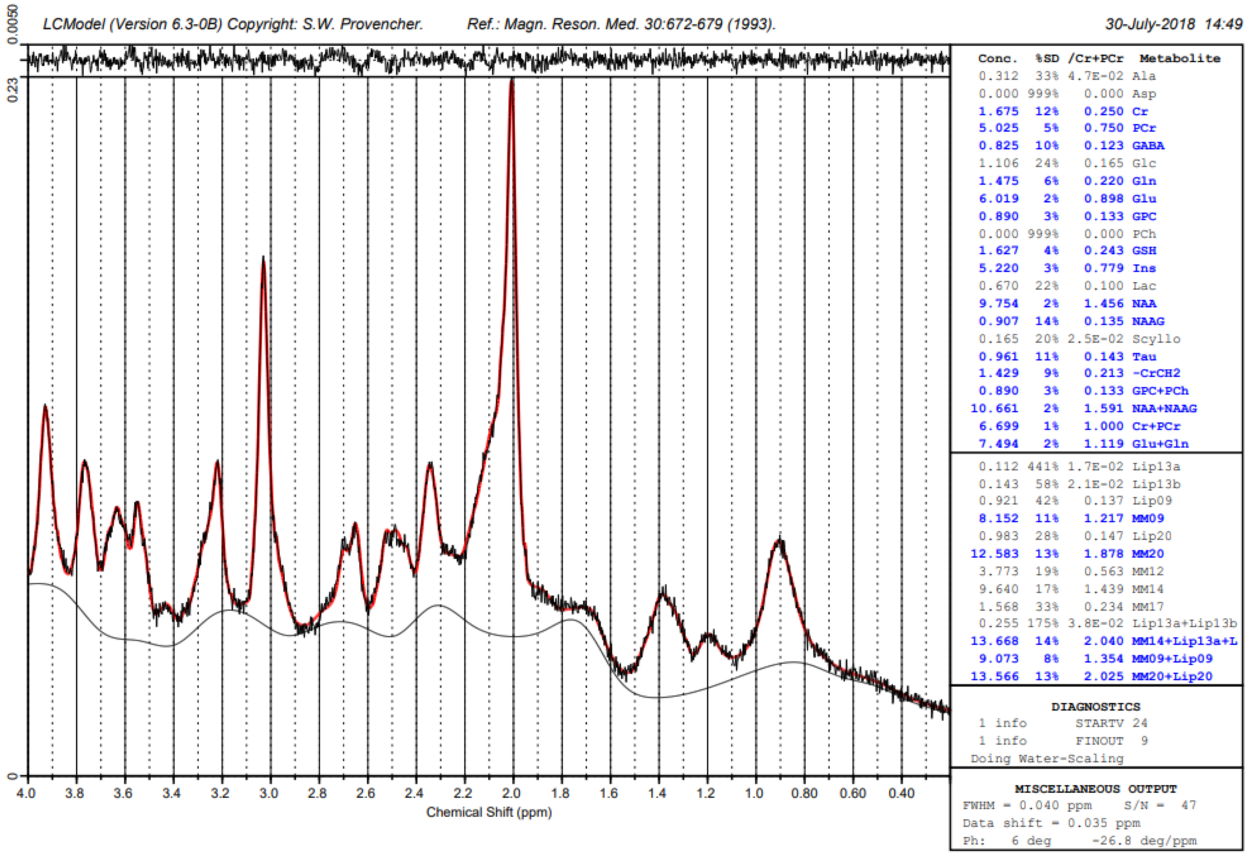

Conc.	%SD	/Cr+PCr	Metabolite
0.312	33%	4.7E-02	Ala
0.000	999%	0.000	Asp
1.675	12%	0.250	Cr
5.025	5%	0.750	PCr
0.825	10%	0.123	GABA
1.106	24%	0.165	Glc
1.475	6%	0.220	Gln
6.019	2%	0.898	Glu
0.890	3%	0.133	GPC
0.000	999%	0.000	PCh
1.627	4%	0.243	GSH
5.220	3%	0.779	Ins
0.670	22%	0.100	Lac
9.754	2%	1.456	NAA
0.907	14%	0.135	NAAG
0.165	20%	2.5E-02	Scyllo
0.961	11%	0.143	Tau
1.429	9%	0.213	-CrCH2
0.890	3%	0.133	GPC+PCh
10.661	2%	1.591	NAA+NAAG
6.699	1%	1.000	Cr+PCr
7.494	2%	1.119	Glu+Gln
0.112	441%	1.7E-02	Lip13a
0.143	58%	2.1E-02	Lip13b
0.921	42%	0.137	Lip09
8.152	11%	1.217	MM09
0.983	28%	0.147	Lip20
12.583	13%	1.878	MM20
3.773	19%	0.563	MM12
9.640	17%	1.439	MM14
1.568	33%	0.234	MM17
0.255	175%	3.8E-02	Lip13a+Lip13b
13.668	14%	2.040	MM14+Lip13a+L
9.073	8%	1.354	MM09+Lip09
13.566	13%	2.025	MM20+Lip20

DIAGNOSTICS

1 info STARTV 24
1 info FINOUT 9
Doing Water-Scaling

MISCELLANEOUS OUTPUT

FWHM = 0.040 ppm S/N = 47
Data shift = 0.035 ppm
Ph: 6 deg -26.8 deg/ppm

C-21yo-Female-Pre-checkerboard

LCModel (Version 6.3-0B) Copyright: S.W. Provencher. Ref.: Magn. Reson. Med. 30:672-679 (1993). 31-July-2018 13:50

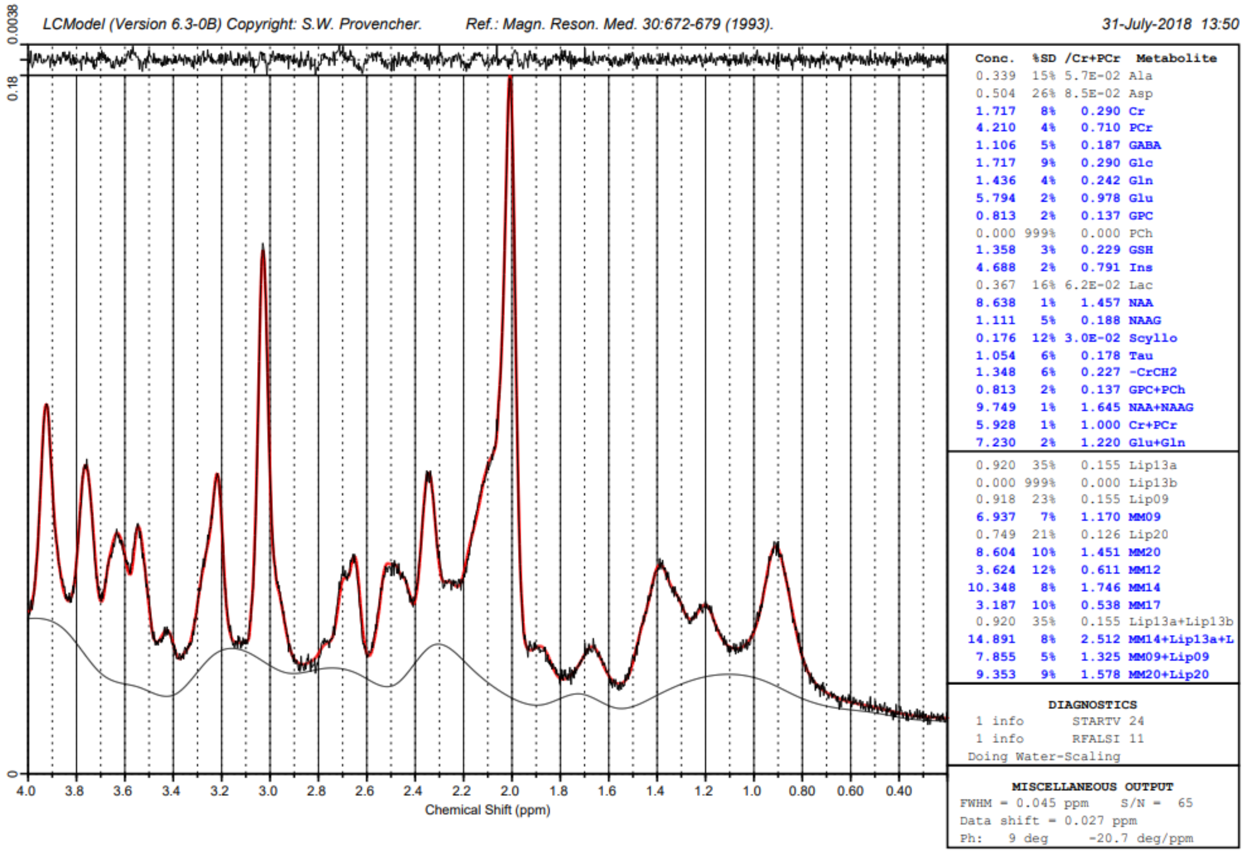

Conc.	%SD	/Cr+PCr	Metabolite
0.339	15%	5.7E-02	Ala
0.504	26%	8.5E-02	Asp
1.717	8%	0.290	Cr
4.210	4%	0.710	PCr
1.106	5%	0.187	GABA
1.717	9%	0.290	Glc
1.436	4%	0.242	Gln
5.794	2%	0.978	Glu
0.813	2%	0.137	GPC
0.000	999%	0.000	PCh
1.358	3%	0.229	GSH
4.688	2%	0.791	Ins
0.367	16%	6.2E-02	Lac
8.638	1%	1.457	NAA
1.111	5%	0.188	NAAG
0.176	12%	3.0E-02	Scyllo
1.054	6%	0.178	Tau
1.348	6%	0.227	-CrCH2
0.813	2%	0.137	GPC+PCh
9.749	1%	1.645	NAA+NAAG
5.928	1%	1.000	Cr+PCr
7.230	2%	1.220	Glu+Gln
0.920	35%	0.155	Lip13a
0.000	999%	0.000	Lip13b
0.918	23%	0.155	Lip09
6.937	7%	1.170	MM09
0.749	21%	0.126	Lip20
8.604	10%	1.451	MM20
3.624	12%	0.611	MM12
10.348	8%	1.746	MM14
3.187	10%	0.538	MM17
0.920	35%	0.155	Lip13a+Lip13b
14.891	8%	2.512	MM14+Lip13a+L
7.855	5%	1.325	MM09+Lip09
9.353	9%	1.578	MM20+Lip20

DIAGNOSTICS

1 info STARTV 24
1 info RFALSI 11
Doing Water-Scaling

MISCELLANEOUS OUTPUT

FWHM = 0.045 ppm S/N = 65
Data shift = 0.027 ppm
Ph: 9 deg -20.7 deg/ppm

C-21yo-Female-Post-checkerboard

LCModel (Version 6.3-0B) Copyright: S.W. Provencher. Ref.: Magn. Reson. Med. 30:672-679 (1993). 31-July-2018 13:50

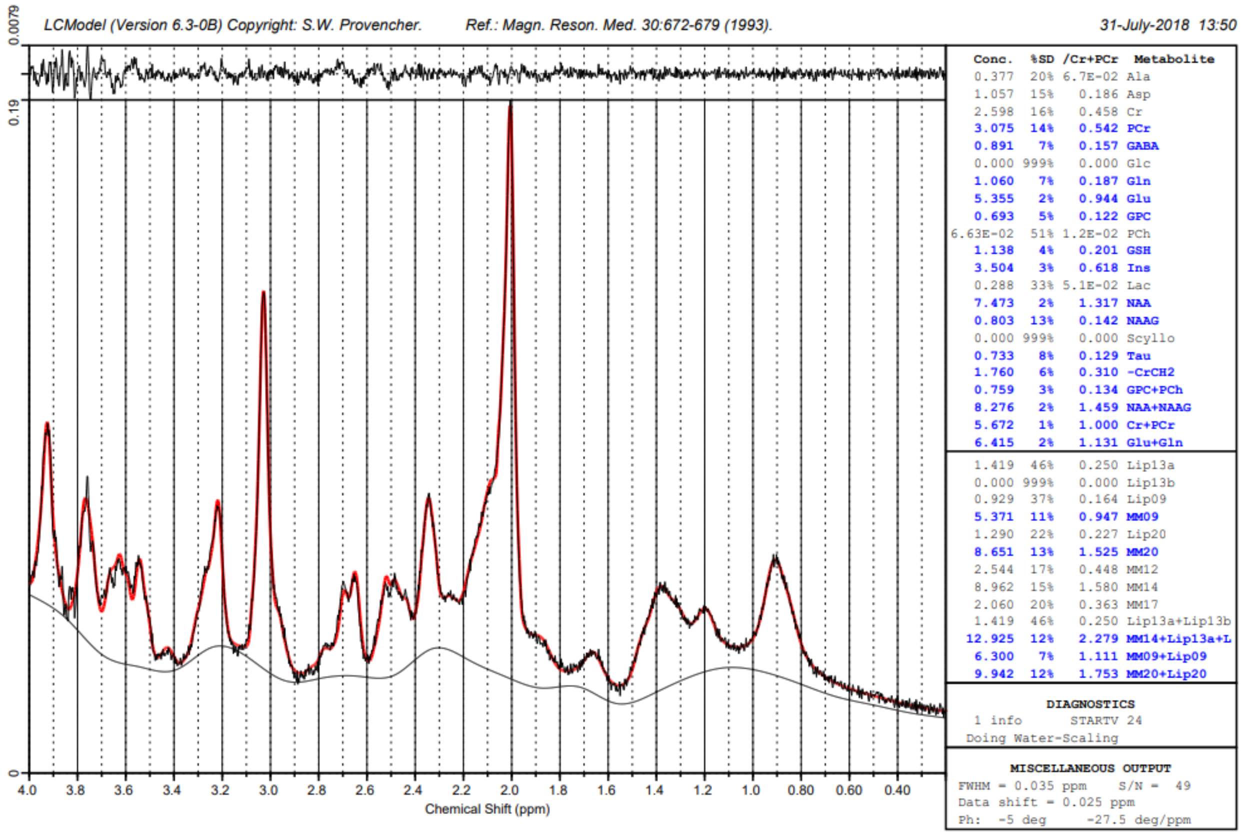

Conc.	%SD	/Cr+PCr	Metabolite
0.377	20%	6.7E-02	Ala
1.057	15%	0.186	Asp
2.598	16%	0.458	Cr
3.075	14%	0.542	PCr
0.891	7%	0.157	GABA
0.000	999%	0.000	Glc
1.060	7%	0.187	Gln
5.355	2%	0.944	Glu
0.693	5%	0.122	GPC
6.63E-02	51%	1.2E-02	PCh
1.138	4%	0.201	GSH
3.504	3%	0.618	Ins
0.288	33%	5.1E-02	Lac
7.473	2%	1.317	NAA
0.803	13%	0.142	NAAG
0.000	999%	0.000	Scyllo
0.733	8%	0.129	Tau
1.760	6%	0.310	-CrCH2
0.759	3%	0.134	GPC+PCh
8.276	2%	1.459	NAA+NAAG
5.672	1%	1.000	Cr+PCr
6.415	2%	1.131	Glu+Gln
1.419	46%	0.250	Lip13a
0.000	999%	0.000	Lip13b
0.929	37%	0.164	Lip09
5.371	11%	0.947	MM09
1.290	22%	0.227	Lip20
8.651	13%	1.525	MM20
2.544	17%	0.448	MM12
8.962	15%	1.580	MM14
2.060	20%	0.363	MM17
1.419	46%	0.250	Lip13a+Lip13b
12.925	12%	2.279	MM14+Lip13a+L
6.300	7%	1.111	MM09+Lip09
9.942	12%	1.753	MM20+Lip20

DIAGNOSTICS

1 info STARTV 24

Doing Water-Scaling

MISCELLANEOUS OUTPUT

FWHM = 0.035 ppm S/N = 49

Data shift = 0.025 ppm

Ph: -5 deg -27.5 deg/ppm

C-18yo-Female-Pre-checkerboard

LCModel (Version 6.3-0B) Copyright: S.W. Provencher. Ref.: Magn. Reson. Med. 30:672-679 (1993). 31-July-2018 13:55

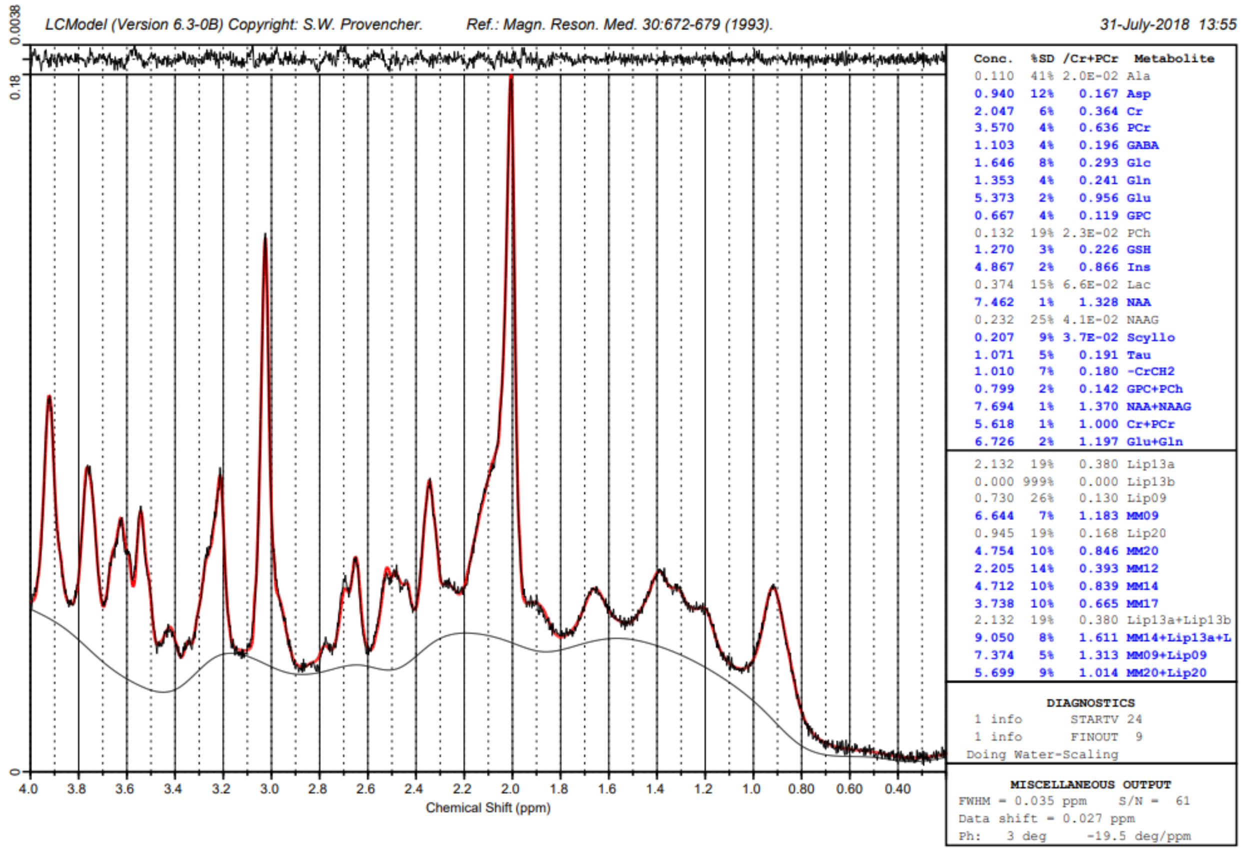

Conc.	%SD	/Cr+PCr	Metabolite
0.110	41%	2.0E-02	Ala
0.940	12%	0.167	Asp
2.047	6%	0.364	Cr
3.570	4%	0.636	PCr
1.103	4%	0.196	GABA
1.646	8%	0.293	Glc
1.353	4%	0.241	Gln
5.373	2%	0.956	Glu
0.667	4%	0.119	GPC
0.132	19%	2.3E-02	PCh
1.270	3%	0.226	GSH
4.867	2%	0.866	Ins
0.374	15%	6.6E-02	Lac
7.462	1%	1.328	NAA
0.232	25%	4.1E-02	NAAG
0.207	9%	3.7E-02	Scyllo
1.071	5%	0.191	Tau
1.010	7%	0.180	-CrCH2
0.799	2%	0.142	GPC+PCh
7.694	1%	1.370	NAA+NAAG
5.618	1%	1.000	Cr+PCr
6.726	2%	1.197	Glu+Gln
2.132	19%	0.380	Lip13a
0.000	999%	0.000	Lip13b
0.730	26%	0.130	Lip09
6.644	7%	1.183	MM09
0.945	19%	0.168	Lip20
4.754	10%	0.846	MM20
2.205	14%	0.393	MM12
4.712	10%	0.839	MM14
3.738	10%	0.665	MM17
2.132	19%	0.380	Lip13a+Lip13b
9.050	8%	1.611	MM14+Lip13a+L
7.374	5%	1.313	MM09+Lip09
5.699	9%	1.014	MM20+Lip20

DIAGNOSTICS

1 info STARTV 24
1 info FINOUT 9
Doing Water-Scaling

MISCELLANEOUS OUTPUT

FWHM = 0.035 ppm S/N = 61
Data shift = 0.027 ppm
Ph: 3 deg -19.5 deg/ppm

C-18yo-Female-Post-checkerboard

LCModel (Version 6.3-0B) Copyright: S.W. Provencher. Ref.: Magn. Reson. Med. 30:672-679 (1993). 31-July-2018 13:54

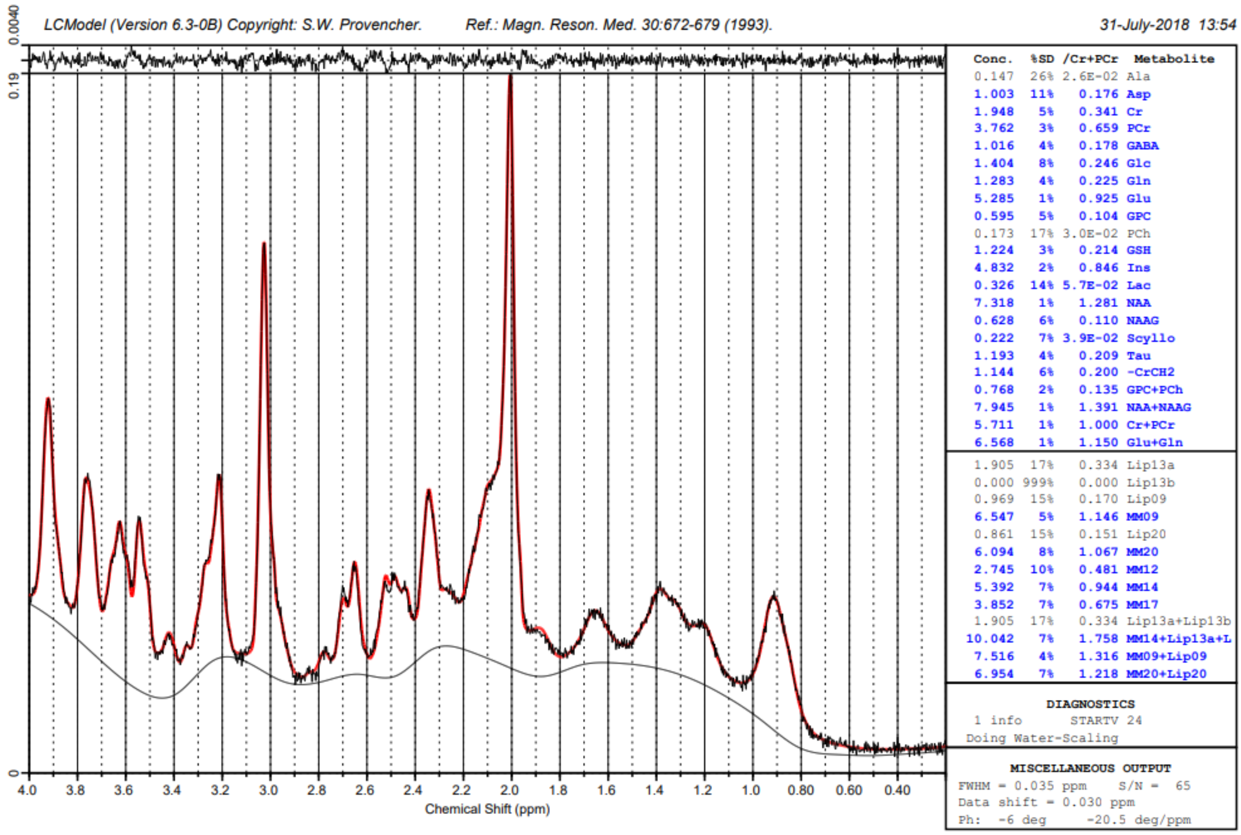

Conc.	%SD	/Cr+PCr	Metabolite
0.147	26%	2.6E-02	Ala
1.003	11%	0.176	Asp
1.948	5%	0.341	Cr
3.762	3%	0.659	PCr
1.016	4%	0.178	GABA
1.404	8%	0.246	Glc
1.283	4%	0.225	Gln
5.285	1%	0.925	Glu
0.595	5%	0.104	GPC
0.173	17%	3.0E-02	PCh
1.224	3%	0.214	GSH
4.832	2%	0.846	Ins
0.326	14%	5.7E-02	Lac
7.318	1%	1.281	NAA
0.628	6%	0.110	NAAG
0.222	7%	3.9E-02	Scyllo
1.193	4%	0.209	Tau
1.144	6%	0.200	-CrCH2
0.768	2%	0.135	GPC+PCh
7.945	1%	1.391	NAA+NAAG
5.711	1%	1.000	Cr+PCr
6.568	1%	1.150	Glu+Gln
1.905	17%	0.334	Lip13a
0.000	999%	0.000	Lip13b
0.969	15%	0.170	Lip09
6.547	5%	1.146	MM09
0.861	15%	0.151	Lip20
6.094	8%	1.067	MM20
2.745	10%	0.481	MM12
5.392	7%	0.944	MM14
3.852	7%	0.675	MM17
1.905	17%	0.334	Lip13a+Lip13b
10.042	7%	1.758	MM14+Lip13a+L
7.516	4%	1.316	MM09+Lip09
6.954	7%	1.218	MM20+Lip20

DIAGNOSTICS

1 info STARTV 24
Doing Water-Scaling

MISCELLANEOUS OUTPUT

FWHM = 0.035 ppm S/N = 65
Data shift = 0.030 ppm
Ph: -6 deg -20.5 deg/ppm

MIGRAINE WITH AURA-45yo-Male-Pre-checkerboard

(1985) Series/Acq=13/1 (2017.11.17 10:27) Pre Checkerboard TR/TE/NS=8590/6/32, 1.500E+01mL (M 045Y, 66kg) MBCIU PROTOCOLS 7T-2017.007 Migraine MRS (MBCIU) _c_32

Data of: Radiology Department, The Royal Melbourne Hospital

LCModel (Version 6.3-0B) Copyright: S.W. Provencher. Ref.: Magn. Reson. Med. 30:672-679 (1993).

31-July-2018 13:59

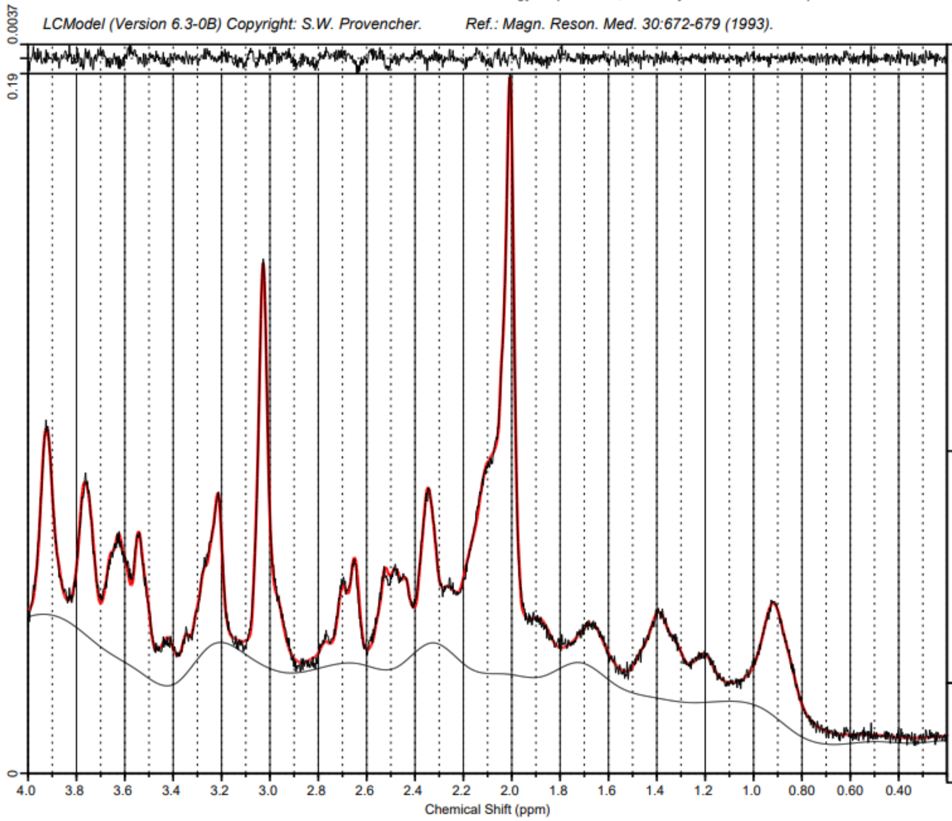

Conc.	%SD	/Cr+PCr	Metabolite
0.202	26%	3.7E-02	Ala
0.645	19%	0.118	Asp
1.890	9%	0.345	Cr
3.586	5%	0.655	PCr
0.859	6%	0.157	GABA
1.009	14%	0.184	Glc
1.395	4%	0.255	Gln
4.827	2%	0.881	Glu
0.643	3%	0.117	GPC
0.000	999%	0.000	PCh
1.067	4%	0.195	GSH
3.978	3%	0.726	Ins
0.287	23%	5.2E-02	Lac
6.847	2%	1.250	NAA
1.037	7%	0.189	NAAG
0.227	8%	4.1E-02	Scyllo
0.887	7%	0.162	Tau
1.089	8%	0.199	-CrCH2
0.643	3%	0.117	GPC+PCh
7.885	2%	1.440	NAA+NAAG
5.476	1%	1.000	Cr+PCr
6.222	2%	1.136	Glu+Gln
0.727	56%	0.133	Lip13a
0.000	999%	0.000	Lip13b
0.396	43%	7.2E-02	Lip09
6.111	9%	1.116	MM09
0.560	17%	0.102	Lip20
12.492	12%	2.281	MM20
1.996	20%	0.364	MM12
5.937	15%	1.084	MM14
2.995	20%	0.547	MM17
0.727	56%	0.133	Lip13a+Lip13b
8.659	14%	1.581	MM14+Lip13a+L
6.507	8%	1.188	MM09+Lip09
13.052	11%	2.384	MM20+Lip20

DIAGNOSTICS

1 info STARTV 24
1 info RFALSI 12
3 info's RFALSI 11
Doing Water-Scaling

MISCELLANEOUS OUTPUT

FWHM = 0.035 ppm S/N = 62
Data shift = 0.030 ppm

MIGRAINE WITH AURA-45yo-Male-Post-checkerboard

LCModel (Version 6.3-0B) Copyright: S.W. Provencher. Ref.: Magn. Reson. Med. 30:672-679 (1993). 31-July-2018 13:59

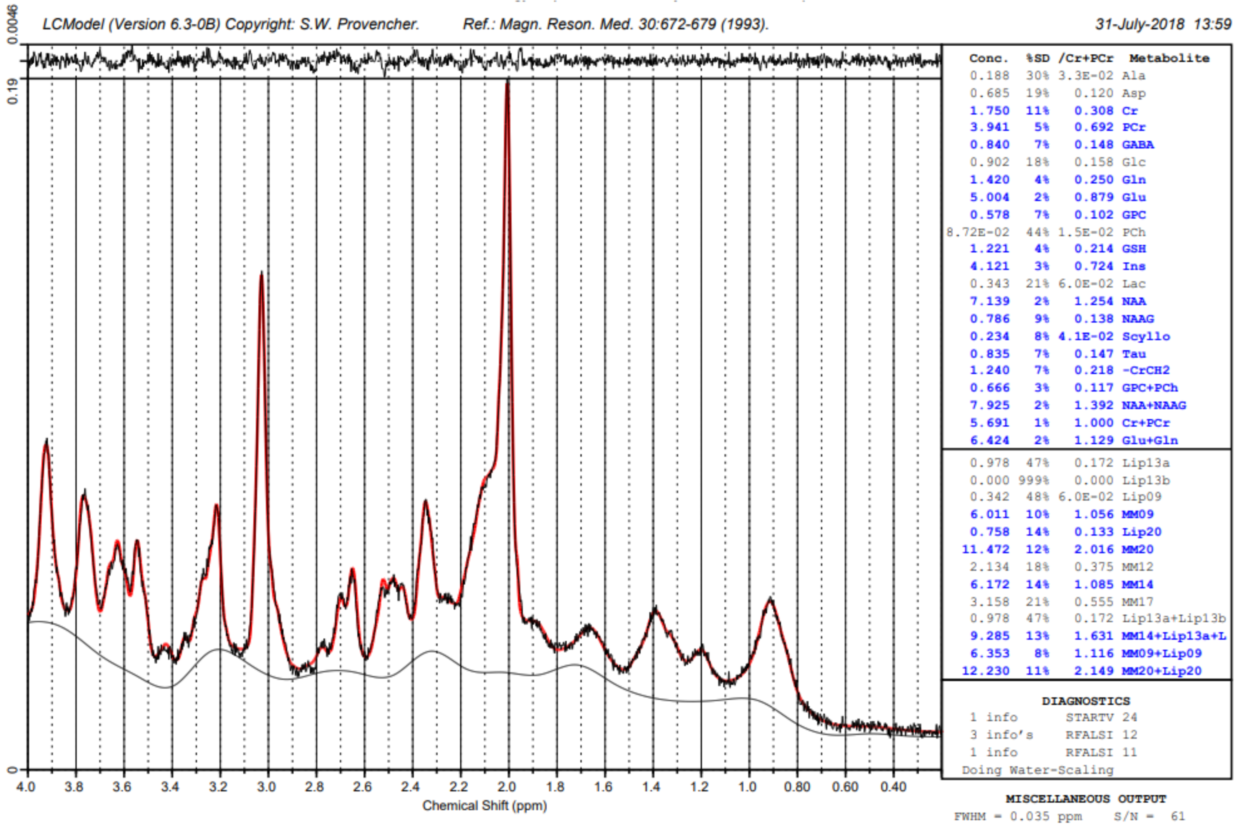

Conc.	%SD	/Cr+PCr	Metabolite
0.188	30%	3.3E-02	Ala
0.685	19%	0.120	Asp
1.750	11%	0.308	Cr
3.941	5%	0.692	PCr
0.840	7%	0.148	GABA
0.902	18%	0.158	Glc
1.420	4%	0.250	Gln
5.004	2%	0.879	Glu
0.578	7%	0.102	GPC
8.72E-02	44%	1.5E-02	PCh
1.221	4%	0.214	GSH
4.121	3%	0.724	Ins
0.343	21%	6.0E-02	Lac
7.139	2%	1.254	NAA
0.786	9%	0.138	NAAG
0.234	8%	4.1E-02	Scyllo
0.835	7%	0.147	Tau
1.240	7%	0.218	-CrCH2
0.666	3%	0.117	GPC+PCh
7.925	2%	1.392	NAA+NAAG
5.691	1%	1.000	Cr+PCr
6.424	2%	1.129	Glu+Gln
0.978	47%	0.172	Lip13a
0.000	999%	0.000	Lip13b
0.342	48%	6.0E-02	Lip09
6.011	10%	1.056	MM09
0.758	14%	0.133	Lip20
11.472	12%	2.016	MM20
2.134	18%	0.375	MM12
6.172	14%	1.085	MM14
3.158	21%	0.555	MM17
0.978	47%	0.172	Lip13a+Lip13b
9.285	13%	1.631	MM14+Lip13a+L
6.353	8%	1.116	MM09+Lip09
12.230	11%	2.149	MM20+Lip20

DIAGNOSTICS

1 info	STARTV 24
3 info's	RFALSI 12
1 info	RFALSI 11

Doing Water-Scaling

MISCELLANEOUS OUTPUT

FWHM = 0.035 ppm S/N = 61

Data shift = 0.027 ppm

MIGRAINE WITHOUT AURA-29yo-Male-Pre-checkerboard

LCModel (Version 6.3-0B) Copyright: S.W. Provencher. *Ref.: Magn. Reson. Med. 30:672-679 (1993).* *31-July-2018 14:00*

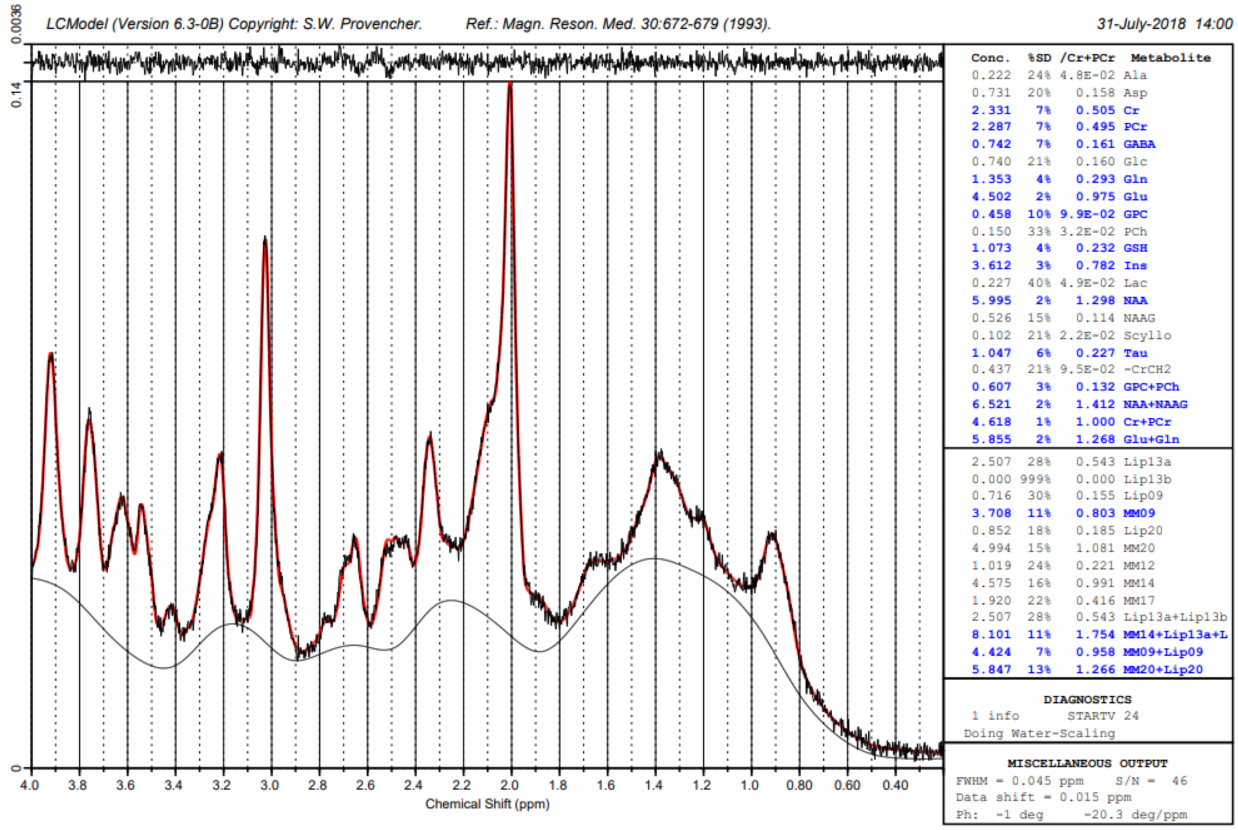

Conc.	%SD	/Cr+PCr	Metabolite
0.222	24%	4.8E-02	Ala
0.731	20%	0.158	Asp
**2.331**	**7%**	**0.505**	**Cr**
**2.287**	**7%**	**0.495**	**PCr**
**0.742**	**7%**	**0.161**	**GABA**
0.740	21%	0.160	Glc
**1.353**	**4%**	**0.293**	**Gln**
**4.502**	**2%**	**0.975**	**Glu**
**0.458**	**10%**	**9.9E-02**	**GPC**
0.150	33%	3.2E-02	PCh
**1.073**	**4%**	**0.232**	**GSH**
**3.612**	**3%**	**0.782**	**Ins**
0.227	40%	4.9E-02	Lac
**5.995**	**2%**	**1.298**	**NAA**
0.526	15%	0.114	NAAG
0.102	21%	2.2E-02	Scyllo
**1.047**	**6%**	**0.227**	**Tau**
0.437	21%	9.5E-02	-CrCH2
**0.607**	**3%**	**0.132**	**GPC+PCh**
**6.521**	**2%**	**1.412**	**NAA+NAAG**
**4.618**	**1%**	**1.000**	**Cr+PCr**
**5.855**	**2%**	**1.268**	**Glu+Gln**
2.507	28%	0.543	Lip13a
0.000	999%	0.000	Lip13b
0.716	30%	0.155	Lip09
**3.708**	**11%**	**0.803**	**MM09**
0.852	18%	0.185	Lip20
4.994	15%	1.081	MM20
1.019	24%	0.221	MM12
4.575	16%	0.991	MM14
1.920	22%	0.416	MM17
2.507	28%	0.543	Lip13a+Lip13b
**8.101**	**11%**	**1.754**	**MM14+Lip13a+L**
**4.424**	**7%**	**0.958**	**MM09+Lip09**
**5.847**	**13%**	**1.266**	**MM20+Lip20**

DIAGNOSTICS

1 info STARTV 24
Doing Water-Scaling

MISCELLANEOUS OUTPUT

FWHM = 0.045 ppm S/N = 46
Data shift = 0.015 ppm
Ph: -1 deg -20.3 deg/ppm

MIGRAINE WITHOUT AURA-29yo-Male-Post-checkerboard

LCModel (Version 6.3-0B) Copyright: S.W. Provencher. *Ref.: Magn. Reson. Med. 30:672-679 (1993).* *31-July-2018 13:59*

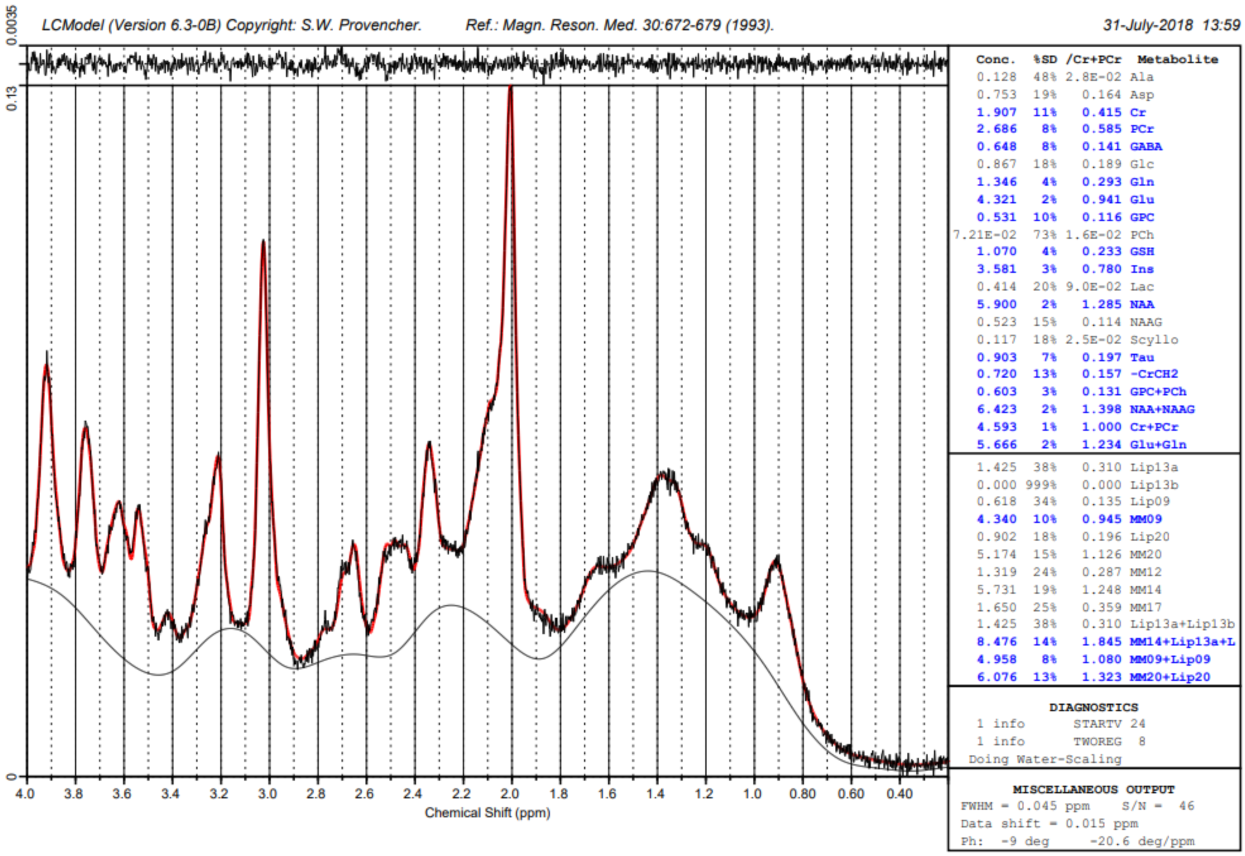

Conc.	%SD	/Cr+PCr	Metabolite
0.128	48%	2.8E-02	Ala
0.753	19%	0.164	Asp
1.907	11%	0.415	Cr
2.686	8%	0.585	PCr
0.648	8%	0.141	GABA
0.867	18%	0.189	Glc
1.346	4%	0.293	Gln
4.321	2%	0.941	Glu
0.531	10%	0.116	GPC
7.21E-02	73%	1.6E-02	PCh
1.070	4%	0.233	GSH
3.581	3%	0.780	Ins
0.414	20%	9.0E-02	Lac
5.900	2%	1.285	NAA
0.523	15%	0.114	NAAG
0.117	18%	2.5E-02	Scyllo
0.903	7%	0.197	Tau
0.720	13%	0.157	-CrCH2
0.603	3%	0.131	GPC+PCh
6.423	2%	1.398	NAA+NAAG
4.593	1%	1.000	Cr+PCr
5.666	2%	1.234	Glu+Gln
1.425	38%	0.310	Lip13a
0.000	999%	0.000	Lip13b
0.618	34%	0.135	Lip09
4.340	10%	0.945	MM09
0.902	18%	0.196	Lip20
5.174	15%	1.126	MM20
1.319	24%	0.287	MM12
5.731	19%	1.248	MM14
1.650	25%	0.359	MM17
1.425	38%	0.310	Lip13a+Lip13b
8.476	14%	1.845	MM14+Lip13a+L
4.958	8%	1.080	MM09+Lip09
6.076	13%	1.323	MM20+Lip20

DIAGNOSTICS

1 info STARTV 24

1 info TWOREG 8

Doing Water-Scaling

MISCELLANEOUS OUTPUT

FWHM = 0.045 ppm S/N = 46

Data shift = 0.015 ppm

Ph: -9 deg -20.6 deg/ppm

MIGRAINE WITH AURA-25yo-Female-Pre-checkerboard

LCModel (Version 6.3-0B) Copyright: S.W. Provencher. *Ref.: Magn. Reson. Med. 30:672-679 (1993).* *31-July-2018 14:00*

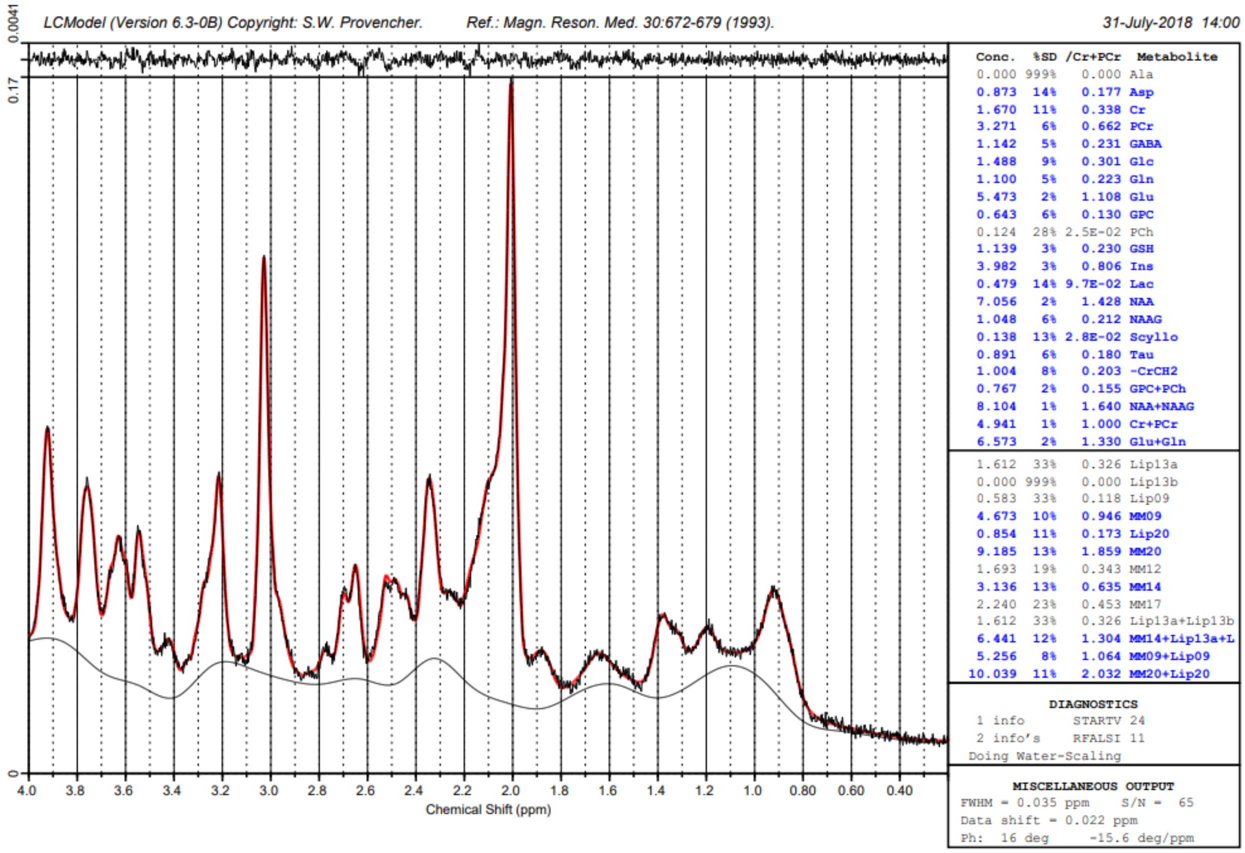

Conc.	%SD	/Cr+PCr	Metabolite
0.000	999%	0.000	Ala
0.873	14%	0.177	Asp
1.670	11%	0.338	Cr
3.271	6%	0.662	PCr
1.142	5%	0.231	GABA
1.488	9%	0.301	Glc
1.100	5%	0.223	Gln
5.473	2%	1.108	Glu
0.643	6%	0.130	GPC
0.124	28%	2.5E-02	PCh
1.139	3%	0.230	GSH
3.982	3%	0.806	Ins
0.479	14%	9.7E-02	Lac
7.056	2%	1.428	NAA
1.048	6%	0.212	NAAG
0.138	13%	2.8E-02	Scyllo
0.891	6%	0.180	Tau
1.004	8%	0.203	-CrCH2
0.767	2%	0.155	GPC+PCh
8.104	1%	1.640	NAA+NAAG
4.941	1%	1.000	Cr+PCr
6.573	2%	1.330	Glu+Gln
1.612	33%	0.326	Lip13a
0.000	999%	0.000	Lip13b
0.583	33%	0.118	Lip09
4.673	10%	0.946	MM09
0.854	11%	0.173	Lip20
9.185	13%	1.859	MM20
1.693	19%	0.343	MM12
3.136	13%	0.635	MM14
2.240	23%	0.453	MM17
1.612	33%	0.326	Lip13a+Lip13b
6.441	12%	1.304	MM14+Lip13a+L
5.256	8%	1.064	MM09+Lip09
10.039	11%	2.032	MM20+Lip20

DIAGNOSTICS

1 info STARTV 24
2 info's RFALSI 11
Doing Water-Scaling

MISCELLANEOUS OUTPUT

FWHM = 0.035 ppm S/N = 65
Data shift = 0.022 ppm
Ph: 16 deg -15.6 deg/ppm

MIGRAINE WITH AURA-25yo-Female-Post-checkerboard

LCModel (Version 6.3-0B) Copyright: S.W. Provencher. *Ref.: Magn. Reson. Med. 30:672-679 (1993).* *31-July-2018 14:00*

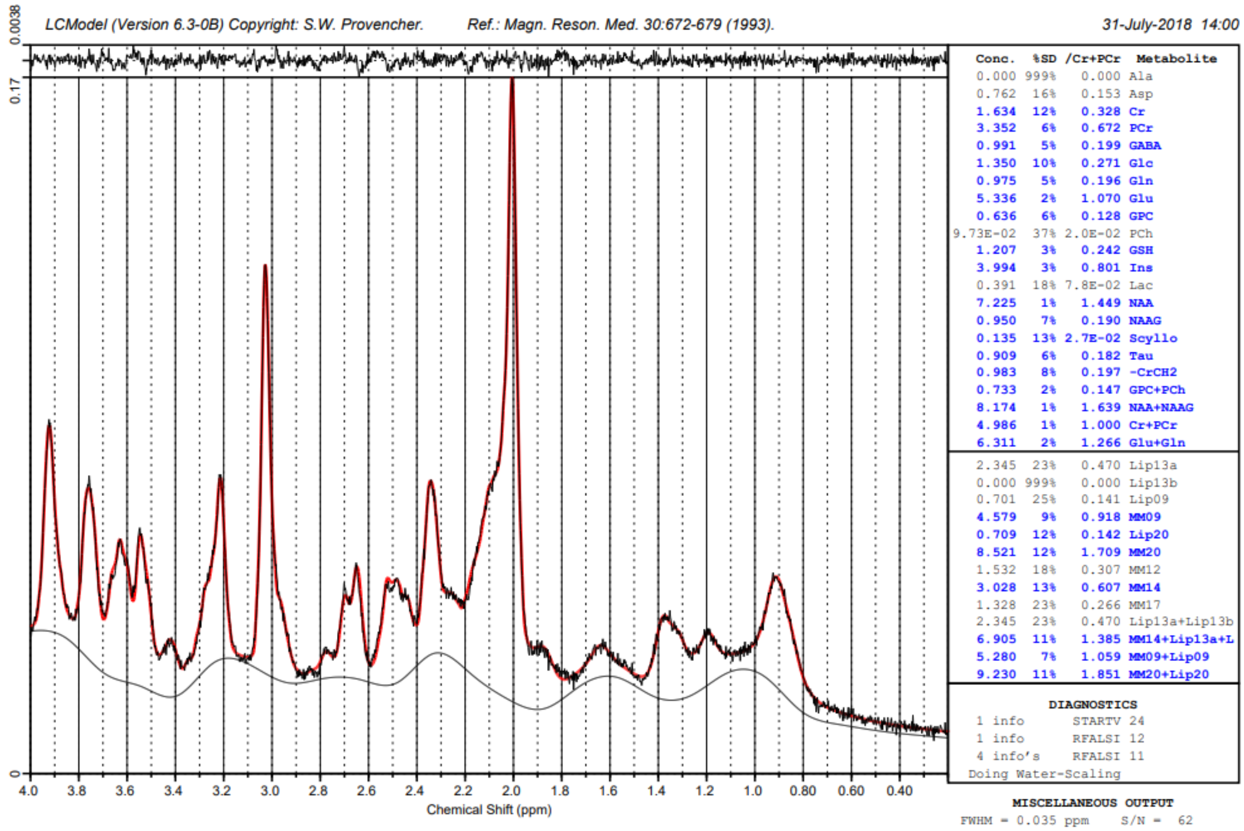

Conc.	%SD	/Cr+PCr	Metabolite
0.000	999%	0.000	Ala
0.762	16%	0.153	Asp
1.634	12%	0.328	Cr
3.352	6%	0.672	PCr
0.991	5%	0.199	GABA
1.350	10%	0.271	Glc
0.975	5%	0.196	Gln
5.336	2%	1.070	Glu
0.636	6%	0.128	GPC
9.73E-02	37%	2.0E-02	PCh
1.207	3%	0.242	GSH
3.994	3%	0.801	Ins
0.391	18%	7.8E-02	Lac
7.225	1%	1.449	NAA
0.950	7%	0.190	NAAG
0.135	13%	2.7E-02	Scyllo
0.909	6%	0.182	Tau
0.983	8%	0.197	-CrCH2
0.733	2%	0.147	GPC+PCh
8.174	1%	1.639	NAA+NAAG
4.986	1%	1.000	Cr+PCr
6.311	2%	1.266	Glu+Gln
2.345	23%	0.470	Lip13a
0.000	999%	0.000	Lip13b
0.701	25%	0.141	Lip09
4.579	9%	0.918	MM09
0.709	12%	0.142	Lip20
8.521	12%	1.709	MM20
1.532	18%	0.307	MM12
3.028	13%	0.607	MM14
1.328	23%	0.266	MM17
2.345	23%	0.470	Lip13a+Lip13b
6.905	11%	1.385	MM14+Lip13a+L
5.280	7%	1.059	MM09+Lip09
9.230	11%	1.851	MM20+Lip20

DIAGNOSTICS

1 info	STARTV 24
1 info	RFALSI 12
4 info's	RFALSI 11

Doing Water-Scaling

MISCELLANEOUS OUTPUT

FWHM = 0.035 ppm S/N = 62

Data shift = 0.022 ppm

C-46yo-Female-Pre-checkerboard

LCModel (Version 6.3-0B) Copyright: S.W. Provencher. *Ref.: Magn. Reson. Med. 30:672-679 (1993).* *31-July-2018 14:00*

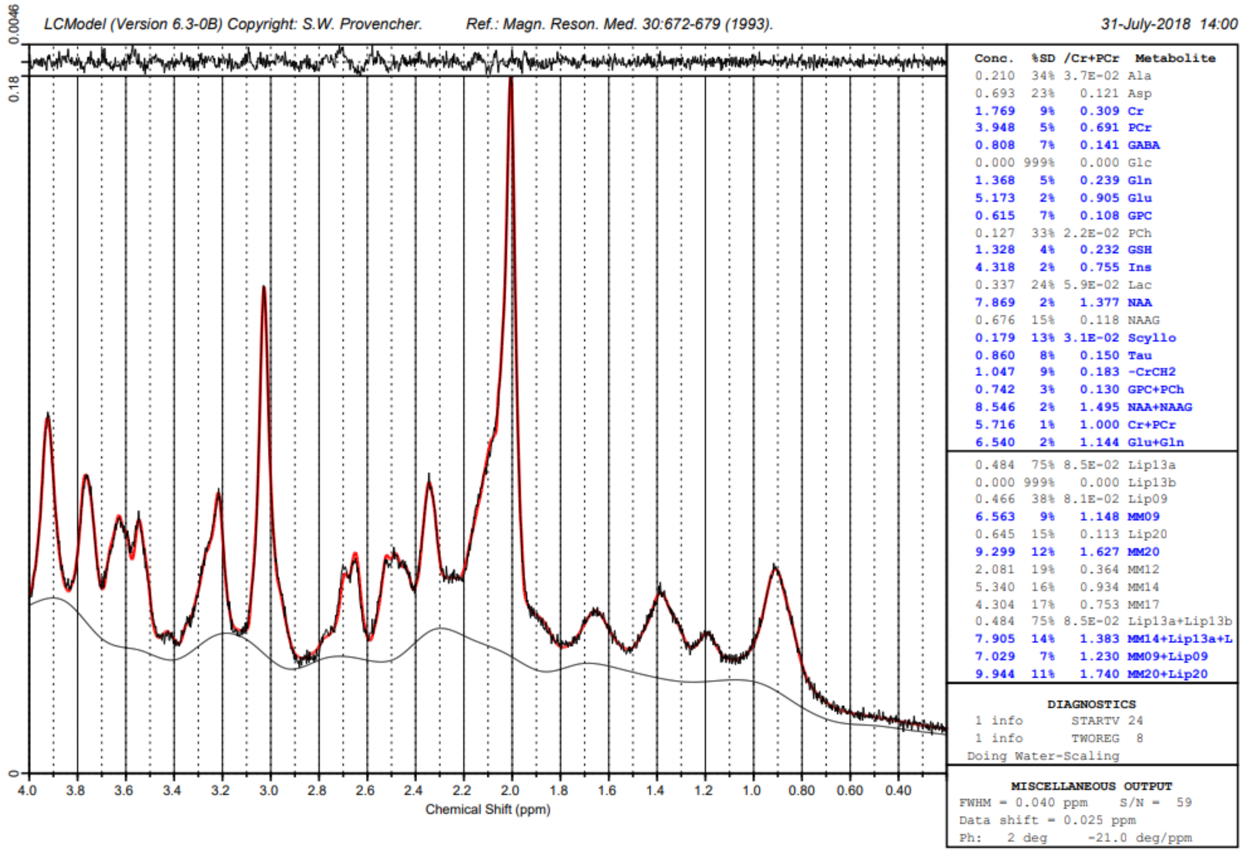

Conc.	%SD	/Cr+PCr	Metabolite
0.210	34%	3.7E-02	Ala
0.693	23%	0.121	Asp
1.769	9%	0.309	Cr
3.948	5%	0.691	PCr
0.808	7%	0.141	GABA
0.000	999%	0.000	Glc
1.368	5%	0.239	Gln
5.173	2%	0.905	Glu
0.615	7%	0.108	GPC
0.127	33%	2.2E-02	PCh
1.328	4%	0.232	GSH
4.318	2%	0.755	Ins
0.337	24%	5.9E-02	Lac
7.869	2%	1.377	NAA
0.676	15%	0.118	NAAG
0.179	13%	3.1E-02	Scyllo
0.860	8%	0.150	Tau
1.047	9%	0.183	-CrCH2
0.742	3%	0.130	GPC+PCh
8.546	2%	1.495	NAA+NAAG
5.716	1%	1.000	Cr+PCr
6.540	2%	1.144	Glu+Gln
0.484	75%	8.5E-02	Lip13a
0.000	999%	0.000	Lip13b
0.466	38%	8.1E-02	Lip09
6.563	9%	1.148	MM09
0.645	15%	0.113	Lip20
9.299	12%	1.627	MM20
2.081	19%	0.364	MM12
5.340	16%	0.934	MM14
4.304	17%	0.753	MM17
0.484	75%	8.5E-02	Lip13a+Lip13b
7.905	14%	1.383	MM14+Lip13a+L
7.029	7%	1.230	MM09+Lip09
9.944	11%	1.740	MM20+Lip20

DIAGNOSTICS

1 info STARTV 24
1 info TWOREG 8
Doing Water-Scaling

MISCELLANEOUS OUTPUT

FWHM = 0.040 ppm S/N = 59
Data shift = 0.025 ppm
Ph: 2 deg -21.0 deg/ppm

C-46yo-Female-Post-checkerboard

LCModel (Version 6.3-0B) Copyright: S.W. Provencher. Ref.: Magn. Reson. Med. 30:672-679 (1993). 31-July-2018 14:00

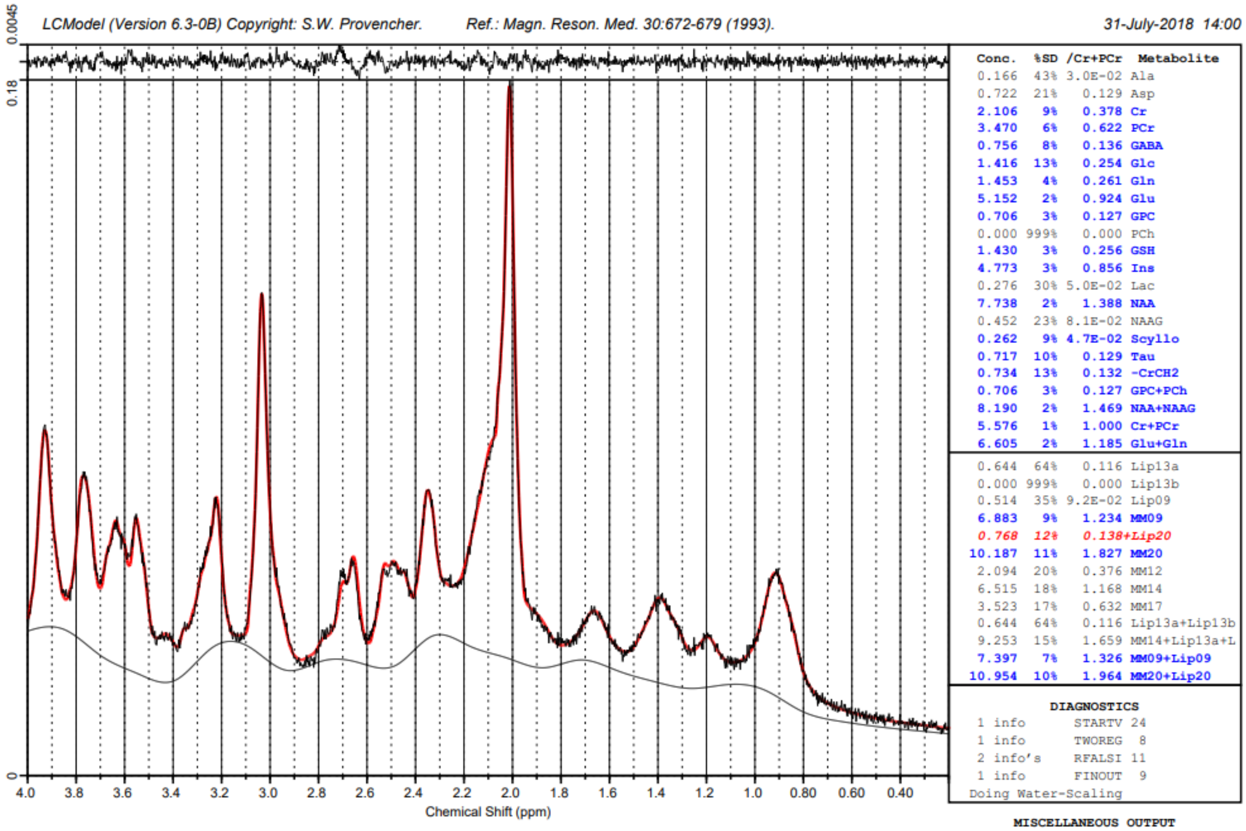

Conc.	%SD	/Cr+PCr	Metabolite
0.166	43%	3.0E-02	Ala
0.722	21%	0.129	Asp
2.106	9%	0.378	Cr
3.470	6%	0.622	PCr
0.756	8%	0.136	GABA
1.416	13%	0.254	Glc
1.453	4%	0.261	Gln
5.152	2%	0.924	Glu
0.706	3%	0.127	GPC
0.000	999%	0.000	PCh
1.430	3%	0.256	GSH
4.773	3%	0.856	Ins
0.276	30%	5.0E-02	Lac
7.738	2%	1.388	NAA
0.452	23%	8.1E-02	NAAG
0.262	9%	4.7E-02	Scyllo
0.717	10%	0.129	Tau
0.734	13%	0.132	-CrCH2
0.706	3%	0.127	GPC+PCh
8.190	2%	1.469	NAA+NAAG
5.576	1%	1.000	Cr+PCr
6.605	2%	1.185	Glu+Gln
0.644	64%	0.116	Lip13a
0.000	999%	0.000	Lip13b
0.514	35%	9.2E-02	Lip09
6.883	9%	1.234	MM09
0.768	12%	0.138	+Lip20
10.187	11%	1.827	MM20
2.094	20%	0.376	MM12
6.515	18%	1.168	MM14
3.523	17%	0.632	MM17
0.644	64%	0.116	Lip13a+Lip13b
9.253	15%	1.659	MM14+Lip13a+L
7.397	7%	1.326	MM09+Lip09
10.954	10%	1.964	MM20+Lip20

DIAGNOSTICS

1	info	STARTV	24
1	info	TWOREG	8
2	info's	RFALSI	11
1	info	FINOUT	9

Doing Water-Scaling

MISCELLANEOUS OUTPUT

FWHM = 0.040 ppm S/N = 58

C-28yo-Male-2012-Pre-checkerboard

(2012) Series/Acq=13/1 (2017.12.05 14:42) Pre Checkerboard TR/TE/NS=8500/6/32, 1.500E+01mL (M 027Y, 67kg) MBCIU PROTOCOLS 7T-2017.007 Migraine MRS (University of Melbourne - Brain Institute) _c_32

Data of: Radiology Department, The Royal Melbourne Hospital

LCModel (Version 6.3-0B) Copyright: S.W. Provencher. *Ref.: Magn. Reson. Med. 30:672-679 (1993).* *31-July-2018 14:01*

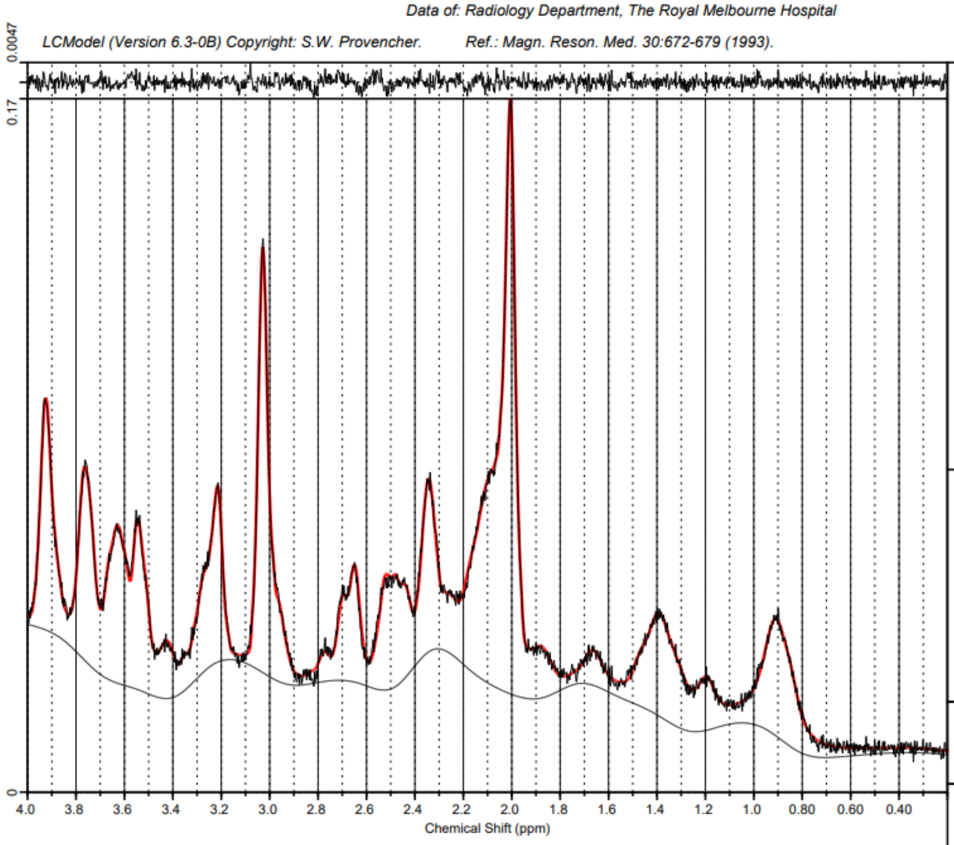

Conc.	%SD	/Cr+PCr	Metabolite
0.287	21%	5.2E-02	Ala
1.031	14%	0.186	Asp
1.572	14%	0.284	Cr
3.970	6%	0.716	PCr
0.965	6%	0.174	GABA
1.310	13%	0.236	Glc
1.384	4%	0.250	Gln
5.280	2%	0.953	Glu
0.742	3%	0.134	GPC
0.000	999%	0.000	PCh
1.421	3%	0.256	GSH
4.609	3%	0.832	Ins
0.151	49%	2.7E-02	Lac
7.460	2%	1.346	NAA
0.826	9%	0.149	NAAG
0.177	12%	3.2E-02	Scyllo
0.877	8%	0.158	Tau
1.023	9%	0.185	-CrCH2
0.742	3%	0.134	GPC+PCh
8.286	2%	1.495	NAA+NAAG
5.542	1%	1.000	Cr+PCr
6.664	2%	1.203	Glu+Gln
1.872	30%	0.338	Lip13a
0.000	999%	0.000	Lip13b
0.589	30%	0.106	Lip09
5.590	10%	1.009	MM09
0.518	17%	9.4E-02	Lip20
6.689	16%	1.207	MM20
1.582	20%	0.285	MM12
6.897	15%	1.245	MM14
1.298	21%	0.234	MM17
1.872	30%	0.338	Lip13a+Lip13b
10.351	12%	1.868	MM14+Lip13a+L
6.179	8%	1.115	MM09+Lip09
7.208	14%	1.301	MM20+Lip20

DIAGNOSTICS

1 info STARTV 24
2 info's RFALSI 12
Doing Water-Scaling

MISCELLANEOUS OUTPUT

FWHM = 0.040 ppm S/N = 54
Data shift = 0.015 ppm
Ph: 18 deg -14.9 deg/ppm

C-28yo-Male-2012-Post-checkerboard

LCModel (Version 6.3-0B) Copyright: S.W. Provencher. *Ref.: Magn. Reson. Med. 30:672-679 (1993).* *31-July-2018 14:01*

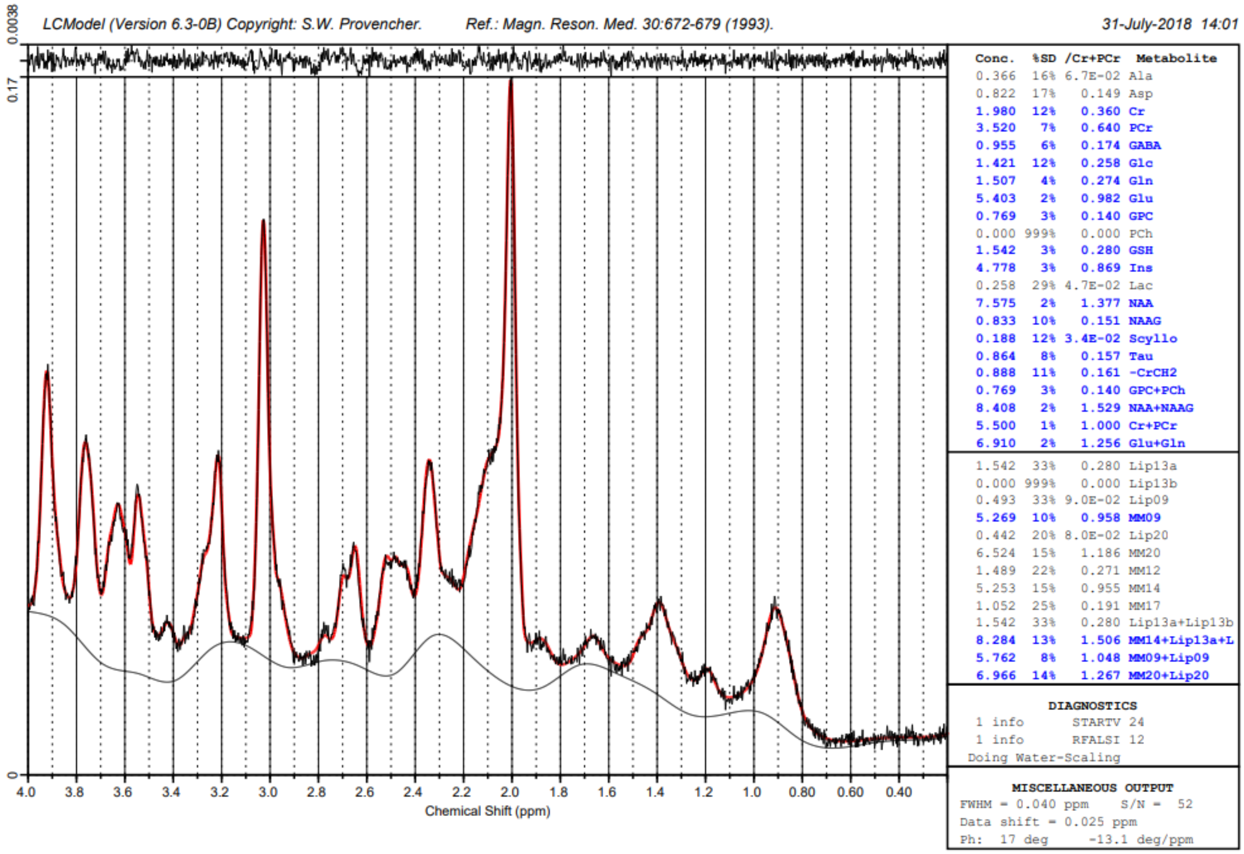

Conc.	%SD	/Cr+PCr	Metabolite
0.366	16%	6.7E-02	Ala
0.822	17%	0.149	Asp
1.980	12%	0.360	Cr
3.520	7%	0.640	PCr
0.955	6%	0.174	GABA
1.421	12%	0.258	Glc
1.507	4%	0.274	Gln
5.403	2%	0.982	Glu
0.769	3%	0.140	GPC
0.000	999%	0.000	PCh
1.542	3%	0.280	GSH
4.778	3%	0.869	Ins
0.258	29%	4.7E-02	Lac
7.575	2%	1.377	NAA
0.833	10%	0.151	NAAG
0.188	12%	3.4E-02	Scyllo
0.864	8%	0.157	Tau
0.888	11%	0.161	-CrCH2
0.769	3%	0.140	GPC+PCh
8.408	2%	1.529	NAA+NAAG
5.500	1%	1.000	Cr+PCr
6.910	2%	1.256	Glu+Gln
1.542	33%	0.280	Lip13a
0.000	999%	0.000	Lip13b
0.493	33%	9.0E-02	Lip09
5.269	10%	0.958	MM09
0.442	20%	8.0E-02	Lip20
6.524	15%	1.186	MM20
1.489	22%	0.271	MM12
5.253	15%	0.955	MM14
1.052	25%	0.191	MM17
1.542	33%	0.280	Lip13a+Lip13b
8.284	13%	1.506	MM14+Lip13a+L
5.762	8%	1.048	MM09+Lip09
6.966	14%	1.267	MM20+Lip20

DIAGNOSTICS

1 info STARTV 24

1 info RFALSI 12

Doing Water-Scaling

MISCELLANEOUS OUTPUT

FWHM = 0.040 ppm S/N = 52

Data shift = 0.025 ppm

Ph: 17 deg -13.1 deg/ppm

MIGRAINE WITH AURA-36yo-Female-Pre-checkerboard

LCModel (Version 6.3-0B) Copyright: S.W. Provencher. *Ref.: Magn. Reson. Med. 30:672-679 (1993).* *31-July-2018 14:01*

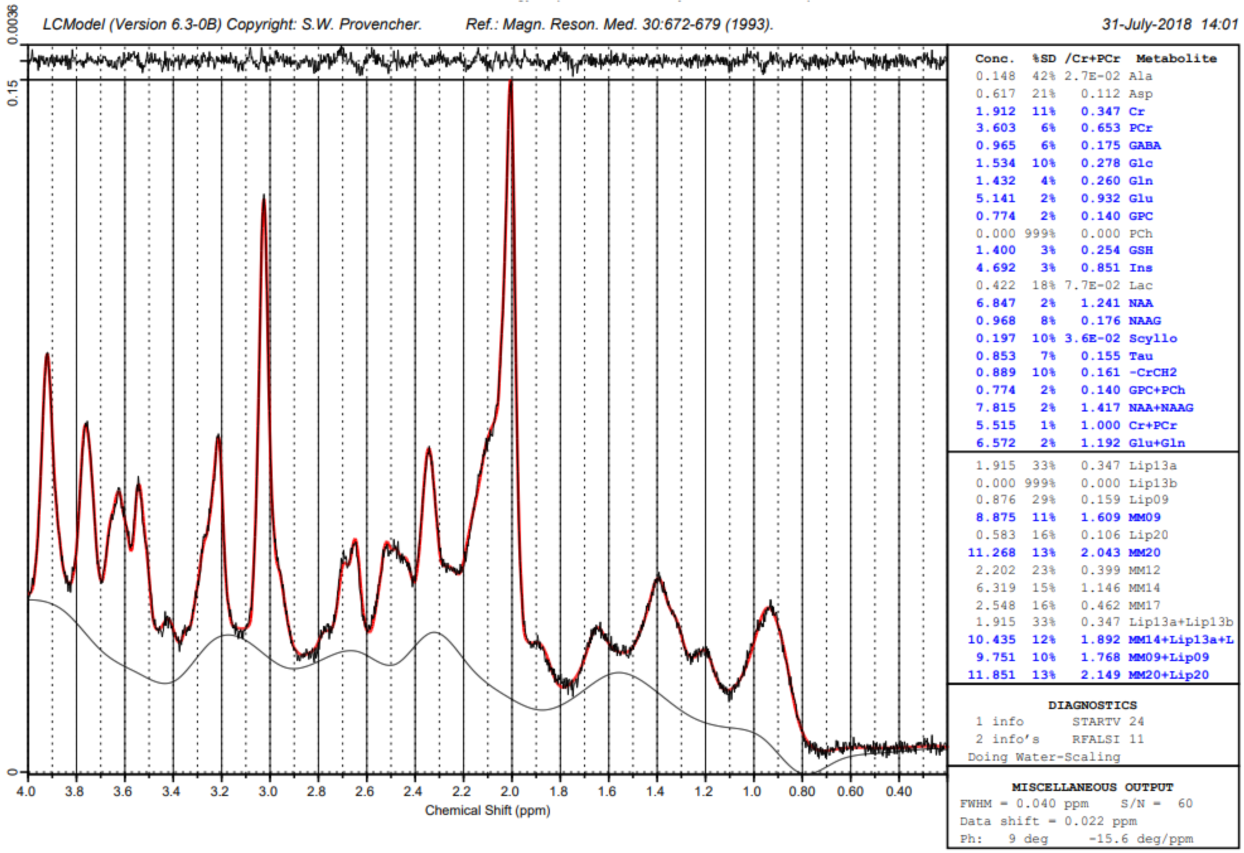

Conc.	%SD	/Cr+PCr	Metabolite
0.148	42%	2.7E-02	Ala
0.617	21%	0.112	Asp
1.912	11%	0.347	Cr
3.603	6%	0.653	PCr
0.965	6%	0.175	GABA
1.534	10%	0.278	Glc
1.432	4%	0.260	Gln
5.141	2%	0.932	Glu
0.774	2%	0.140	GPC
0.000	999%	0.000	PCh
1.400	3%	0.254	GSH
4.692	3%	0.851	Ins
0.422	18%	7.7E-02	Lac
6.847	2%	1.241	NAA
0.968	8%	0.176	NAAG
0.197	10%	3.6E-02	Scyllo
0.853	7%	0.155	Tau
0.889	10%	0.161	-CrCH2
0.774	2%	0.140	GPC+PCh
7.815	2%	1.417	NAA+NAAG
5.515	1%	1.000	Cr+PCr
6.572	2%	1.192	Glu+Gln
1.915	33%	0.347	Lip13a
0.000	999%	0.000	Lip13b
0.876	29%	0.159	Lip09
8.875	11%	1.609	MM09
0.583	16%	0.106	Lip20
11.268	13%	2.043	MM20
2.202	23%	0.399	MM12
6.319	15%	1.146	MM14
2.548	16%	0.462	MM17
1.915	33%	0.347	Lip13a+Lip13b
10.435	12%	1.892	MM14+Lip13a+L
9.751	10%	1.768	MM09+Lip09
11.851	13%	2.149	MM20+Lip20

DIAGNOSTICS

1 info STARTV 24
2 info's RFALSI 11
Doing Water-Scaling

MISCELLANEOUS OUTPUT

FWHM = 0.040 ppm S/N = 60
Data shift = 0.022 ppm
Ph: 9 deg -15.6 deg/ppm

MIGRAINE WITH AURA-36yo-Female-Post-checkerboard

LCModel (Version 6.3-0B) Copyright: S.W. Provencher. *Ref.: Magn. Reson. Med. 30:672-679 (1993).* *31-July-2018 14:01*

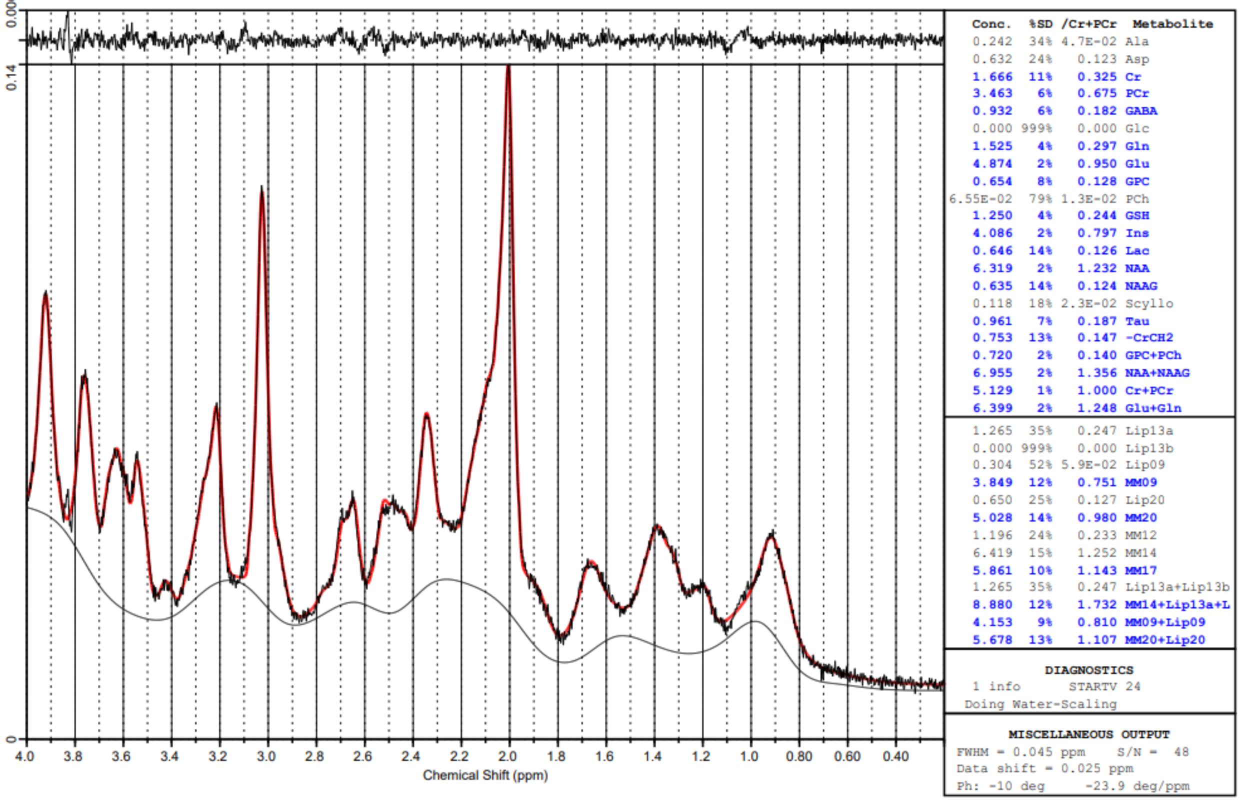

Conc.	%SD	/Cr+PCr	Metabolite
0.242	34%	4.7E-02	Ala
0.632	24%	0.123	Asp
1.666	11%	0.325	Cr
3.463	6%	0.675	PCr
0.932	6%	0.182	GABA
0.000	999%	0.000	Glc
1.525	4%	0.297	Gln
4.874	2%	0.950	Glu
0.654	8%	0.128	GPC
6.55E-02	79%	1.3E-02	PCh
1.250	4%	0.244	GSH
4.086	2%	0.797	Ins
0.646	14%	0.126	Lac
6.319	2%	1.232	NAA
0.635	14%	0.124	NAAG
0.118	18%	2.3E-02	Scyllo
0.961	7%	0.187	Tau
0.753	13%	0.147	-CrCH2
0.720	2%	0.140	GPC+PCh
6.955	2%	1.356	NAA+NAAG
5.129	1%	1.000	Cr+PCr
6.399	2%	1.248	Glu+Gln
1.265	35%	0.247	Lip13a
0.000	999%	0.000	Lip13b
0.304	52%	5.9E-02	Lip09
3.849	12%	0.751	MM09
0.650	25%	0.127	Lip20
5.028	14%	0.980	MM20
1.196	24%	0.233	MM12
6.419	15%	1.252	MM14
5.861	10%	1.143	MM17
1.265	35%	0.247	Lip13a+Lip13b
8.880	12%	1.732	MM14+Lip13a+L
4.153	9%	0.810	MM09+Lip09
5.678	13%	1.107	MM20+Lip20

DIAGNOSTICS

1 info STARTV 24

Doing Water-Scaling

MISCELLANEOUS OUTPUT

FWHM = 0.045 ppm S/N = 48

Data shift = 0.025 ppm

Ph: -10 deg -23.9 deg/ppm

CONTROL-28yo-Male-Pre-checkerboard

LCModel (Version 6.3-0B) Copyright: S.W. Provencher. *Ref.: Magn. Reson. Med. 30:672-679 (1993).* *30-July-2018 15:17*

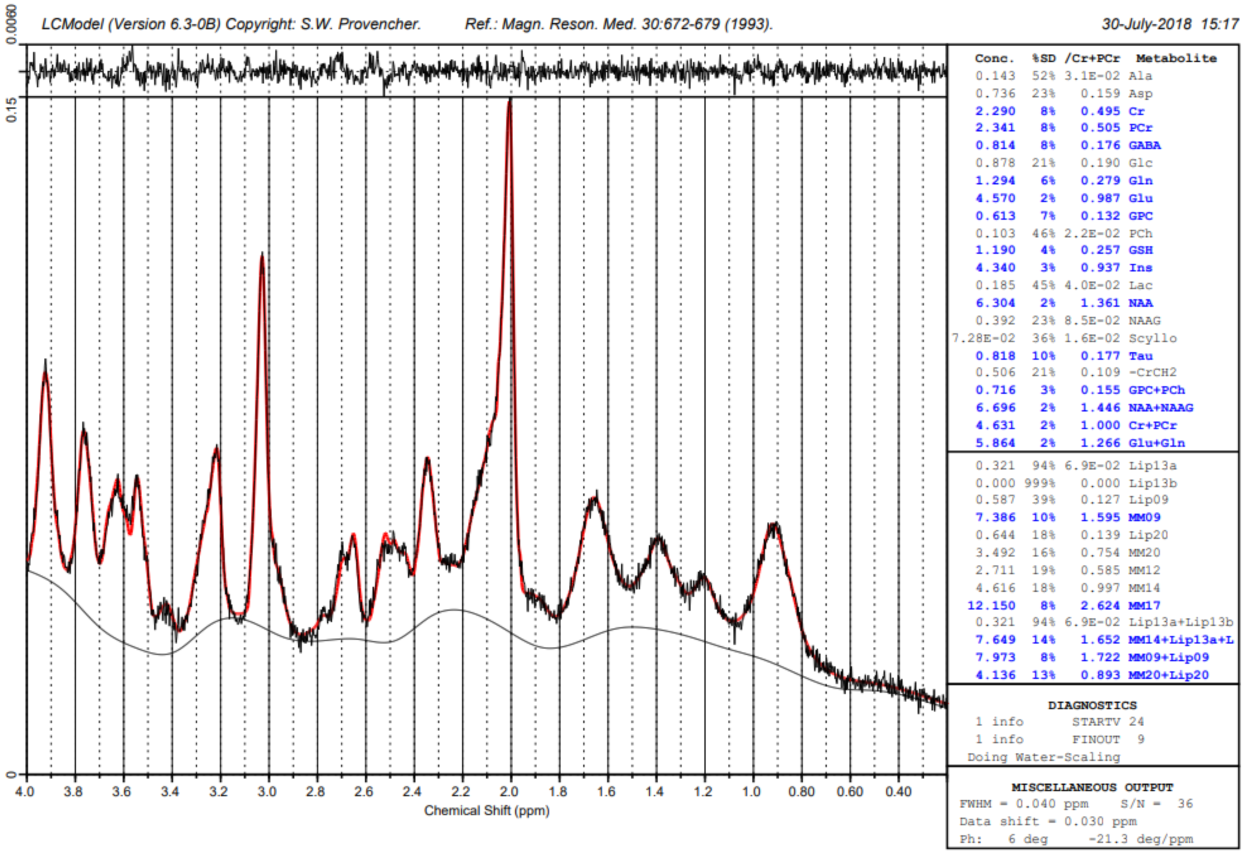

Conc.	%SD	/Cr+PCr	Metabolite
0.143	52%	3.1E-02	Ala
0.736	23%	0.159	Asp
2.290	8%	0.495	Cr
2.341	8%	0.505	PCr
0.814	8%	0.176	GABA
0.878	21%	0.190	Glc
1.294	6%	0.279	Gln
4.570	2%	0.987	Glu
0.613	7%	0.132	GPC
0.103	46%	2.2E-02	PCh
1.190	4%	0.257	GSH
4.340	3%	0.937	Ins
0.185	45%	4.0E-02	Lac
6.304	2%	1.361	NAA
0.392	23%	8.5E-02	NAAG
7.28E-02	36%	1.6E-02	Scyllo
0.818	10%	0.177	Tau
0.506	21%	0.109	-CrCH2
0.716	3%	0.155	GPC+PCh
6.696	2%	1.446	NAA+NAAG
4.631	2%	1.000	Cr+PCr
5.864	2%	1.266	Glu+Gln
0.321	94%	6.9E-02	Lip13a
0.000	999%	0.000	Lip13b
0.587	39%	0.127	Lip09
7.386	10%	1.595	MM09
0.644	18%	0.139	Lip20
3.492	16%	0.754	MM20
2.711	19%	0.585	MM12
4.616	18%	0.997	MM14
12.150	8%	2.624	MM17
0.321	94%	6.9E-02	Lip13a+Lip13b
7.649	14%	1.652	MM14+Lip13a+L
7.973	8%	1.722	MM09+Lip09
4.136	13%	0.893	MM20+Lip20

DIAGNOSTICS

1 info STARTV 24
1 info FINOUT 9
Doing Water-Scaling

MISCELLANEOUS OUTPUT

FWHM = 0.040 ppm S/N = 36
Data shift = 0.030 ppm
Ph: 6 deg -21.3 deg/ppm

CONTROL-28yo-Male-Post-checkerboard

LCModel (Version 6.3-0B) Copyright: S.W. Provencher. *Ref.: Magn. Reson. Med. 30:672-679 (1993).* *30-July-2018 15:16*

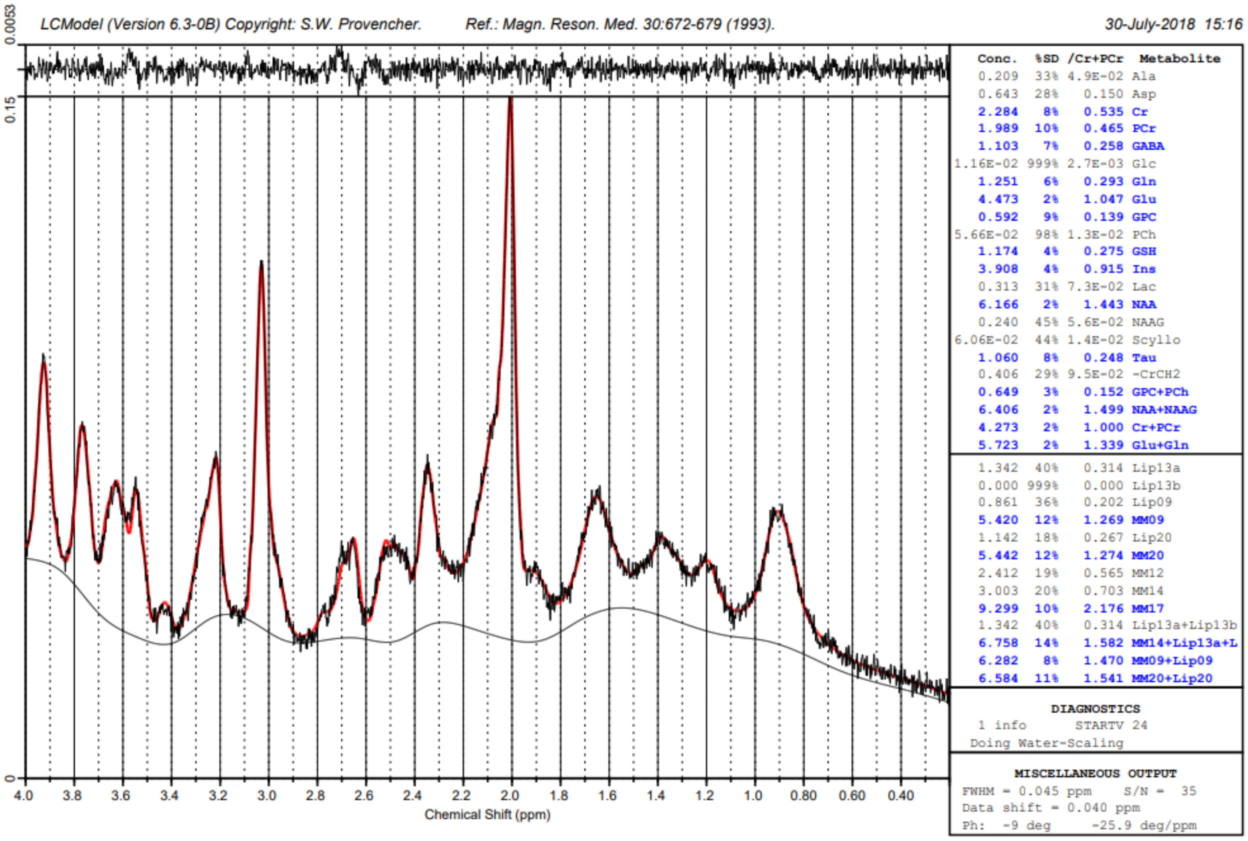

Conc.	%SD	/Cr+PCr	Metabolite
0.209	33%	4.9E-02	Ala
0.643	28%	0.150	Asp
2.284	8%	0.535	Cr
1.989	10%	0.465	PCr
1.103	7%	0.258	GABA
1.16E-02	999%	2.7E-03	Glc
1.251	6%	0.293	Gln
4.473	2%	1.047	Glu
0.592	9%	0.139	GPC
5.66E-02	98%	1.3E-02	PCh
1.174	4%	0.275	GSH
3.908	4%	0.915	Ins
0.313	31%	7.3E-02	Lac
6.166	2%	1.443	NAA
0.240	45%	5.6E-02	NAAG
6.06E-02	44%	1.4E-02	Scyllo
1.060	8%	0.248	Tau
0.406	29%	9.5E-02	-CrCH2
0.649	3%	0.152	GPC+PCh
6.406	2%	1.499	NAA+NAAG
4.273	2%	1.000	Cr+PCr
5.723	2%	1.339	Glu+Gln
1.342	40%	0.314	Lip13a
0.000	999%	0.000	Lip13b
0.861	36%	0.202	Lip09
5.420	12%	1.269	MM09
1.142	18%	0.267	Lip20
5.442	12%	1.274	MM20
2.412	19%	0.565	MM12
3.003	20%	0.703	MM14
9.299	10%	2.176	MM17
1.342	40%	0.314	Lip13a+Lip13b
6.758	14%	1.582	MM14+Lip13a+L
6.282	8%	1.470	MM09+Lip09
6.584	11%	1.541	MM20+Lip20

DIAGNOSTICS

1 info STARTV 24

Doing Water-Scaling

MISCELLANEOUS OUTPUT

FWHM = 0.045 ppm S/N = 35

Data shift = 0.040 ppm

Ph: -9 deg -25.9 deg/ppm

CONTROL-38yo-Male-Pre-checkerboard

LCModel (Version 6.3-0B) Copyright: S.W. Provencher.   *Ref.: Magn. Reson. Med. 30:672-679 (1993).*   *30-July-2018 15:57*

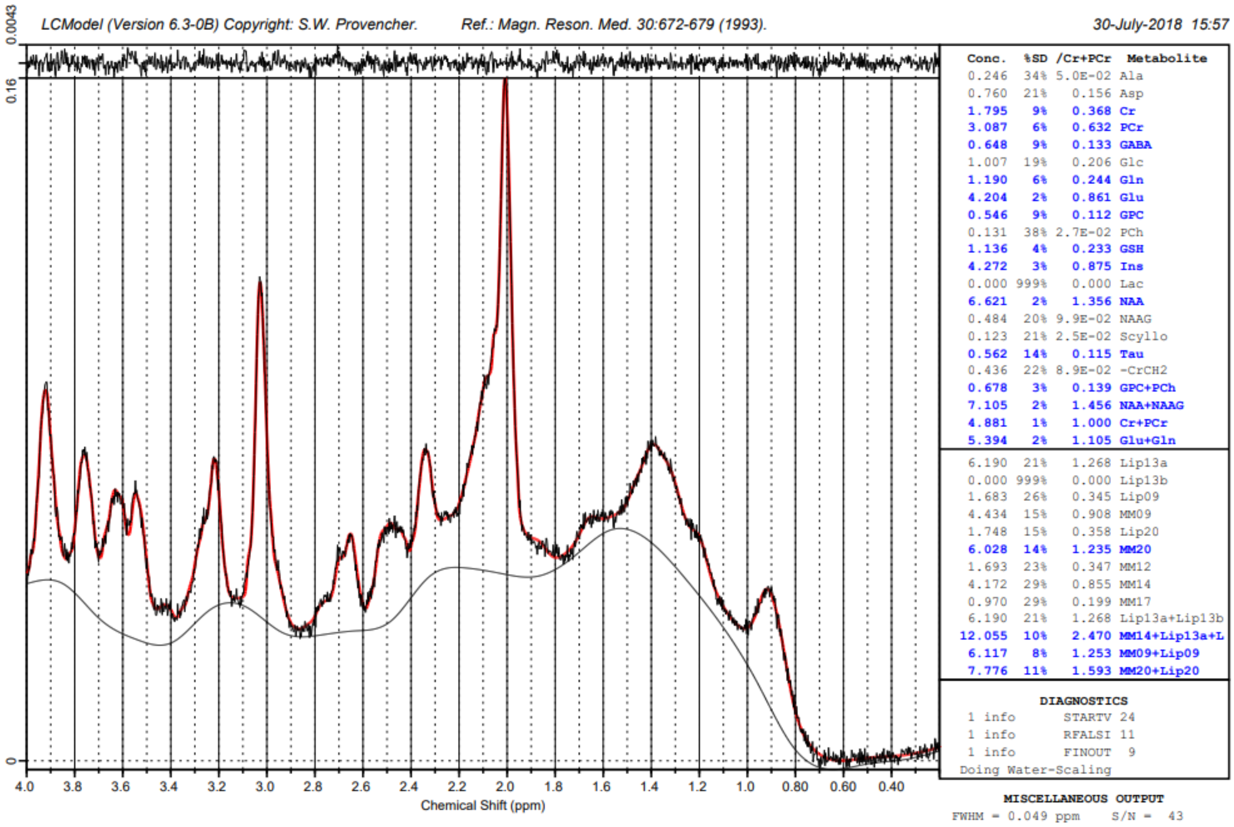

Conc.	%SD	/Cr+PCr	Metabolite
0.246	34%	5.0E-02	Ala
0.760	21%	0.156	Asp
**1.795**	**9%**	**0.368**	**Cr**
**3.087**	**6%**	**0.632**	**PCr**
**0.648**	**9%**	**0.133**	**GABA**
1.007	19%	0.206	Glc
**1.190**	**6%**	**0.244**	**Gln**
**4.204**	**2%**	**0.861**	**Glu**
**0.546**	**9%**	**0.112**	**GPC**
0.131	38%	2.7E-02	PCh
**1.136**	**4%**	**0.233**	**GSH**
**4.272**	**3%**	**0.875**	**Ins**
0.000	999%	0.000	Lac
**6.621**	**2%**	**1.356**	**NAA**
0.484	20%	9.9E-02	NAAG
0.123	21%	2.5E-02	Scyllo
**0.562**	**14%**	**0.115**	**Tau**
0.436	22%	8.9E-02	-CrCH2
**0.678**	**3%**	**0.139**	**GPC+PCh**
**7.105**	**2%**	**1.456**	**NAA+NAAG**
**4.881**	**1%**	**1.000**	**Cr+PCr**
**5.394**	**2%**	**1.105**	**Glu+Gln**
6.190	21%	1.268	Lip13a
0.000	999%	0.000	Lip13b
1.683	26%	0.345	Lip09
4.434	15%	0.908	MM09
1.748	15%	0.358	Lip20
**6.028**	**14%**	**1.235**	**MM20**
1.693	23%	0.347	MM12
4.172	29%	0.855	MM14
0.970	29%	0.199	MM17
6.190	21%	1.268	Lip13a+Lip13b
**12.055**	**10%**	**2.470**	**MM14+Lip13a+L**
**6.117**	**8%**	**1.253**	**MM09+Lip09**
**7.776**	**11%**	**1.593**	**MM20+Lip20**

DIAGNOSTICS

1 info STARTV 24
1 info RFALSI 11
1 info FINOUT 9
Doing Water-Scaling

MISCELLANEOUS OUTPUT

FWHM = 0.049 ppm   S/N = 43
Data shift = 0.022 ppm

CONTROL-38yo-Male-Post-checkerboard

LCModel (Version 6.3-0B) Copyright: S.W. Provencher. Ref.: Magn. Reson. Med. 30:672-679 (1993). 30-July-2018 15:57

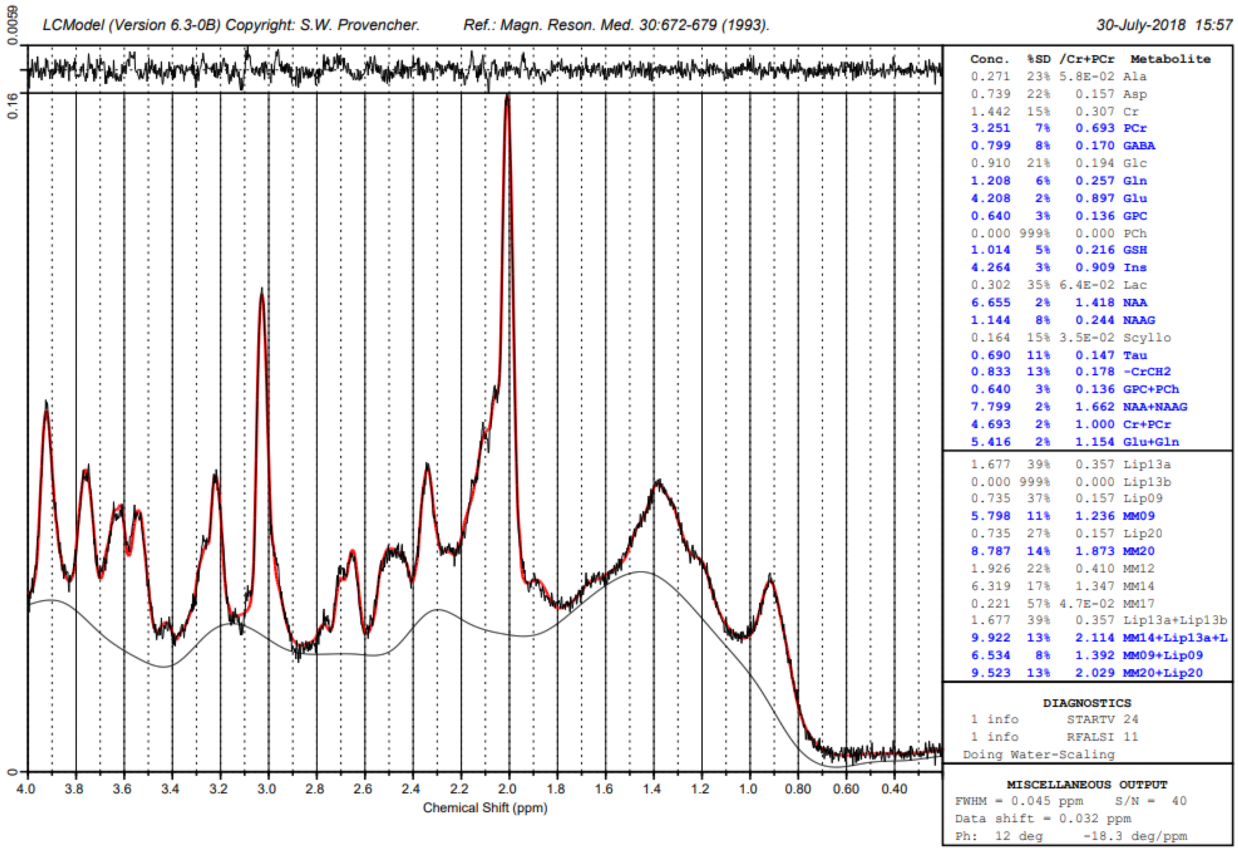

Conc.	%SD	/Cr+PCr	Metabolite
0.271	23%	5.8E-02	Ala
0.739	22%	0.157	Asp
1.442	15%	0.307	Cr
3.251	7%	0.693	PCr
0.799	8%	0.170	GABA
0.910	21%	0.194	Glc
1.208	6%	0.257	Gln
4.208	2%	0.897	Glu
0.640	3%	0.136	GPC
0.000	999%	0.000	PCh
1.014	5%	0.216	GSH
4.264	3%	0.909	Ins
0.302	35%	6.4E-02	Lac
6.655	2%	1.418	NAA
1.144	8%	0.244	NAAG
0.164	15%	3.5E-02	Scyllo
0.690	11%	0.147	Tau
0.833	13%	0.178	-CrCH2
0.640	3%	0.136	GPC+PCh
7.799	2%	1.662	NAA+NAAG
4.693	2%	1.000	Cr+PCr
5.416	2%	1.154	Glu+Gln
1.677	39%	0.357	Lip13a
0.000	999%	0.000	Lip13b
0.735	37%	0.157	Lip09
5.798	11%	1.236	MM09
0.735	27%	0.157	Lip20
8.787	14%	1.873	MM20
1.926	22%	0.410	MM12
6.319	17%	1.347	MM14
0.221	57%	4.7E-02	MM17
1.677	39%	0.357	Lip13a+Lip13b
9.922	13%	2.114	MM14+Lip13a+L
6.534	8%	1.392	MM09+Lip09
9.523	13%	2.029	MM20+Lip20

DIAGNOSTICS

1 info STARTV 24
1 info RFALSI 11
Doing Water-Scaling

MISCELLANEOUS OUTPUT

FWHM = 0.045 ppm S/N = 40
Data shift = 0.032 ppm
Ph: 12 deg -18.3 deg/ppm

CONTROL-29yo-Female-Pre-checkerboard

LCModel (Version 6.3-0B) Copyright: S.W. Provencher. *Ref.: Magn. Reson. Med. 30:672-679 (1993).* *30-July-2018 15:58*

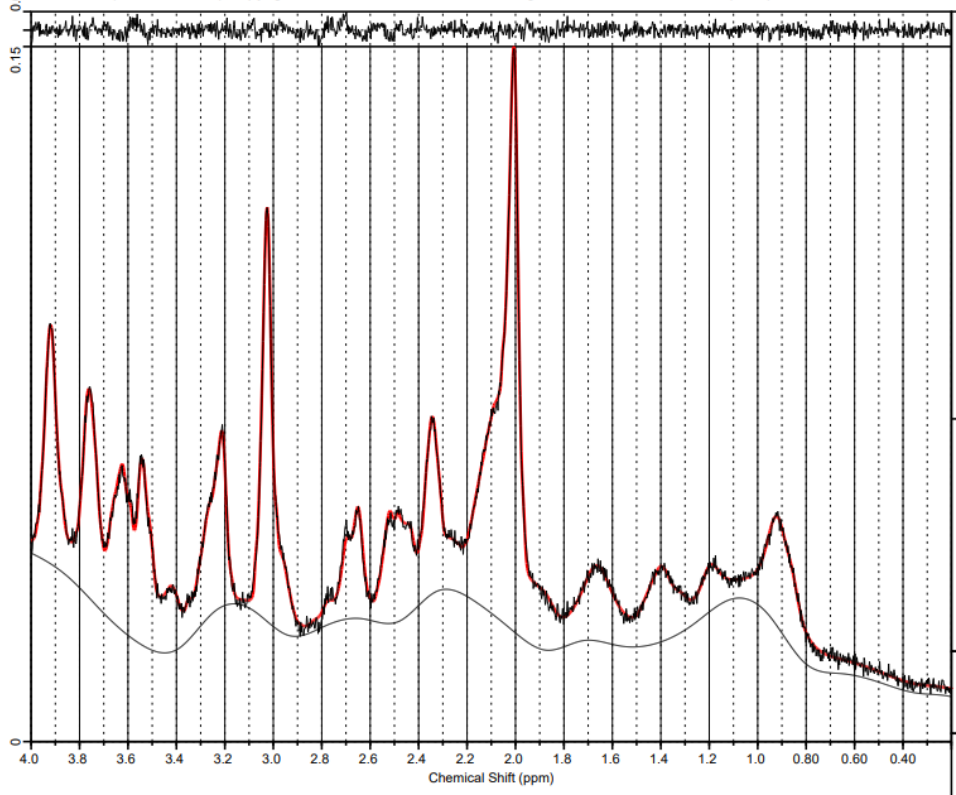

Conc.	%SD	/Cr+PCr	Metabolite
0.160	44%	3.2E-02	Ala
0.805	16%	0.163	Asp
2.445	7%	0.495	Cr
2.498	7%	0.505	PCr
0.741	7%	0.150	GABA
1.715	9%	0.347	Glc
1.371	4%	0.277	Gln
4.959	2%	1.003	Glu
0.513	8%	0.104	GPC
0.146	29%	3.0E-02	PCh
1.198	4%	0.242	GSH
4.411	3%	0.892	Ins
0.155	32%	3.1E-02	Lac
6.608	2%	1.337	NAA
0.458	16%	9.3E-02	NAAG
0.179	11%	3.6E-02	Scyllo
0.811	8%	0.164	Tau
0.559	16%	0.113	-CrCH2
0.660	3%	0.133	GPC+PCh
7.066	2%	1.430	NAA+NAAG
4.943	1%	1.000	Cr+PCr
6.329	2%	1.280	Glu+Gln
0.000	999%	0.000	Lip13a
0.000	999%	0.000	Lip13b
0.285	38%	5.8E-02	Lip09
4.413	9%	0.893	MM09
0.407	19%	8.2E-02	Lip20
6.147	12%	1.243	MM20
1.483	19%	0.300	MM12
3.675	19%	0.743	MM14
6.105	13%	1.235	MM17
0.000	999%	0.000	Lip13a+Lip13b
5.157	16%	1.043	MM14+Lip13a+L
4.698	8%	0.950	MM09+Lip09
6.553	11%	1.326	MM20+Lip20

DIAGNOSTICS

1 info STARTV 24
3 info's RFALSI 11
Doing Water-Scaling

MISCELLANEOUS OUTPUT

FWHM = 0.040 ppm S/N = 53
Data shift = 0.015 ppm
Ph: 4 deg -19.5 deg/ppm

CONTROL-29yo-Female-Post-checkerboard

LCModel (Version 6.3-0B) Copyright: S.W. Provencher. *Ref.: Magn. Reson. Med. 30:672-679 (1993).* *30-July-2018 15:58*

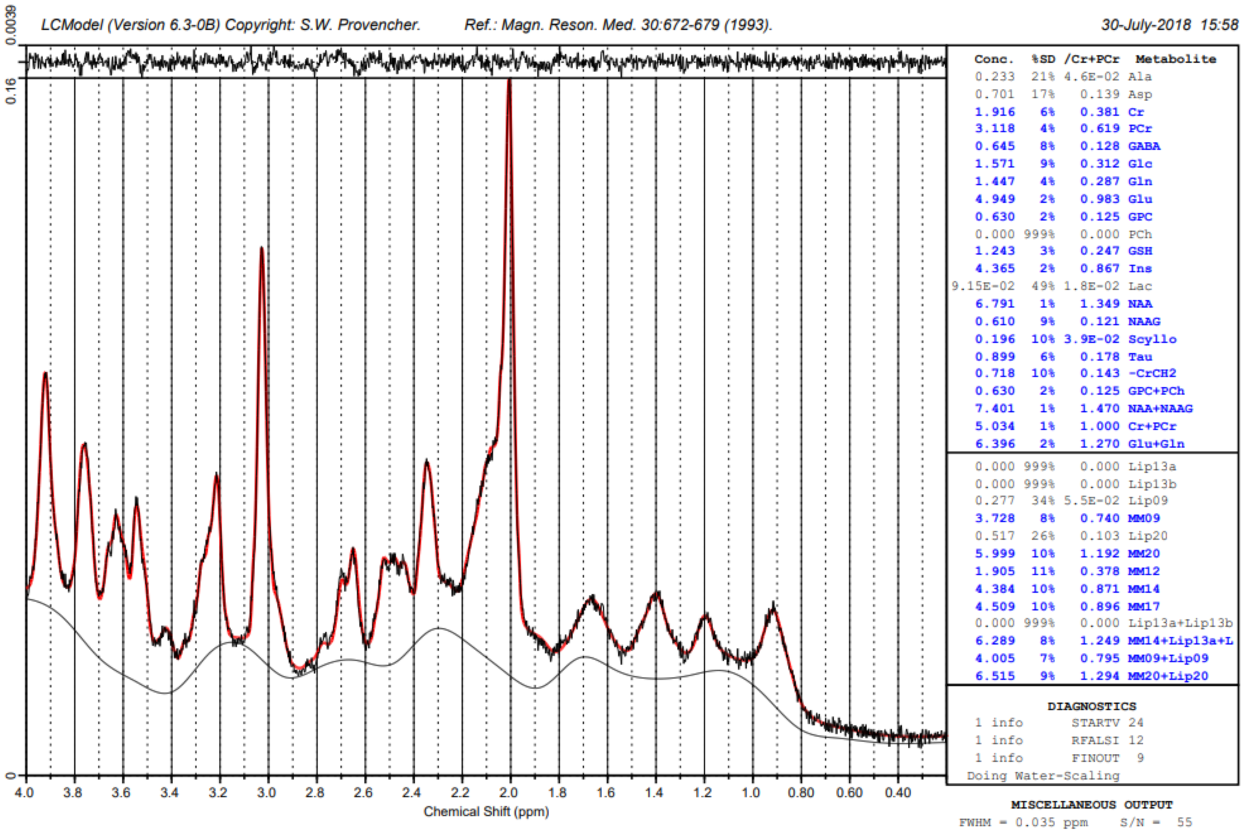

Conc.	%SD	/Cr+PCr	Metabolite
0.233	21%	4.6E-02	Ala
0.701	17%	0.139	Asp
1.916	6%	0.381	Cr
3.118	4%	0.619	PCr
0.645	8%	0.128	GABA
1.571	9%	0.312	Glc
1.447	4%	0.287	Gln
4.949	2%	0.983	Glu
0.630	2%	0.125	GPC
0.000	999%	0.000	PCh
1.243	3%	0.247	GSH
4.365	2%	0.867	Ins
9.15E-02	49%	1.8E-02	Lac
6.791	1%	1.349	NAA
0.610	9%	0.121	NAAG
0.196	10%	3.9E-02	Scyllo
0.899	6%	0.178	Tau
0.718	10%	0.143	-CrCH2
0.630	2%	0.125	GPC+PCh
7.401	1%	1.470	NAA+NAAG
5.034	1%	1.000	Cr+PCr
6.396	2%	1.270	Glu+Gln
0.000	999%	0.000	Lip13a
0.000	999%	0.000	Lip13b
0.277	34%	5.5E-02	Lip09
3.728	8%	0.740	MM09
0.517	26%	0.103	Lip20
5.999	10%	1.192	MM20
1.905	11%	0.378	MM12
4.384	10%	0.871	MM14
4.509	10%	0.896	MM17
0.000	999%	0.000	Lip13a+Lip13b
6.289	8%	1.249	MM14+Lip13a+L
4.005	7%	0.795	MM09+Lip09
6.515	9%	1.294	MM20+Lip20

DIAGNOSTICS

1 info STARTV 24
1 info RFALSI 12
1 info FINOUT 9
Doing Water-Scaling

MISCELLANEOUS OUTPUT

FWHM = 0.035 ppm S/N = 55
Data shift = 0.025 ppm

CONTROL-41yo-Male-Pre-checkerboard

LCModel (Version 6.3-0B) Copyright: S.W. Provencher. *Ref.: Magn. Reson. Med. 30:672-679 (1993).* *30-July-2018 15:58*

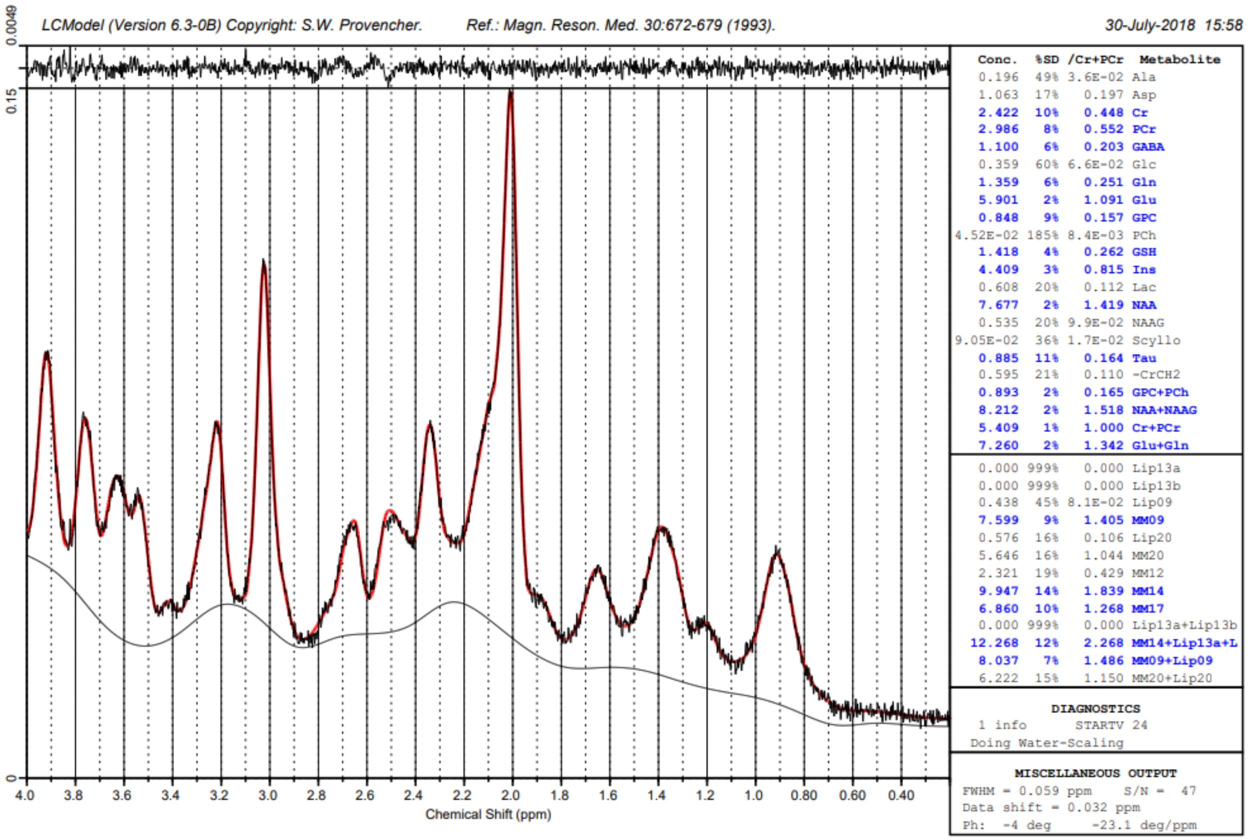

Conc.	%SD	/Cr+PCr	Metabolite
0.196	49%	3.6E-02	Ala
1.063	17%	0.197	Asp
**2.422**	**10%**	**0.448**	**Cr**
**2.986**	**8%**	**0.552**	**PCr**
**1.100**	**6%**	**0.203**	**GABA**
0.359	60%	6.6E-02	Glc
**1.359**	**6%**	**0.251**	**Gln**
**5.901**	**2%**	**1.091**	**Glu**
**0.848**	**9%**	**0.157**	**GPC**
4.52E-02	185%	8.4E-03	PCh
**1.418**	**4%**	**0.262**	**GSH**
**4.409**	**3%**	**0.815**	**Ins**
0.608	20%	0.112	Lac
**7.677**	**2%**	**1.419**	**NAA**
0.535	20%	9.9E-02	NAAG
9.05E-02	36%	1.7E-02	Scyllo
**0.885**	**11%**	**0.164**	**Tau**
0.595	21%	0.110	-CrCH2
**0.893**	**2%**	**0.165**	**GPC+PCh**
**8.212**	**2%**	**1.518**	**NAA+NAAG**
**5.409**	**1%**	**1.000**	**Cr+PCr**
**7.260**	**2%**	**1.342**	**Glu+Gln**
0.000	999%	0.000	Lip13a
0.000	999%	0.000	Lip13b
0.438	45%	8.1E-02	Lip09
**7.599**	**9%**	**1.405**	**MM09**
0.576	16%	0.106	Lip20
5.646	16%	1.044	MM20
2.321	19%	0.429	MM12
**9.947**	**14%**	**1.839**	**MM14**
**6.860**	**10%**	**1.268**	**MM17**
0.000	999%	0.000	Lip13a+Lip13b
**12.268**	**12%**	**2.268**	**MM14+Lip13a+L**
**8.037**	**7%**	**1.486**	**MM09+Lip09**
6.222	15%	1.150	MM20+Lip20

DIAGNOSTICS

1 info STARTV 24

Doing Water-Scaling

MISCELLANEOUS OUTPUT

FWHM = 0.059 ppm S/N = 47

Data shift = 0.032 ppm

Ph: -4 deg -23.1 deg/ppm

CONTROL-41yo-Male-Post-checkerboard

LCModel (Version 6.3-0B) Copyright: S.W. Provencher. *Ref.: Magn. Reson. Med. 30:672-679 (1993).* *30-July-2018 15:58*

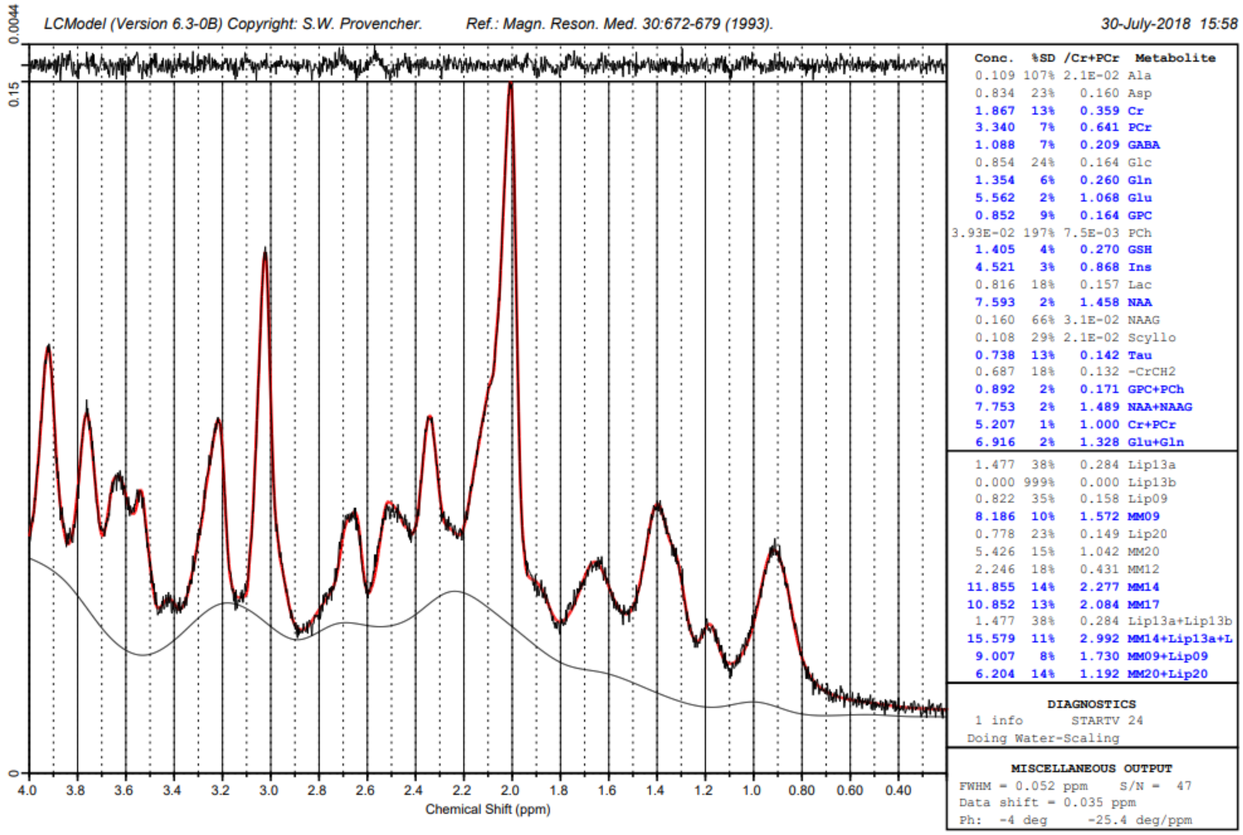

Conc.	%SD	/Cr+PCr	Metabolite
0.109	107%	2.1E-02	Ala
0.834	23%	0.160	Asp
1.867	13%	0.359	Cr
3.340	7%	0.641	PCr
1.088	7%	0.209	GABA
0.854	24%	0.164	Glc
1.354	6%	0.260	Gln
5.562	2%	1.068	Glu
0.852	9%	0.164	GPC
3.93E-02	197%	7.5E-03	PCh
1.405	4%	0.270	GSH
4.521	3%	0.868	Ins
0.816	18%	0.157	Lac
7.593	2%	1.458	NAA
0.160	66%	3.1E-02	NAAG
0.108	29%	2.1E-02	Scyllo
0.738	13%	0.142	Tau
0.687	18%	0.132	-CrCH2
0.892	2%	0.171	GPC+PCh
7.753	2%	1.489	NAA+NAAG
5.207	1%	1.000	Cr+PCr
6.916	2%	1.328	Glu+Gln
1.477	38%	0.284	Lip13a
0.000	999%	0.000	Lip13b
0.822	35%	0.158	Lip09
8.186	10%	1.572	MM09
0.778	23%	0.149	Lip20
5.426	15%	1.042	MM20
2.246	18%	0.431	MM12
11.855	14%	2.277	MM14
10.852	13%	2.084	MM17
1.477	38%	0.284	Lip13a+Lip13b
15.579	11%	2.992	MM14+Lip13a+L
9.007	8%	1.730	MM09+Lip09
6.204	14%	1.192	MM20+Lip20

DIAGNOSTICS

1 info STARTV 24

Doing Water-Scaling

MISCELLANEOUS OUTPUT

FWHM = 0.052 ppm S/N = 47

Data shift = 0.035 ppm

Ph: -4 deg -25.4 deg/ppm

CONTROL-42yo-Male-Pre-checkerboard

LCModel (Version 6.3-0B) Copyright: S.W. Provencher. *Ref.: Magn. Reson. Med. 30:672-679 (1993).* *30-July-2018 15:59*

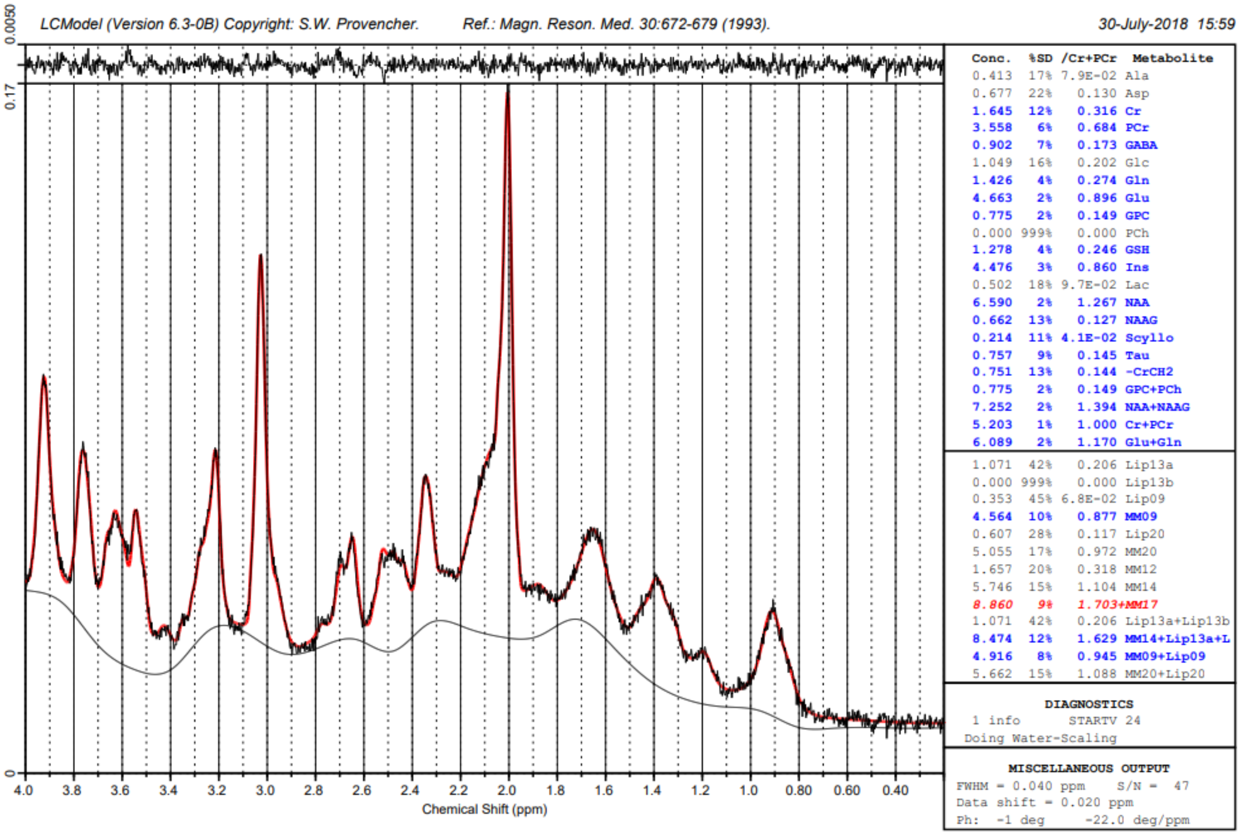

Conc.	%SD	/Cr+PCr	Metabolite
0.413	17%	7.9E-02	Ala
0.677	22%	0.130	Asp
1.645	12%	0.316	Cr
3.558	6%	0.684	PCr
0.902	7%	0.173	GABA
1.049	16%	0.202	Glc
1.426	4%	0.274	Gln
4.663	2%	0.896	Glu
0.775	2%	0.149	GPC
0.000	999%	0.000	PCh
1.278	4%	0.246	GSH
4.476	3%	0.860	Ins
0.502	18%	9.7E-02	Lac
6.590	2%	1.267	NAA
0.662	13%	0.127	NAAG
0.214	11%	4.1E-02	Scyllo
0.757	9%	0.145	Tau
0.751	13%	0.144	-CrCH2
0.775	2%	0.149	GPC+PCh
7.252	2%	1.394	NAA+NAAG
5.203	1%	1.000	Cr+PCr
6.089	2%	1.170	Glu+Gln
1.071	42%	0.206	Lip13a
0.000	999%	0.000	Lip13b
0.353	45%	6.8E-02	Lip09
4.564	10%	0.877	MM09
0.607	28%	0.117	Lip20
5.055	17%	0.972	MM20
1.657	20%	0.318	MM12
5.746	15%	1.104	MM14
8.860	9%	1.703+MM17	
1.071	42%	0.206	Lip13a+Lip13b
8.474	12%	1.629	MM14+Lip13a+L
4.916	8%	0.945	MM09+Lip09
5.662	15%	1.088	MM20+Lip20

DIAGNOSTICS

1 info STARTV 24

Doing Water-Scaling

MISCELLANEOUS OUTPUT

FWHM = 0.040 ppm S/N = 47

Data shift = 0.020 ppm

Ph: -1 deg -22.0 deg/ppm

CONTROL-42yo-Male-Post-checkerboard

LCModel (Version 6.3-0B) Copyright: S.W. Provencher.   *Ref.: Magn. Reson. Med. 30:672-679 (1993).*   *30-July-2018 15:58*

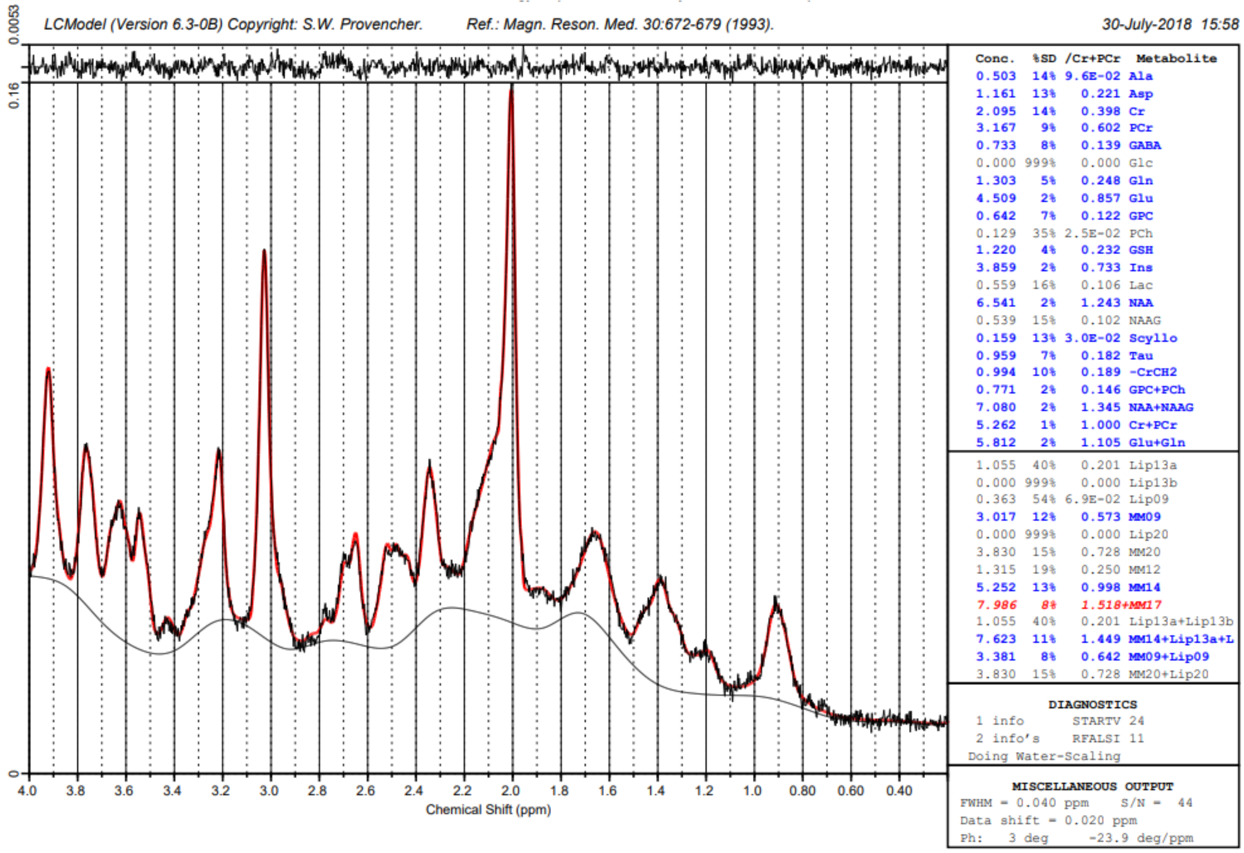

Conc.	%SD	/Cr+PCr	Metabolite
0.503	14%	9.6E-02	Ala
1.161	13%	0.221	Asp
2.095	14%	0.398	Cr
3.167	9%	0.602	PCr
0.733	8%	0.139	GABA
0.000	999%	0.000	Glc
1.303	5%	0.248	Gln
4.509	2%	0.857	Glu
0.642	7%	0.122	GPC
0.129	35%	2.5E-02	PCh
1.220	4%	0.232	GSH
3.859	2%	0.733	Ins
0.559	16%	0.106	Lac
6.541	2%	1.243	NAA
0.539	15%	0.102	NAAG
0.159	13%	3.0E-02	Scyllo
0.959	7%	0.182	Tau
0.994	10%	0.189	-CrCH2
0.771	2%	0.146	GPC+PCh
7.080	2%	1.345	NAA+NAAG
5.262	1%	1.000	Cr+PCr
5.812	2%	1.105	Glu+Gln
1.055	40%	0.201	Lip13a
0.000	999%	0.000	Lip13b
0.363	54%	6.9E-02	Lip09
3.017	12%	0.573	MM09
0.000	999%	0.000	Lip20
3.830	15%	0.728	MM20
1.315	19%	0.250	MM12
5.252	13%	0.998	MM14
7.986	*8%*	*1.518*	*+MM17*
1.055	40%	0.201	Lip13a+Lip13b
7.623	11%	1.449	MM14+Lip13a+L
3.381	8%	0.642	MM09+Lip09
3.830	15%	0.728	MM20+Lip20

DIAGNOSTICS

1 info   STARTV 24

2 info's   RFALSI 11

Doing Water-Scaling

MISCELLANEOUS OUTPUT

FWHM = 0.040 ppm   S/N = 44

Data shift = 0.020 ppm

Ph: 3 deg   -23.9 deg/ppm

MIGRAINE WITH AURA-42yo-Male-Pre-checkerboard

LCModel (Version 6.3-0B) Copyright: S.W. Provencher. *Ref.: Magn. Reson. Med. 30:672-679 (1993).* *30-July-2018 15:59*

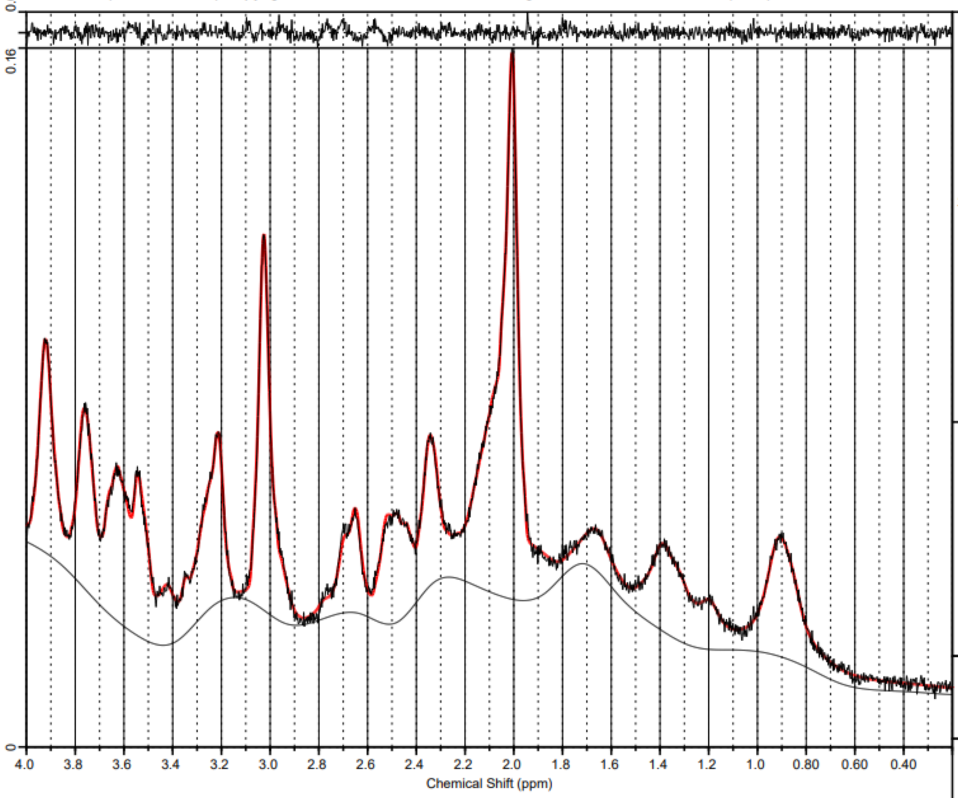

Conc.	%SD	/Cr+PCr	Metabolite
0.143	42%	2.8E-02	Ala
0.413	38%	7.9E-02	Asp
1.868	9%	0.359	Cr
3.338	6%	0.641	PCr
0.554	11%	0.106	GABA
1.122	15%	0.215	Glc
1.323	5%	0.254	Gln
4.588	2%	0.881	Glu
0.707	9%	0.136	GPC
3.44E-03	999%	6.6E-04	PCh
1.261	4%	0.242	GSH
4.294	3%	0.825	Ins
0.312	28%	6.0E-02	Lac
7.078	2%	1.360	NAA
0.422	22%	8.1E-02	NAAG
0.314	8%	6.0E-02	Scyllo
1.043	7%	0.200	Tau
0.793	13%	0.152	-CrCH2
0.711	3%	0.137	GPC+PCh
7.500	2%	1.441	NAA+NAAG
5.206	1%	1.000	Cr+PCr
5.911	2%	1.135	Glu+Gln
1.524	38%	0.293	Lip13a
0.000	999%	0.000	Lip13b
0.626	34%	0.120	Lip09
5.193	11%	0.998	MM09
0.608	23%	0.117	Lip20
5.665	14%	1.088	MM20
1.489	25%	0.286	MM12
4.728	17%	0.908	MM14
2.569	23%	0.493	MM17
1.524	38%	0.293	Lip13a+Lip13b
7.741	14%	1.487	MM14+Lip13a+L
5.820	8%	1.118	MM09+Lip09
6.273	13%	1.205	MM20+Lip20

DIAGNOSTICS

1 info STARTV 24
4 info's RFALSI 11
Doing Water-Scaling

MISCELLANEOUS OUTPUT

FWHM = 0.040 ppm S/N = 52
Data shift = 0.007 ppm
Ph: -7 deg -21.9 deg/ppm

MIGRAINE WITH AURA-42yo-Male-Post-checkerboard

LCModel (Version 6.3-0B) Copyright: S.W. Provencher. *Ref.: Magn. Reson. Med. 30:672-679 (1993).* *30-July-2018 15:59*

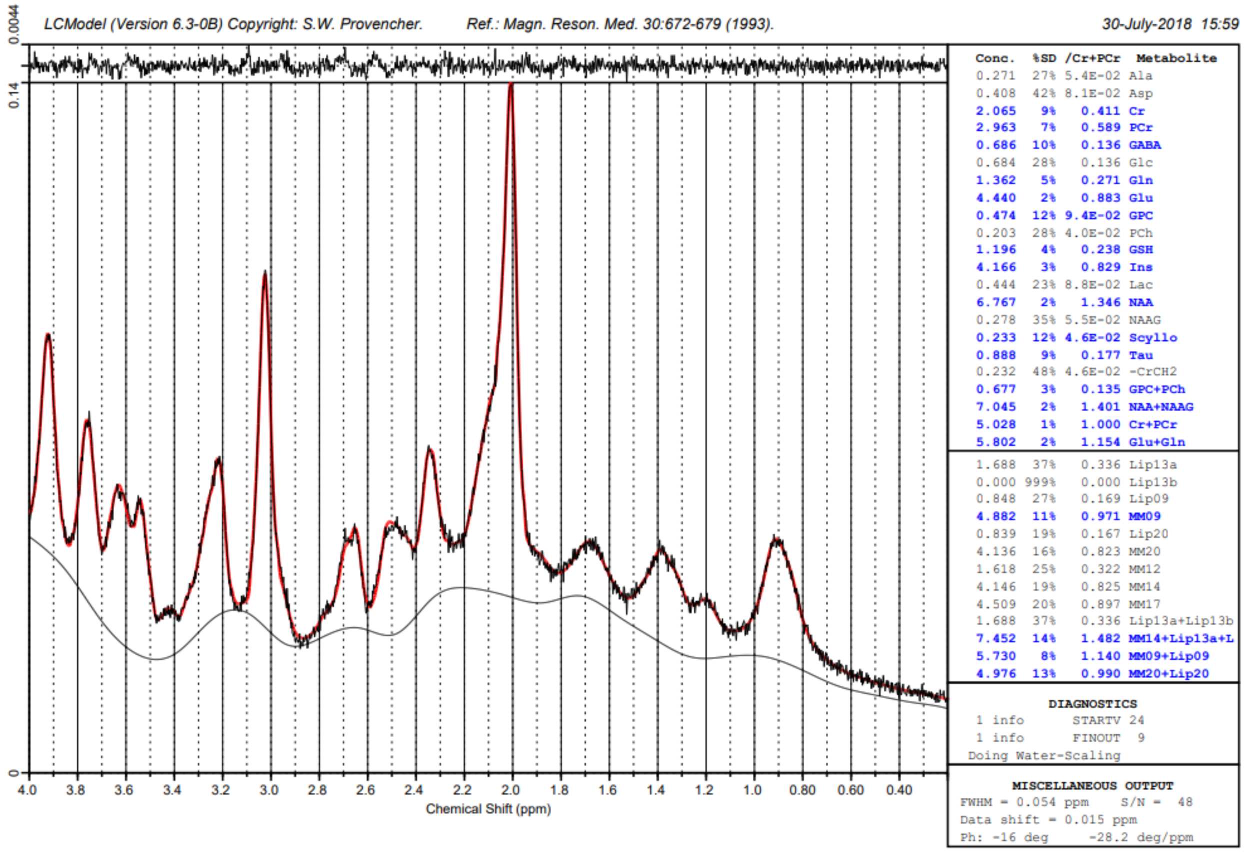

Conc.	%SD	/Cr+PCr	Metabolite
0.271	27%	5.4E-02	Ala
0.408	42%	8.1E-02	Asp
2.065	9%	0.411	Cr
2.963	7%	0.589	PCr
0.686	10%	0.136	GABA
0.684	28%	0.136	Glc
1.362	5%	0.271	Gln
4.440	2%	0.883	Glu
0.474	12%	9.4E-02	GPC
0.203	28%	4.0E-02	PCh
1.196	4%	0.238	GSH
4.166	3%	0.829	Ins
0.444	23%	8.8E-02	Lac
6.767	2%	1.346	NAA
0.278	35%	5.5E-02	NAAG
0.233	12%	4.6E-02	Scyllo
0.888	9%	0.177	Tau
0.232	48%	4.6E-02	-CrCH2
0.677	3%	0.135	GPC+PCh
7.045	2%	1.401	NAA+NAAG
5.028	1%	1.000	Cr+PCr
5.802	2%	1.154	Glu+Gln
1.688	37%	0.336	Lip13a
0.000	999%	0.000	Lip13b
0.848	27%	0.169	Lip09
4.882	11%	0.971	MM09
0.839	19%	0.167	Lip20
4.136	16%	0.823	MM20
1.618	25%	0.322	MM12
4.146	19%	0.825	MM14
4.509	20%	0.897	MM17
1.688	37%	0.336	Lip13a+Lip13b
7.452	14%	1.482	MM14+Lip13a+L
5.730	8%	1.140	MM09+Lip09
4.976	13%	0.990	MM20+Lip20

DIAGNOSTICS

1 info STARTV 24
1 info FINOUT 9
Doing Water-Scaling

MISCELLANEOUS OUTPUT

FWHM = 0.054 ppm S/N = 48
Data shift = 0.015 ppm
Ph: -16 deg -28.2 deg/ppm

MIGRAINE WITH AURA-26yo-Female-Pre-checkerboard

LCModel (Version 6.3-0B) Copyright: S.W. Provencher. *Ref.: Magn. Reson. Med. 30:672-679 (1993).* *30-July-2018 16:00*

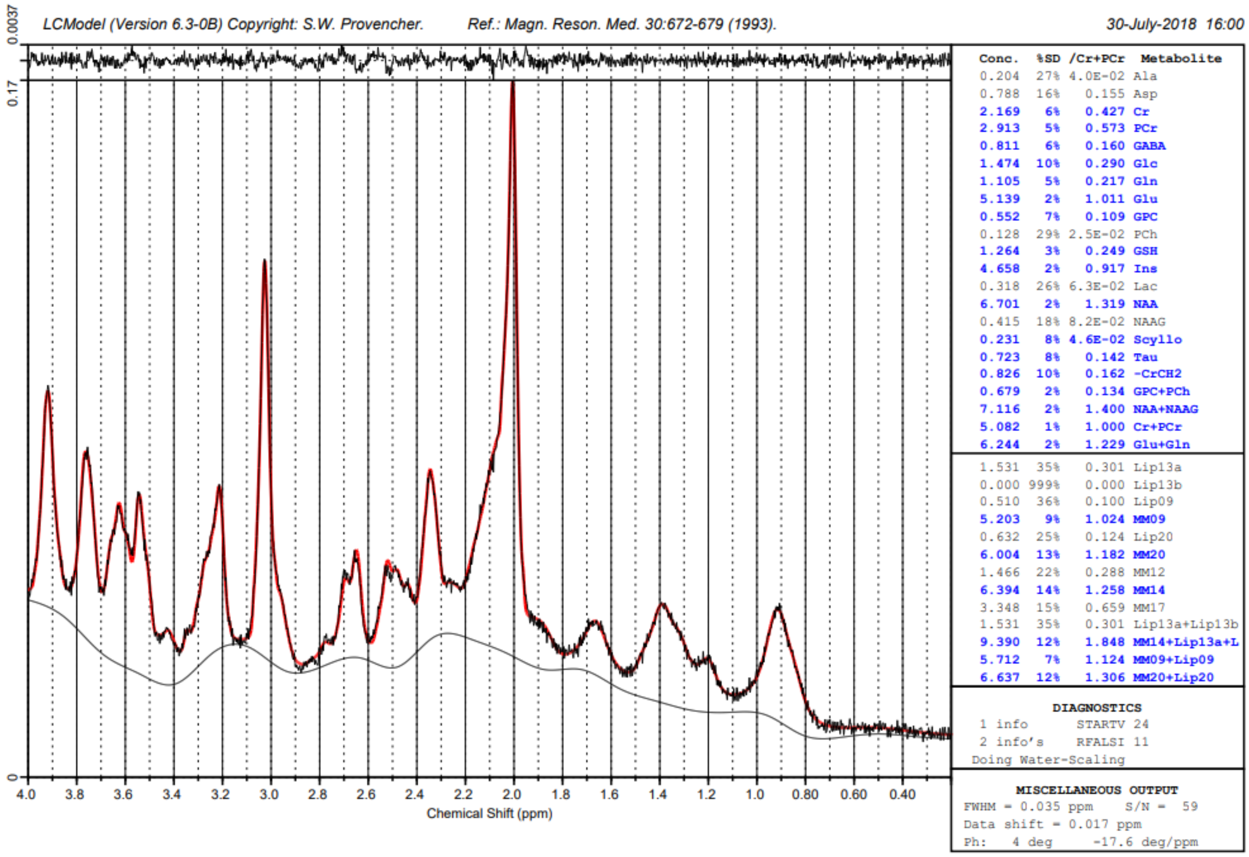

Conc.	%SD	/Cr+PCr	Metabolite
0.204	27%	4.0E-02	Ala
0.788	16%	0.155	Asp
2.169	6%	0.427	Cr
2.913	5%	0.573	PCr
0.811	6%	0.160	GABA
1.474	10%	0.290	Glc
1.105	5%	0.217	Gln
5.139	2%	1.011	Glu
0.552	7%	0.109	GPC
0.128	29%	2.5E-02	PCh
1.264	3%	0.249	GSH
4.658	2%	0.917	Ins
0.318	26%	6.3E-02	Lac
6.701	2%	1.319	NAA
0.415	18%	8.2E-02	NAAG
0.231	8%	4.6E-02	Scyllo
0.723	8%	0.142	Tau
0.826	10%	0.162	-CrCH2
0.679	2%	0.134	GPC+PCh
7.116	2%	1.400	NAA+NAAG
5.082	1%	1.000	Cr+PCr
6.244	2%	1.229	Glu+Gln
1.531	35%	0.301	Lip13a
0.000	999%	0.000	Lip13b
0.510	36%	0.100	Lip09
5.203	9%	1.024	MM09
0.632	25%	0.124	Lip20
6.004	13%	1.182	MM20
1.466	22%	0.288	MM12
6.394	14%	1.258	MM14
3.348	15%	0.659	MM17
1.531	35%	0.301	Lip13a+Lip13b
9.390	12%	1.848	MM14+Lip13a+L
5.712	7%	1.124	MM09+Lip09
6.637	12%	1.306	MM20+Lip20

DIAGNOSTICS

1 info STARTV 24
2 info's RFALSI 11
Doing Water-Scaling

MISCELLANEOUS OUTPUT

FWHM = 0.035 ppm S/N = 59
Data shift = 0.017 ppm
Ph: 4 deg -17.6 deg/ppm

MIGRAINE WITH AURA-26yo-Female-Post-checkerboard

LCModel (Version 6.3-0B) Copyright: S.W. Provencher. *Ref.: Magn. Reson. Med. 30:672-679 (1993).* *30-July-2018 16:00*

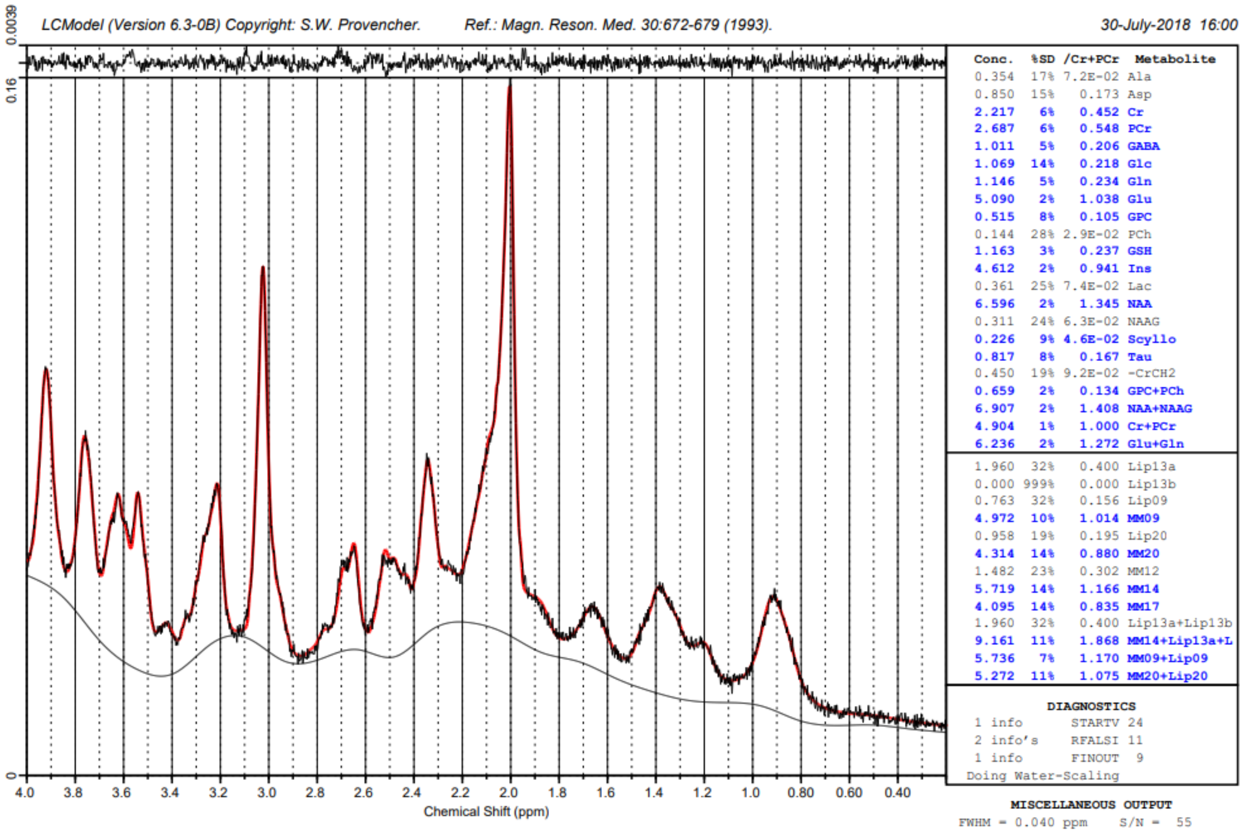

Conc.	%SD	/Cr+PCr	Metabolite
0.354	17%	7.2E-02	Ala
0.850	15%	0.173	Asp
2.217	6%	0.452	Cr
2.687	6%	0.548	PCr
1.011	5%	0.206	GABA
1.069	14%	0.218	Glc
1.146	5%	0.234	Gln
5.090	2%	1.038	Glu
0.515	8%	0.105	GPC
0.144	28%	2.9E-02	PCh
1.163	3%	0.237	GSH
4.612	2%	0.941	Ins
0.361	25%	7.4E-02	Lac
6.596	2%	1.345	NAA
0.311	24%	6.3E-02	NAAG
0.226	9%	4.6E-02	Scyllo
0.817	8%	0.167	Tau
0.450	19%	9.2E-02	-CrCH2
0.659	2%	0.134	GPC+PCh
6.907	2%	1.408	NAA+NAAG
4.904	1%	1.000	Cr+PCr
6.236	2%	1.272	Glu+Gln
1.960	32%	0.400	Lip13a
0.000	999%	0.000	Lip13b
0.763	32%	0.156	Lip09
4.972	10%	1.014	MM09
0.958	19%	0.195	Lip20
4.314	14%	0.880	MM20
1.482	23%	0.302	MM12
5.719	14%	1.166	MM14
4.095	14%	0.835	MM17
1.960	32%	0.400	Lip13a+Lip13b
9.161	11%	1.868	MM14+Lip13a+L
5.736	7%	1.170	MM09+Lip09
5.272	11%	1.075	MM20+Lip20

DIAGNOSTICS

1 info	STARTV 24
2 info's	RFALSI 11
1 info	FINOUT 9

Doing Water-Scaling

MISCELLANEOUS OUTPUT

FWHM = 0.040 ppm S/N = 55

Data shift = 0.015 ppm

CONTROL-44yo-Male-Pre-Checkerboard

LCModel (Version 6.3-0B) Copyright: S.W. Provencher. *Ref.: Magn. Reson. Med. 30:672-679 (1993).* *30-July-2018 16:00*

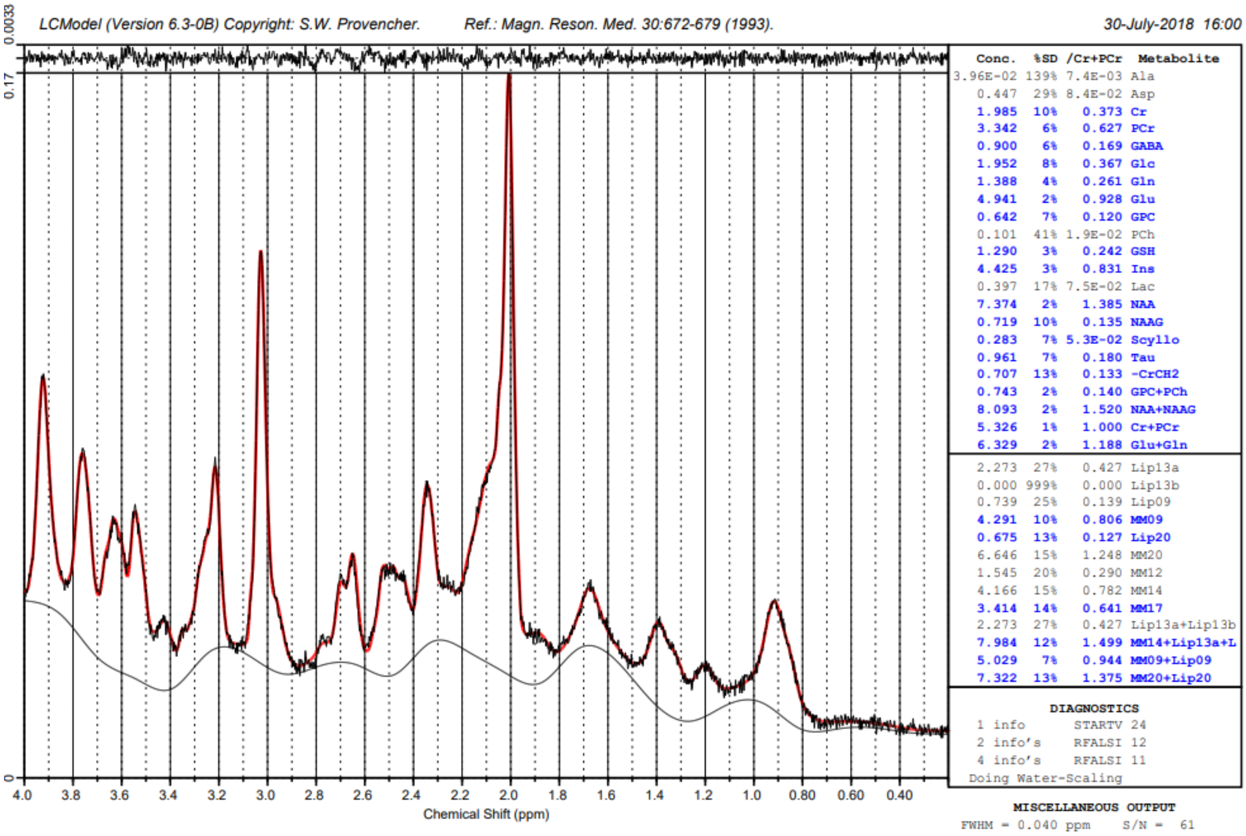

Conc.	%SD	/Cr+PCr	Metabolite
3.96E-02	139%	7.4E-03	Ala
0.447	29%	8.4E-02	Asp
1.985	10%	0.373	Cr
3.342	6%	0.627	PCr
0.900	6%	0.169	GABA
1.952	8%	0.367	Glc
1.388	4%	0.261	Gln
4.941	2%	0.928	Glu
0.642	7%	0.120	GPC
0.101	41%	1.9E-02	PCh
1.290	3%	0.242	GSH
4.425	3%	0.831	Ins
0.397	17%	7.5E-02	Lac
7.374	2%	1.385	NAA
0.719	10%	0.135	NAAG
0.283	7%	5.3E-02	Scyllo
0.961	7%	0.180	Tau
0.707	13%	0.133	-CrCH2
0.743	2%	0.140	GPC+PCh
8.093	2%	1.520	NAA+NAAG
5.326	1%	1.000	Cr+PCr
6.329	2%	1.188	Glu+Gln
2.273	27%	0.427	Lip13a
0.000	999%	0.000	Lip13b
0.739	25%	0.139	Lip09
4.291	10%	0.806	MM09
0.675	13%	0.127	Lip20
6.646	15%	1.248	MM20
1.545	20%	0.290	MM12
4.166	15%	0.782	MM14
3.414	14%	0.641	MM17
2.273	27%	0.427	Lip13a+Lip13b
7.984	12%	1.499	MM14+Lip13a+L
5.029	7%	0.944	MM09+Lip09
7.322	13%	1.375	MM20+Lip20

DIAGNOSTICS

1 info	STARTV 24
2 info's	RFALSI 12
4 info's	RFALSI 11

Doing Water-Scaling

MISCELLANEOUS OUTPUT

FWHM = 0.040 ppm S/N = 61

Data shift = 0.030 ppm

CONTROL-44yo-Male-Post-Checkerboard

LCModel (Version 6.3-0B) Copyright: S.W. Provencher. Ref.: Magn. Reson. Med. 30:672-679 (1993). 30-July-2018 16:00

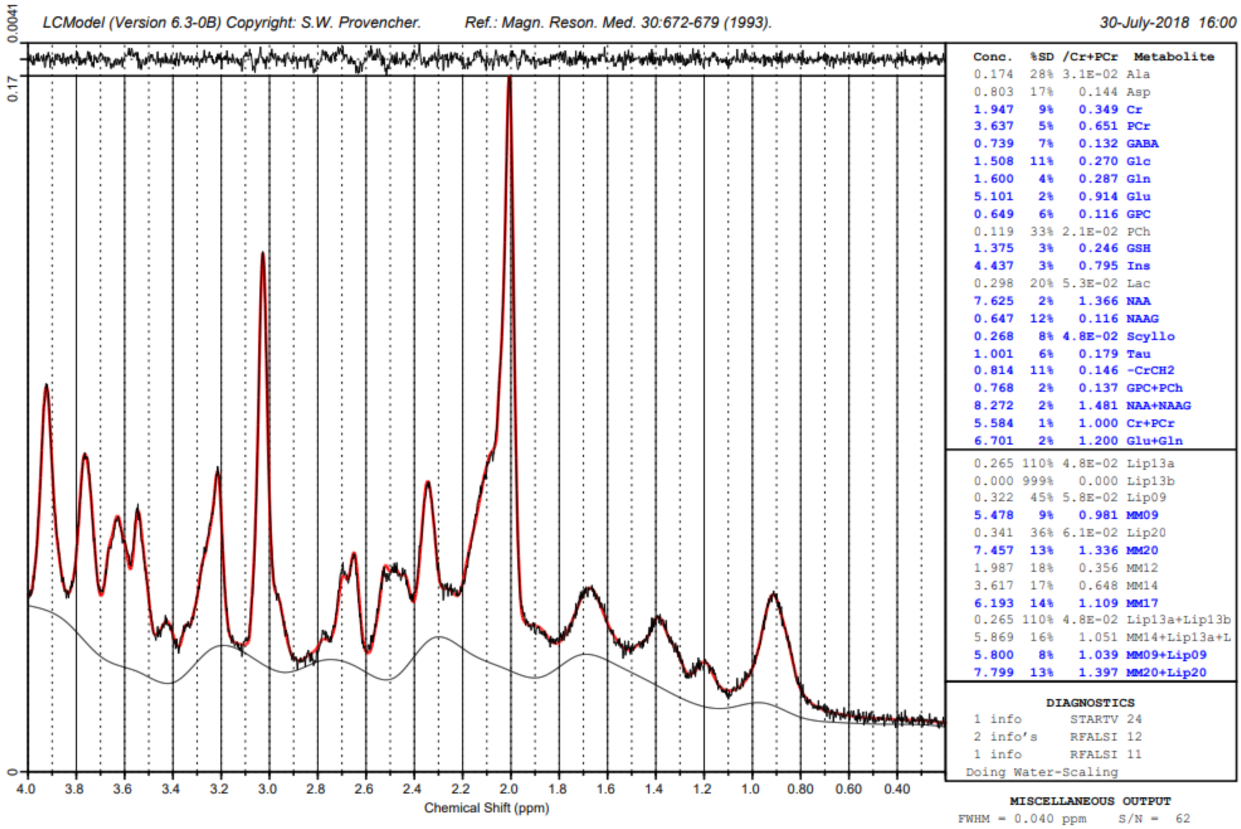

Conc.	%SD	/Cr+PCr	Metabolite
0.174	28%	3.1E-02	Ala
0.803	17%	0.144	Asp
1.947	9%	0.349	Cr
3.637	5%	0.651	PCr
0.739	7%	0.132	GABA
1.508	11%	0.270	Glc
1.600	4%	0.287	Gln
5.101	2%	0.914	Glu
0.649	6%	0.116	GPC
0.119	33%	2.1E-02	PCh
1.375	3%	0.246	GSH
4.437	3%	0.795	Ins
0.298	20%	5.3E-02	Lac
7.625	2%	1.366	NAA
0.647	12%	0.116	NAAG
0.268	8%	4.8E-02	Scyllo
1.001	6%	0.179	Tau
0.814	11%	0.146	-CrCH2
0.768	2%	0.137	GPC+PCh
8.272	2%	1.481	NAA+NAAG
5.584	1%	1.000	Cr+PCr
6.701	2%	1.200	Glu+Gln
0.265	110%	4.8E-02	Lip13a
0.000	999%	0.000	Lip13b
0.322	45%	5.8E-02	Lip09
5.478	9%	0.981	MM09
0.341	36%	6.1E-02	Lip20
7.457	13%	1.336	MM20
1.987	18%	0.356	MM12
3.617	17%	0.648	MM14
6.193	14%	1.109	MM17
0.265	110%	4.8E-02	Lip13a+Lip13b
5.869	16%	1.051	MM14+Lip13a+L
5.800	8%	1.039	MM09+Lip09
7.799	13%	1.397	MM20+Lip20

DIAGNOSTICS

1 info STARTV 24
2 info's RFALSI 12
1 info RFALSI 11
Doing Water-Scaling

MISCELLANEOUS OUTPUT

FWHM = 0.040 ppm S/N = 62
Data shift = 0.030 ppm

MIGRAINE WITH AURA-27yo-Female-Pre-checkerboard

LCModel (Version 6.3-0B) Copyright: S.W. Provencher. Ref.: Magn. Reson. Med. 30:672-679 (1993). 30-July-2018 16:01

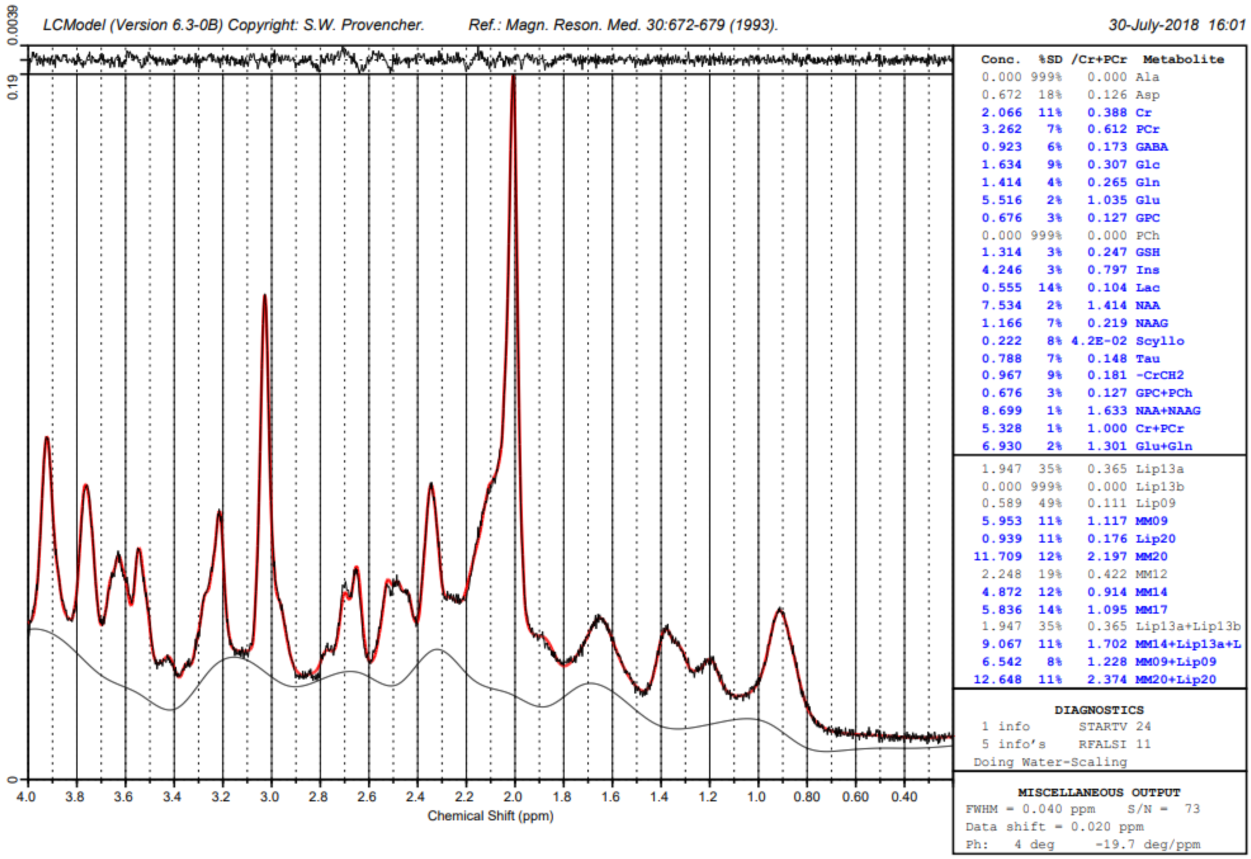

Conc.	%SD	/Cr+PCr	Metabolite
0.000	999%	0.000	Ala
0.672	18%	0.126	Asp
2.066	11%	0.388	Cr
3.262	7%	0.612	PCr
0.923	6%	0.173	GABA
1.634	9%	0.307	Glc
1.414	4%	0.265	Gln
5.516	2%	1.035	Glu
0.676	3%	0.127	GPC
0.000	999%	0.000	PCh
1.314	3%	0.247	GSH
4.246	3%	0.797	Ins
0.555	14%	0.104	Lac
7.534	2%	1.414	NAA
1.166	7%	0.219	NAAG
0.222	8%	4.2E-02	Scyllo
0.788	7%	0.148	Tau
0.967	9%	0.181	-CrCH2
0.676	3%	0.127	GPC+PCh
8.699	1%	1.633	NAA+NAAG
5.328	1%	1.000	Cr+PCr
6.930	2%	1.301	Glu+Gln
1.947	35%	0.365	Lip13a
0.000	999%	0.000	Lip13b
0.589	49%	0.111	Lip09
5.953	11%	1.117	MM09
0.939	11%	0.176	Lip20
11.709	12%	2.197	MM20
2.248	19%	0.422	MM12
4.872	12%	0.914	MM14
5.836	14%	1.095	MM17
1.947	35%	0.365	Lip13a+Lip13b
9.067	11%	1.702	MM14+Lip13a+L
6.542	8%	1.228	MM09+Lip09
12.648	11%	2.374	MM20+Lip20

DIAGNOSTICS

1 info STARTV 24
5 info's RFALSI 11
Doing Water-Scaling

MISCELLANEOUS OUTPUT

FWHM = 0.040 ppm S/N = 73
Data shift = 0.020 ppm
Ph: 4 deg -19.7 deg/ppm

MIGRAINE WITH AURA-27yo-Female-Post-checkerboard

LCModel (Version 6.3-0B) Copyright: S.W. Provencher. *Ref.: Magn. Reson. Med. 30:672-679 (1993).* *30-July-2018 16:00*

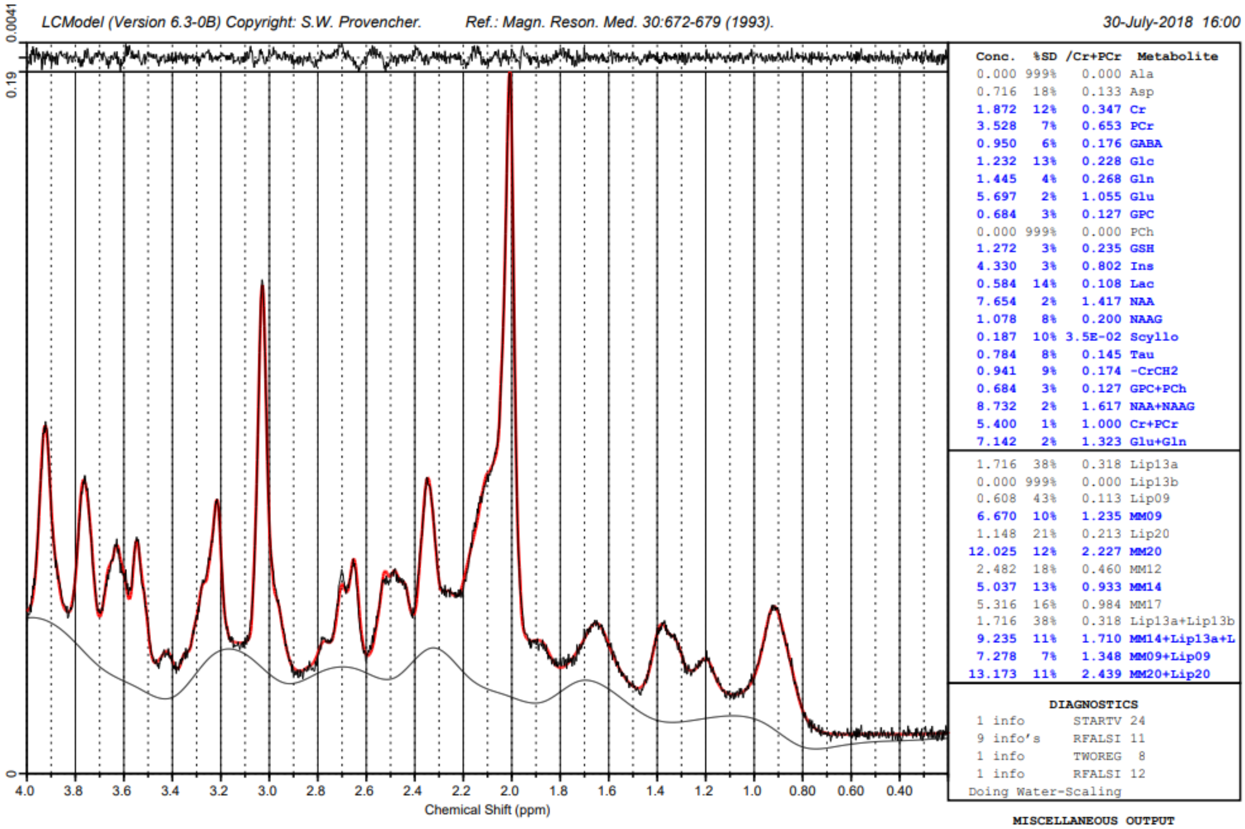

Conc.	%SD	/Cr+PCr	Metabolite
0.000	999%	0.000	Ala
0.716	18%	0.133	Asp
1.872	12%	0.347	Cr
3.528	7%	0.653	PCr
0.950	6%	0.176	GABA
1.232	13%	0.228	Glc
1.445	4%	0.268	Gln
5.697	2%	1.055	Glu
0.684	3%	0.127	GPC
0.000	999%	0.000	PCh
1.272	3%	0.235	GSH
4.330	3%	0.802	Ins
0.584	14%	0.108	Lac
7.654	2%	1.417	NAA
1.078	8%	0.200	NAAG
0.187	10%	3.5E-02	Scyllo
0.784	8%	0.145	Tau
0.941	9%	0.174	-CrCH2
0.684	3%	0.127	GPC+PCh
8.732	2%	1.617	NAA+NAAG
5.400	1%	1.000	Cr+PCr
7.142	2%	1.323	Glu+Gln
1.716	38%	0.318	Lip13a
0.000	999%	0.000	Lip13b
0.608	43%	0.113	Lip09
6.670	10%	1.235	MM09
1.148	21%	0.213	Lip20
12.025	12%	2.227	MM20
2.482	18%	0.460	MM12
5.037	13%	0.933	MM14
5.316	16%	0.984	MM17
1.716	38%	0.318	Lip13a+Lip13b
9.235	11%	1.710	MM14+Lip13a+L
7.278	7%	1.348	MM09+Lip09
13.173	11%	2.439	MM20+Lip20

DIAGNOSTICS

1 info	STARTV 24
9 info's	RFALSI 11
1 info	TWOREG 8
1 info	RFALSI 12

Doing Water-Scaling

MISCELLANEOUS OUTPUT

FWHM = 0.040 ppm S/N = 68

CONTROL-24yo-Male-Pre-checkerboard

LCModel (Version 6.3-0B) Copyright: S.W. Provencher. *Ref.: Magn. Reson. Med. 30:672-679 (1993).* *30-July-2018 16:01*

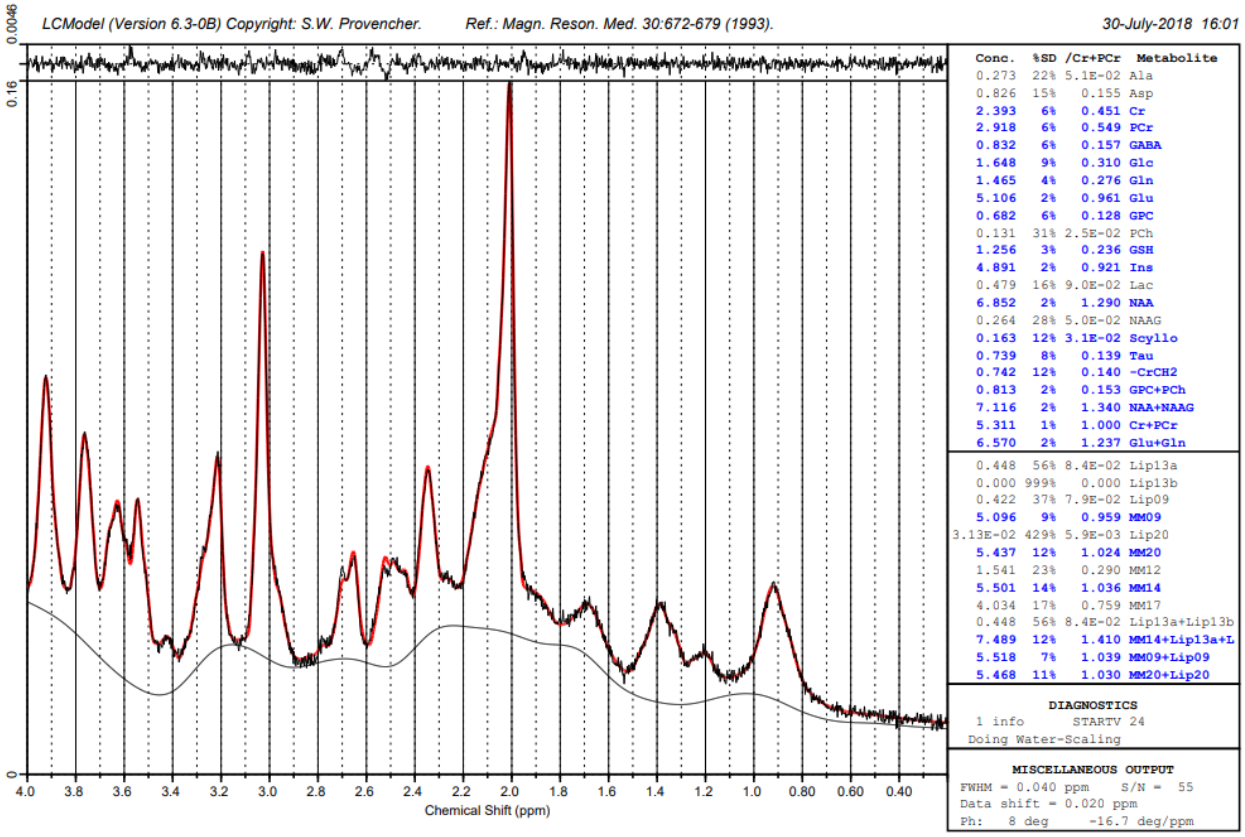

Conc.	%SD	/Cr+PCr	Metabolite
0.273	22%	5.1E-02	Ala
0.826	15%	0.155	Asp
2.393	6%	0.451	Cr
2.918	6%	0.549	PCr
0.832	6%	0.157	GABA
1.648	9%	0.310	Glc
1.465	4%	0.276	Gln
5.106	2%	0.961	Glu
0.682	6%	0.128	GPC
0.131	31%	2.5E-02	PCh
1.256	3%	0.236	GSH
4.891	2%	0.921	Ins
0.479	16%	9.0E-02	Lac
6.852	2%	1.290	NAA
0.264	28%	5.0E-02	NAAG
0.163	12%	3.1E-02	Scyllo
0.739	8%	0.139	Tau
0.742	12%	0.140	-CrCH2
0.813	2%	0.153	GPC+PCh
7.116	2%	1.340	NAA+NAAG
5.311	1%	1.000	Cr+PCr
6.570	2%	1.237	Glu+Gln
0.448	56%	8.4E-02	Lip13a
0.000	999%	0.000	Lip13b
0.422	37%	7.9E-02	Lip09
5.096	9%	0.959	MM09
3.13E-02	429%	5.9E-03	Lip20
5.437	12%	1.024	MM20
1.541	23%	0.290	MM12
5.501	14%	1.036	MM14
4.034	17%	0.759	MM17
0.448	56%	8.4E-02	Lip13a+Lip13b
7.489	12%	1.410	MM14+Lip13a+L
5.518	7%	1.039	MM09+Lip09
5.468	11%	1.030	MM20+Lip20

DIAGNOSTICS

1 info STARTV 24

Doing Water-Scaling

MISCELLANEOUS OUTPUT

FWHM = 0.040 ppm S/N = 55

Data shift = 0.020 ppm

Ph: 8 deg -16.7 deg/ppm

CONTROL-24yo-Male-Post-checkerboard

LCModel (Version 6.3-0B) Copyright: S.W. Provencher. Ref.: Magn. Reson. Med. 30:672-679 (1993). 30-July-2018 16:01

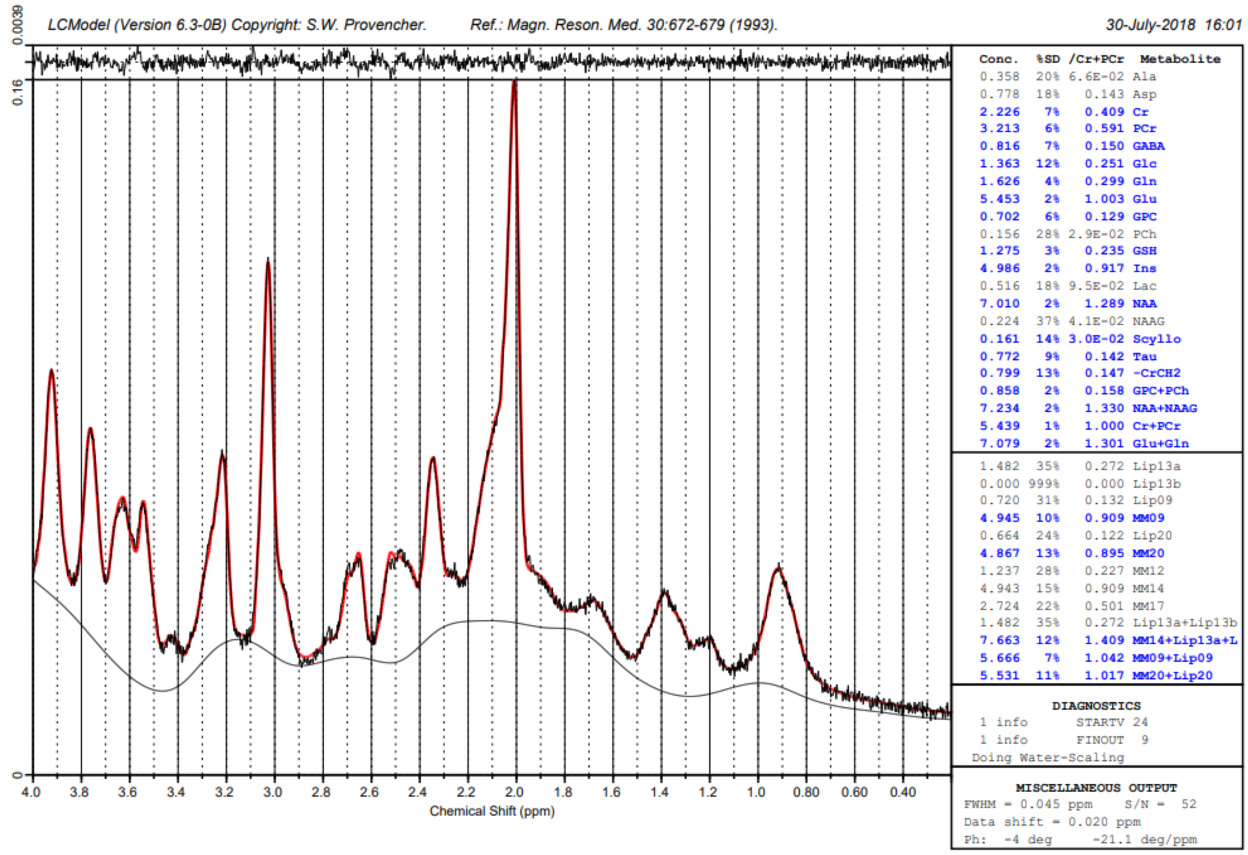

Conc.	%SD	/Cr+PCr	Metabolite
0.358	20%	6.6E-02	Ala
0.778	18%	0.143	Asp
2.226	7%	0.409	Cr
3.213	6%	0.591	PCr
0.816	7%	0.150	GABA
1.363	12%	0.251	Glc
1.626	4%	0.299	Gln
5.453	2%	1.003	Glu
0.702	6%	0.129	GPC
0.156	28%	2.9E-02	PCh
1.275	3%	0.235	GSH
4.986	2%	0.917	Ins
0.516	18%	9.5E-02	Lac
7.010	2%	1.289	NAA
0.224	37%	4.1E-02	NAAG
0.161	14%	3.0E-02	Scyllo
0.772	9%	0.142	Tau
0.799	13%	0.147	-CrCH2
0.858	2%	0.158	GPC+PCh
7.234	2%	1.330	NAA+NAAG
5.439	1%	1.000	Cr+PCr
7.079	2%	1.301	Glu+Gln
1.482	35%	0.272	Lip13a
0.000	999%	0.000	Lip13b
0.720	31%	0.132	Lip09
4.945	10%	0.909	MM09
0.664	24%	0.122	Lip20
4.867	13%	0.895	MM20
1.237	28%	0.227	MM12
4.943	15%	0.909	MM14
2.724	22%	0.501	MM17
1.482	35%	0.272	Lip13a+Lip13b
7.663	12%	1.409	MM14+Lip13a+L
5.666	7%	1.042	MM09+Lip09
5.531	11%	1.017	MM20+Lip20

DIAGNOSTICS

1 info STARTV 24
1 info FINOUT 9
Doing Water-Scaling

MISCELLANEOUS OUTPUT

FWHM = 0.045 ppm S/N = 52
Data shift = 0.020 ppm
Ph: -4 deg -21.1 deg/ppm

CONTROL-29yo-Male-Pre-checkerboard

LCModel (Version 6.3-0B) Copyright: S.W. Provencher. *Ref.: Magn. Reson. Med. 30:672-679 (1993).* *31-July-2018 13:51*

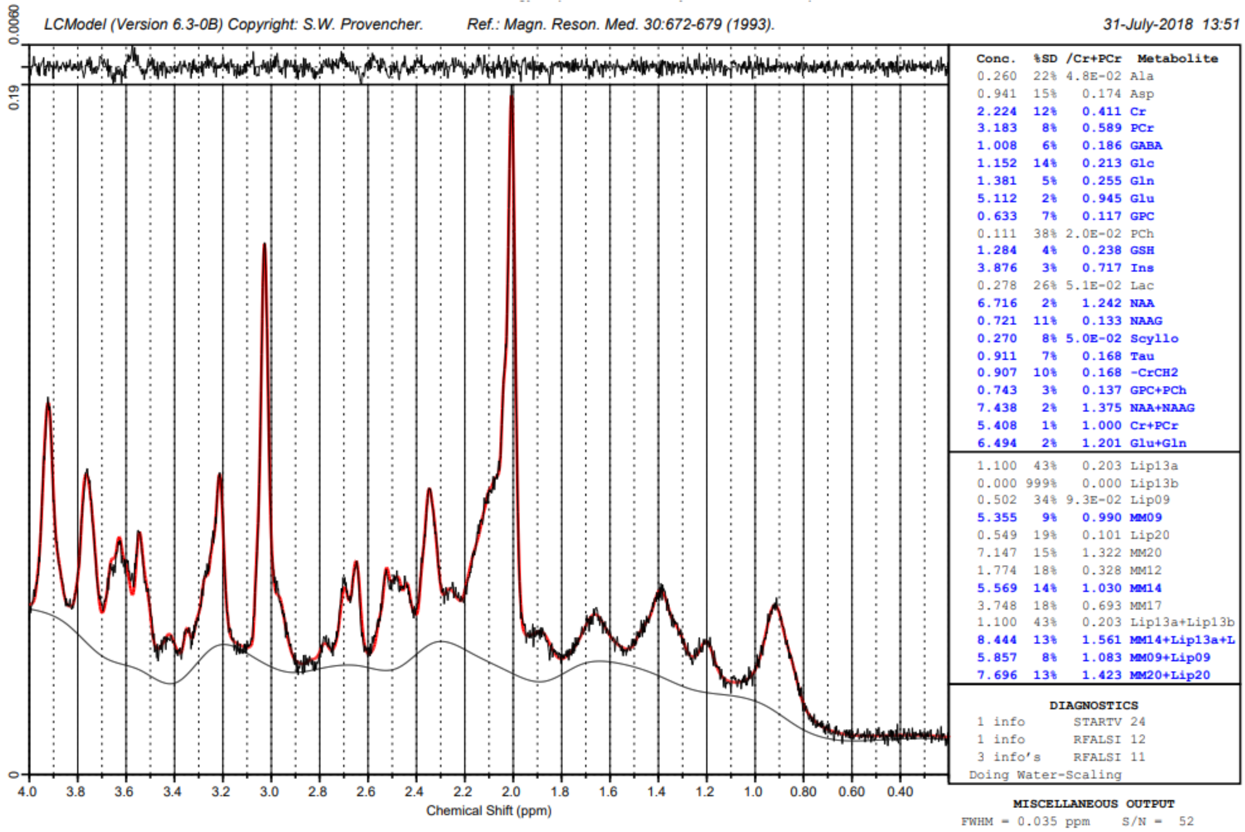

Conc.	%SD	/Cr+PCr	Metabolite
0.260	22%	4.8E-02	Ala
0.941	15%	0.174	Asp
2.224	12%	0.411	Cr
3.183	8%	0.589	PCr
1.008	6%	0.186	GABA
1.152	14%	0.213	Glc
1.381	5%	0.255	Gln
5.112	2%	0.945	Glu
0.633	7%	0.117	GPC
0.111	38%	2.0E-02	PCh
1.284	4%	0.238	GSH
3.876	3%	0.717	Ins
0.278	26%	5.1E-02	Lac
6.716	2%	1.242	NAA
0.721	11%	0.133	NAAG
0.270	8%	5.0E-02	Scyllo
0.911	7%	0.168	Tau
0.907	10%	0.168	-CrCH2
0.743	3%	0.137	GPC+PCh
7.438	2%	1.375	NAA+NAAG
5.408	1%	1.000	Cr+PCr
6.494	2%	1.201	Glu+Gln
1.100	43%	0.203	Lip13a
0.000	999%	0.000	Lip13b
0.502	34%	9.3E-02	Lip09
5.355	9%	0.990	MM09
0.549	19%	0.101	Lip20
7.147	15%	1.322	MM20
1.774	18%	0.328	MM12
5.569	14%	1.030	MM14
3.748	18%	0.693	MM17
1.100	43%	0.203	Lip13a+Lip13b
8.444	13%	1.561	MM14+Lip13a+L
5.857	8%	1.083	MM09+Lip09
7.696	13%	1.423	MM20+Lip20

DIAGNOSTICS

1 info STARTV 24

1 info RFALSI 12

3 info's RFALSI 11

Doing Water-Scaling

MISCELLANEOUS OUTPUT

FWHM = 0.035 ppm S/N = 52

Data shift = 0.020 ppm

CONTROL-29yo-Male-Post-checkerboard

LCModel (Version 6.3-0B) Copyright: S.W. Provencher. *Ref.: Magn. Reson. Med. 30:672-679 (1993).* *31-July-2018 13:51*

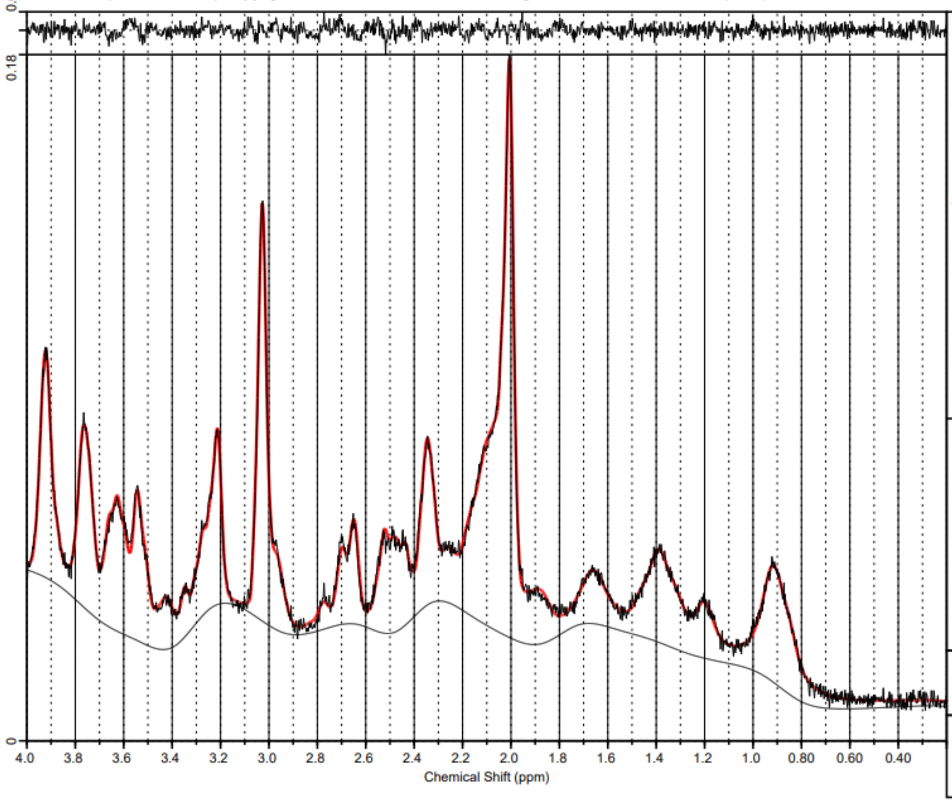

Conc.	%SD	/Cr+PCr	Metabolite
0.225	28%	4.2E-02	Ala
0.826	18%	0.154	Asp
2.293	9%	0.427	Cr
3.074	7%	0.573	PCr
0.957	6%	0.178	GABA
1.415	12%	0.264	Glc
1.468	4%	0.274	Gln
5.303	2%	0.988	Glu
0.698	7%	0.130	GPC
5.69E-02	85%	1.1E-02	PCh
1.299	4%	0.242	GSH
4.079	3%	0.760	Ins
0.230	32%	4.3E-02	Lac
6.781	2%	1.264	NAA
0.872	9%	0.162	NAAG
0.297	8%	5.5E-02	Scyllo
0.814	8%	0.152	Tau
0.675	15%	0.126	-CrCH2
0.755	3%	0.141	GPC+PCh
7.653	2%	1.426	NAA+NAAG
5.366	1%	1.000	Cr+PCr
6.771	2%	1.262	Glu+Gln
0.802	54%	0.150	Lip13a
0.000	999%	0.000	Lip13b
0.501	38%	9.3E-02	Lip09
5.874	10%	1.095	MM09
0.759	14%	0.141	Lip20
6.868	15%	1.280	MM20
2.091	18%	0.390	MM12
6.314	15%	1.177	MM14
3.847	17%	0.717	MM17
0.802	54%	0.150	Lip13a+Lip13b
9.208	13%	1.716	MM14+Lip13a+L
6.375	8%	1.188	MM09+Lip09
7.626	14%	1.421	MM20+Lip20

DIAGNOSTICS

1 info STARTV 24
Doing Water-Scaling

MISCELLANEOUS OUTPUT

FWHM = 0.035 ppm S/N = 49
Data shift = 0.030 ppm
Ph: 4 deg -20.2 deg/ppm

CONTROL-46yo-Male-Pre-checkerboard

LCModel (Version 6.3-0B) Copyright: S.W. Provencher. *Ref.: Magn. Reson. Med. 30:672-679 (1993).* *31-July-2018 13:51*

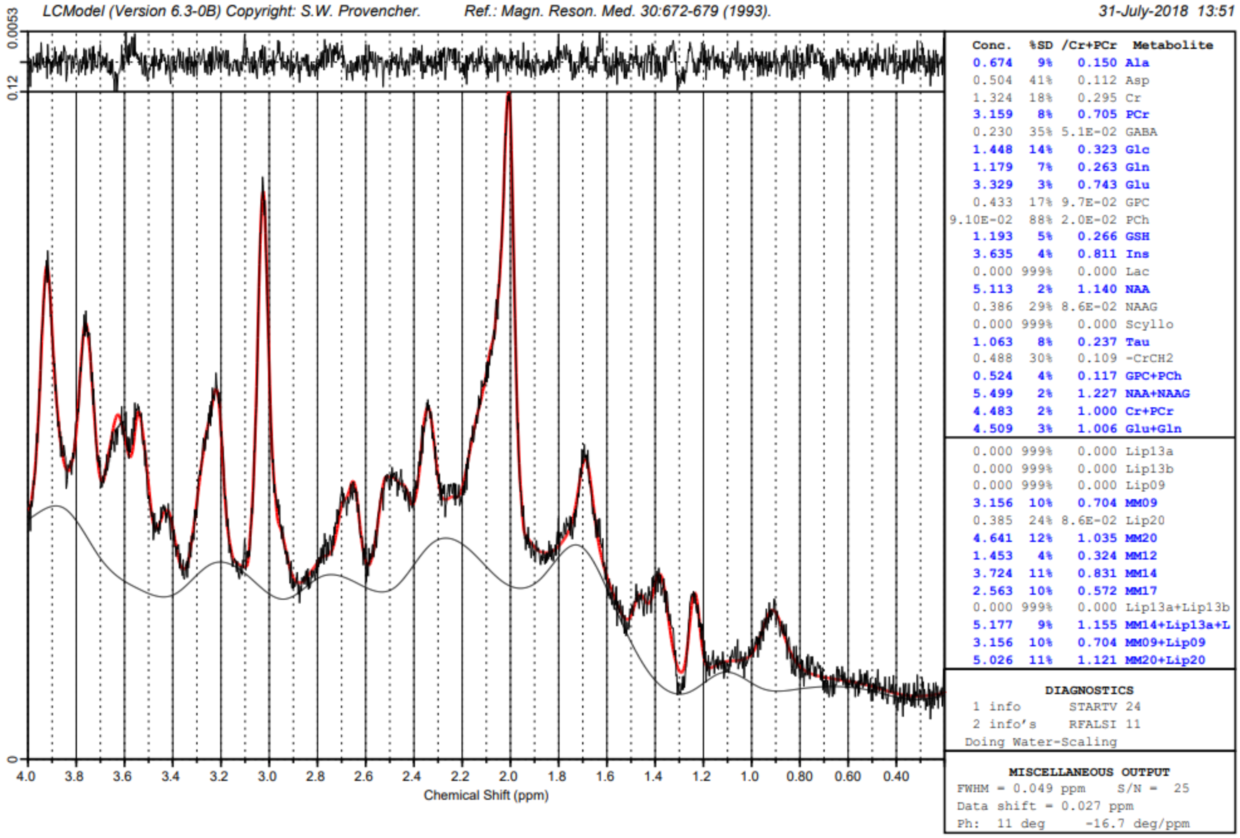

Conc.	%SD	/Cr+PCr	Metabolite
0.674	9%	0.150	Ala
0.504	41%	0.112	Asp
1.324	18%	0.295	Cr
3.159	8%	0.705	PCr
0.230	35%	5.1E-02	GABA
1.448	14%	0.323	Glc
1.179	7%	0.263	Gln
3.329	3%	0.743	Glu
0.433	17%	9.7E-02	GPC
9.10E-02	88%	2.0E-02	PCh
1.193	5%	0.266	GSH
3.635	4%	0.811	Ins
0.000	999%	0.000	Lac
5.113	2%	1.140	NAA
0.386	29%	8.6E-02	NAAG
0.000	999%	0.000	Scyllo
1.063	8%	0.237	Tau
0.488	30%	0.109	-CrCH2
0.524	4%	0.117	GPC+PCh
5.499	2%	1.227	NAA+NAAG
4.483	2%	1.000	Cr+PCr
4.509	3%	1.006	Glu+Gln
0.000	999%	0.000	Lip13a
0.000	999%	0.000	Lip13b
0.000	999%	0.000	Lip09
3.156	10%	0.704	MM09
0.385	24%	8.6E-02	Lip20
4.641	12%	1.035	MM20
1.453	4%	0.324	MM12
3.724	11%	0.831	MM14
2.563	10%	0.572	MM17
0.000	999%	0.000	Lip13a+Lip13b
5.177	9%	1.155	MM14+Lip13a+L
3.156	10%	0.704	MM09+Lip09
5.026	11%	1.121	MM20+Lip20

DIAGNOSTICS

1 info STARTV 24
2 info's RFALSI 11
Doing Water-Scaling

MISCELLANEOUS OUTPUT

FWHM = 0.049 ppm S/N = 25
Data shift = 0.027 ppm
Ph: 11 deg -16.7 deg/ppm

CONTROL-46yo-Male-Post-checkerboard

LCModel (Version 6.3-0B) Copyright: S.W. Provencher. *Ref.: Magn. Reson. Med. 30:672-679 (1993).* *31-July-2018 13:51*

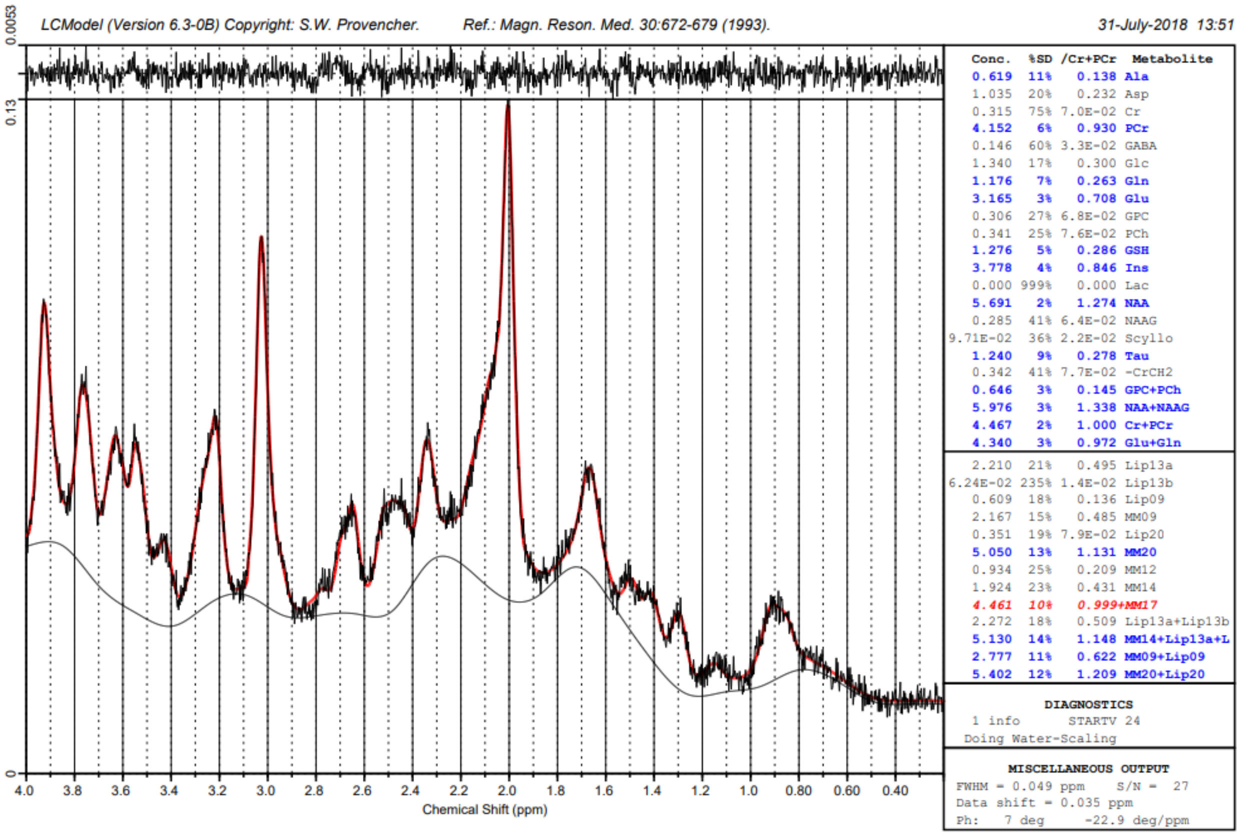

Conc.	%SD	/Cr+PCr	Metabolite
**0.619**	**11%**	**0.138**	**Ala**
1.035	20%	0.232	Asp
0.315	75%	7.0E-02	Cr
**4.152**	**6%**	**0.930**	**PCr**
0.146	60%	3.3E-02	GABA
1.340	17%	0.300	Glc
**1.176**	**7%**	**0.263**	**Gln**
**3.165**	**3%**	**0.708**	**Glu**
0.306	27%	6.8E-02	GPC
0.341	25%	7.6E-02	PCh
**1.276**	**5%**	**0.286**	**GSH**
**3.778**	**4%**	**0.846**	**Ins**
0.000	999%	0.000	Lac
**5.691**	**2%**	**1.274**	**NAA**
0.285	41%	6.4E-02	NAAG
9.71E-02	36%	2.2E-02	Scyllo
**1.240**	**9%**	**0.278**	**Tau**
0.342	41%	7.7E-02	-CrCH2
**0.646**	**3%**	**0.145**	**GPC+PCh**
**5.976**	**3%**	**1.338**	**NAA+NAAG**
**4.467**	**2%**	**1.000**	**Cr+PCr**
**4.340**	**3%**	**0.972**	**Glu+Gln**

2.210	21%	0.495	Lip13a
6.24E-02	235%	1.4E-02	Lip13b
0.609	18%	0.136	Lip09
2.167	15%	0.485	MM09
0.351	19%	7.9E-02	Lip20
**5.050**	**13%**	**1.131**	**MM20**
0.934	25%	0.209	MM12
1.924	23%	0.431	MM14
4.461	*10%*	*0.999+MM17*	
2.272	18%	0.509	Lip13a+Lip13b
**5.130**	**14%**	**1.148**	**MM14+Lip13a+L**
**2.777**	**11%**	**0.622**	**MM09+Lip09**
**5.402**	**12%**	**1.209**	**MM20+Lip20**

DIAGNOSTICS

1 info STARTV 24

Doing Water-Scaling

MISCELLANEOUS OUTPUT

FWHM = 0.049 ppm S/N = 27

Data shift = 0.035 ppm

Ph: 7 deg -22.9 deg/ppm

CONTROL-28yo-Female-Pre-checkerboard

LCModel (Version 6.3-0B) Copyright: S.W. Provencher. Ref.: Magn. Reson. Med. 30:672-679 (1993). 31-July-2018 13:52

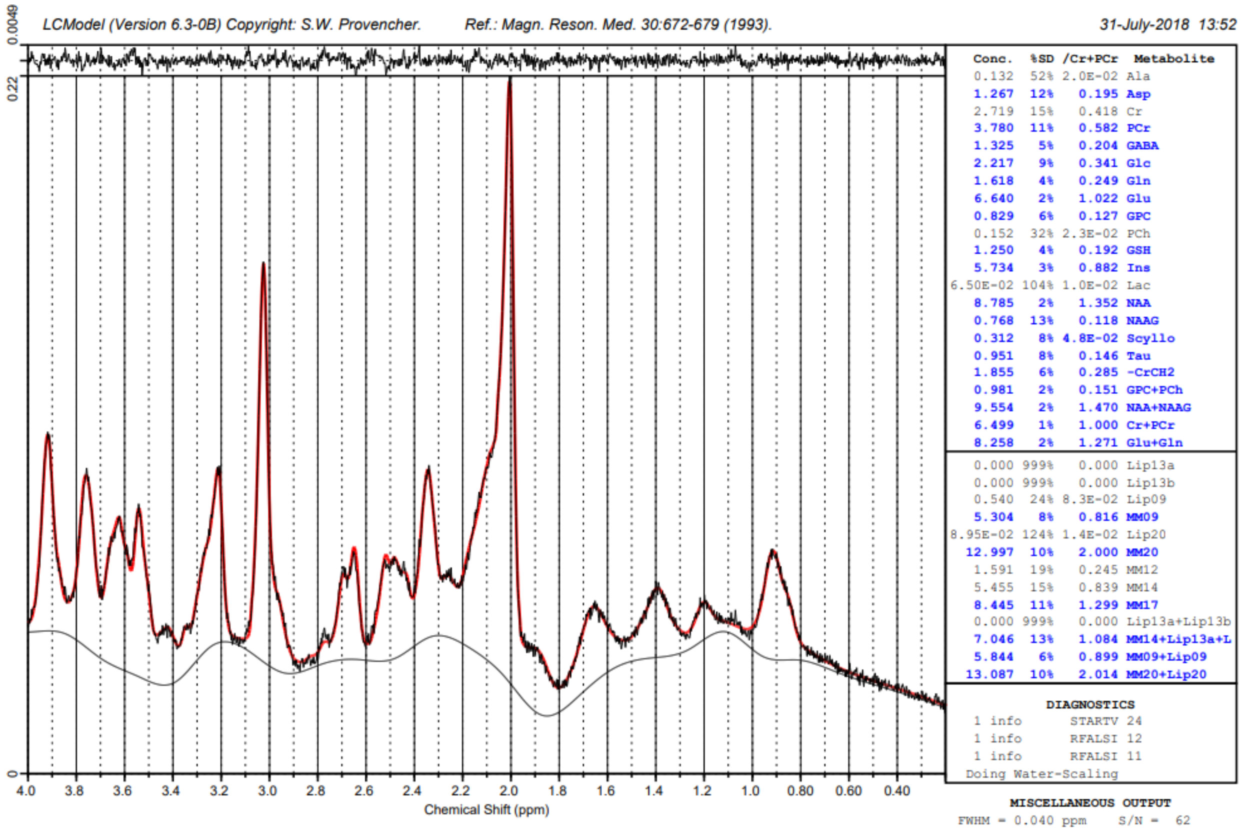

Conc.	%SD	/Cr+PCr	Metabolite
0.132	52%	2.0E-02	Ala
1.267	12%	0.195	Asp
2.719	15%	0.418	Cr
3.780	11%	0.582	PCr
1.325	5%	0.204	GABA
2.217	9%	0.341	Glc
1.618	4%	0.249	Gln
6.640	2%	1.022	Glu
0.829	6%	0.127	GPC
0.152	32%	2.3E-02	PCh
1.250	4%	0.192	GSH
5.734	3%	0.882	Ins
6.50E-02	104%	1.0E-02	Lac
8.785	2%	1.352	NAA
0.768	13%	0.118	NAAG
0.312	8%	4.8E-02	Scyllo
0.951	8%	0.146	Tau
1.855	6%	0.285	-CrCH2
0.981	2%	0.151	GPC+PCh
9.554	2%	1.470	NAA+NAAG
6.499	1%	1.000	Cr+PCr
8.258	2%	1.271	Glu+Gln
0.000	999%	0.000	Lip13a
0.000	999%	0.000	Lip13b
0.540	24%	8.3E-02	Lip09
5.304	8%	0.816	MM09
8.95E-02	124%	1.4E-02	Lip20
12.997	10%	2.000	MM20
1.591	19%	0.245	MM12
5.455	15%	0.839	MM14
8.445	11%	1.299	MM17
0.000	999%	0.000	Lip13a+Lip13b
7.046	13%	1.084	MM14+Lip13a+L
5.844	6%	0.899	MM09+Lip09
13.087	10%	2.014	MM20+Lip20

DIAGNOSTICS

1 info STARTV 24
1 info RFALSI 12
1 info RFALSI 11
Doing Water-Scaling

MISCELLANEOUS OUTPUT

FWHM = 0.040 ppm S/N = 62
Data shift = 0.032 ppm

CONTROL-28yo-Female-Pre-checkerboard

LCModel (Version 6.3-0B) Copyright: S.W. Provencher. Ref.: Magn. Reson. Med. 30:672-679 (1993). *31-July-2018 13:52*

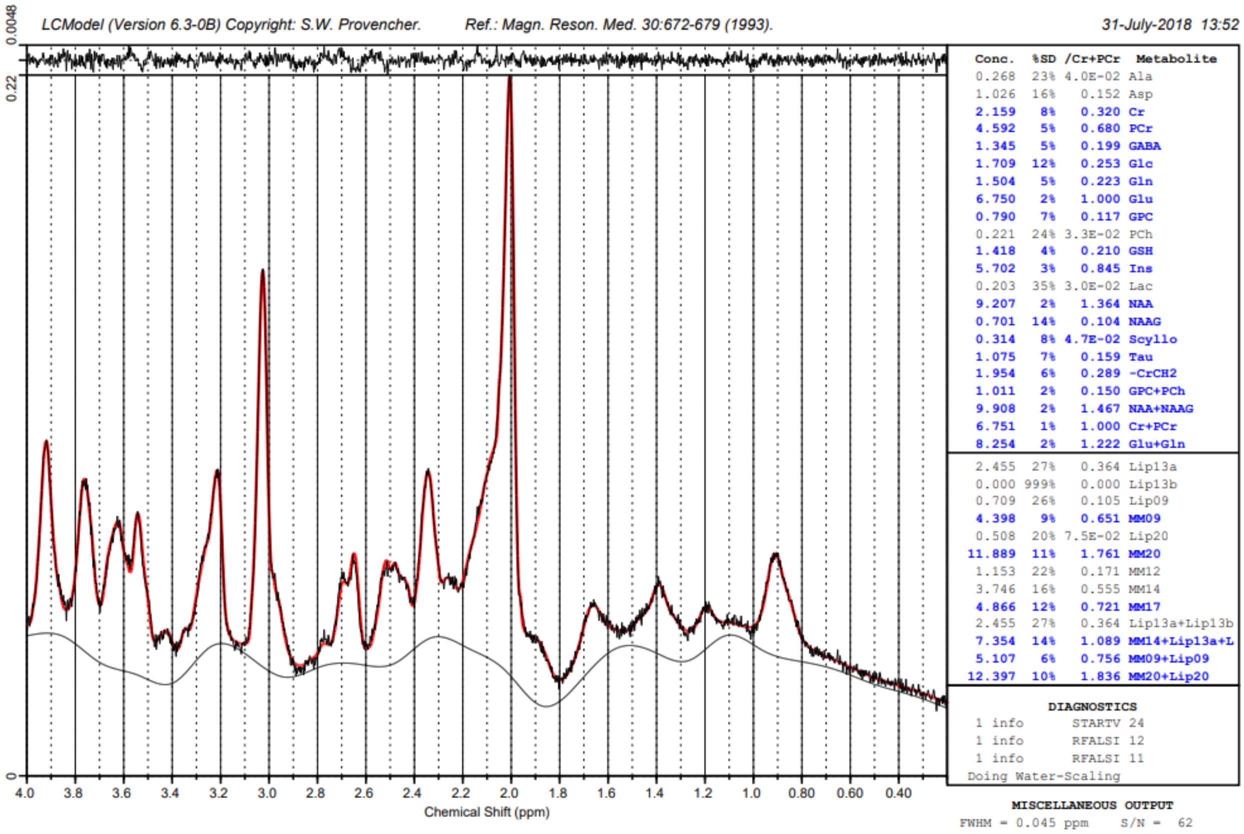

Conc.	%SD	/Cr+PCr	Metabolite
0.268	23%	4.0E-02	Ala
1.026	16%	0.152	Asp
2.159	8%	0.320	Cr
4.592	5%	0.680	PCr
1.345	5%	0.199	GABA
1.709	12%	0.253	Glc
1.504	5%	0.223	Gln
6.750	2%	1.000	Glu
0.790	7%	0.117	GPC
0.221	24%	3.3E-02	PCh
1.418	4%	0.210	GSH
5.702	3%	0.845	Ins
0.203	35%	3.0E-02	Lac
9.207	2%	1.364	NAA
0.701	14%	0.104	NAAG
0.314	8%	4.7E-02	Scyllo
1.075	7%	0.159	Tau
1.954	6%	0.289	-CrCH2
1.011	2%	0.150	GPC+PCh
9.908	2%	1.467	NAA+NAAG
6.751	1%	1.000	Cr+PCr
8.254	2%	1.222	Glu+Gln
2.455	27%	0.364	Lip13a
0.000	999%	0.000	Lip13b
0.709	26%	0.105	Lip09
4.398	9%	0.651	MM09
0.508	20%	7.5E-02	Lip20
11.889	11%	1.761	MM20
1.153	22%	0.171	MM12
3.746	16%	0.555	MM14
4.866	12%	0.721	MM17
2.455	27%	0.364	Lip13a+Lip13b
7.354	14%	1.089	MM14+Lip13a+L
5.107	6%	0.756	MM09+Lip09
12.397	10%	1.836	MM20+Lip20

DIAGNOSTICS

1 info STARTV 24
1 info RFALSI 12
1 info RFALSI 11
Doing Water-Scaling

MISCELLANEOUS OUTPUT

FWHM = 0.045 ppm S/N = 62
Data shift = 0.030 ppm

MIGRAINE WITH AURA-27yo-Female-Pre-checkerboard

LCModel (Version 6.3-0B) Copyright: S.W. Provencher. *Ref.: Magn. Reson. Med. 30:672-679 (1993).* *31-July-2018 13:52*

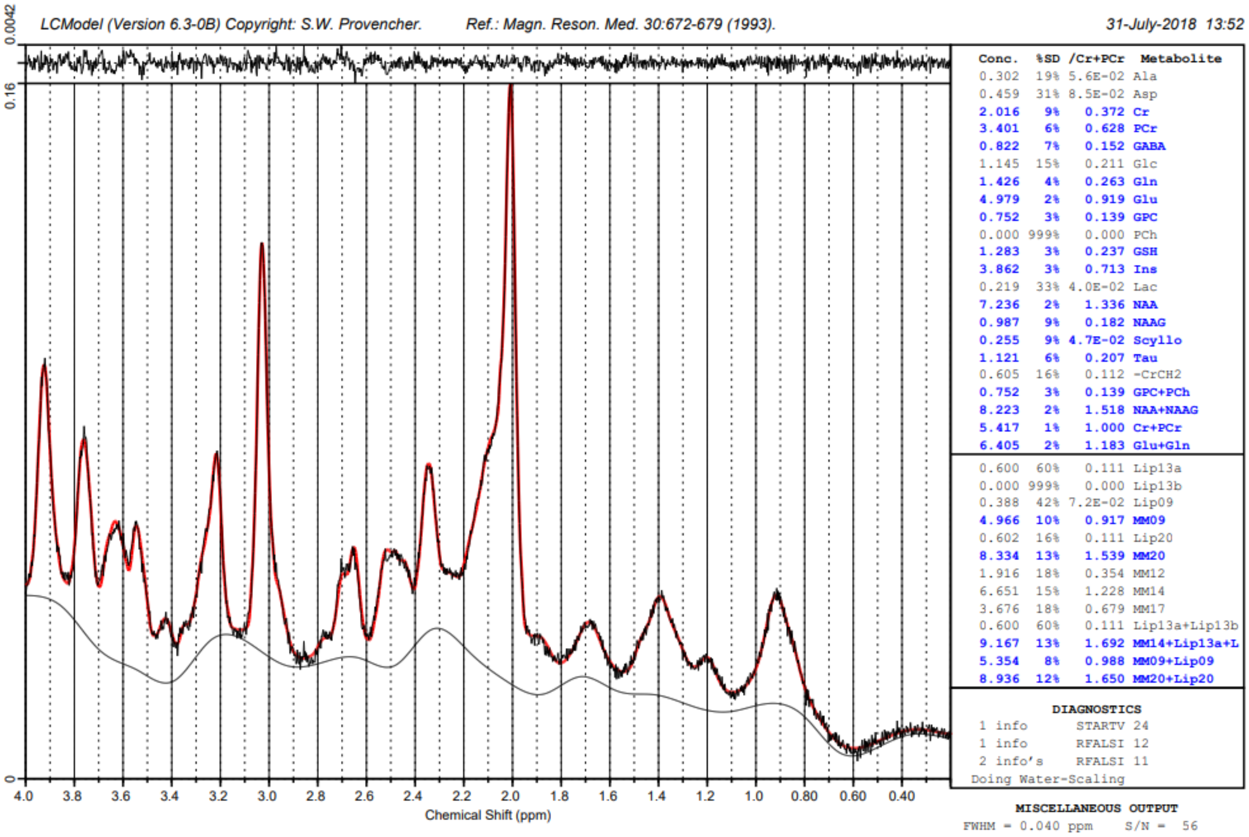

Conc.	%SD	/Cr+PCr	Metabolite
0.302	19%	5.6E-02	Ala
0.459	31%	8.5E-02	Asp
2.016	9%	0.372	Cr
3.401	6%	0.628	PCr
0.822	7%	0.152	GABA
1.145	15%	0.211	Glc
1.426	4%	0.263	Gln
4.979	2%	0.919	Glu
0.752	3%	0.139	GPC
0.000	999%	0.000	PCh
1.283	3%	0.237	GSH
3.862	3%	0.713	Ins
0.219	33%	4.0E-02	Lac
7.236	2%	1.336	NAA
0.987	9%	0.182	NAAG
0.255	9%	4.7E-02	Scyllo
1.121	6%	0.207	Tau
0.605	16%	0.112	-CrCH2
0.752	3%	0.139	GPC+PCh
8.223	2%	1.518	NAA+NAAG
5.417	1%	1.000	Cr+PCr
6.405	2%	1.183	Glu+Gln
0.600	60%	0.111	Lip13a
0.000	999%	0.000	Lip13b
0.388	42%	7.2E-02	Lip09
4.966	10%	0.917	MM09
0.602	16%	0.111	Lip20
8.334	13%	1.539	MM20
1.916	18%	0.354	MM12
6.651	15%	1.228	MM14
3.676	18%	0.679	MM17
0.600	60%	0.111	Lip13a+Lip13b
9.167	13%	1.692	MM14+Lip13a+L
5.354	8%	0.988	MM09+Lip09
8.936	12%	1.650	MM20+Lip20

DIAGNOSTICS

1 info STARTV 24
1 info RFALSI 12
2 info's RFALSI 11
Doing Water-Scaling

MISCELLANEOUS OUTPUT

FWHM = 0.040 ppm S/N = 56
Data shift = 0.017 ppm

MIGRAINE WITH AURA-27yo-Female-Post-checkerboard

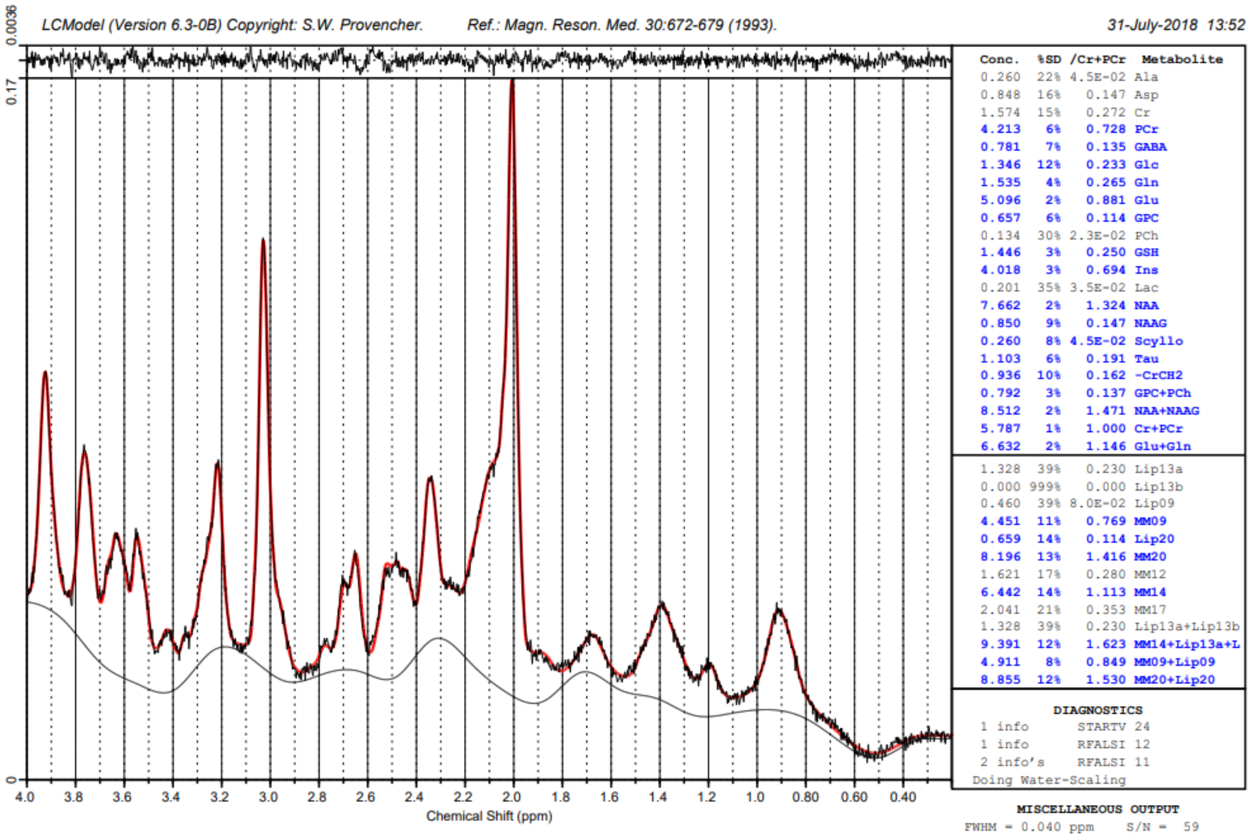

Conc.	%SD	/Cr+PCr	Metabolite
0.260	22%	4.5E-02	Ala
0.848	16%	0.147	Asp
1.574	15%	0.272	Cr
4.213	6%	0.728	PCr
0.781	7%	0.135	GABA
1.346	12%	0.233	Glc
1.535	4%	0.265	Gln
5.096	2%	0.881	Glu
0.657	6%	0.114	GPC
0.134	30%	2.3E-02	PCh
1.446	3%	0.250	GSH
4.018	3%	0.694	Ins
0.201	35%	3.5E-02	Lac
7.662	2%	1.324	NAA
0.850	9%	0.147	NAAG
0.260	8%	4.5E-02	Scyllo
1.103	6%	0.191	Tau
0.936	10%	0.162	-CrCH2
0.792	3%	0.137	GPC+PCh
8.512	2%	1.471	NAA+NAAG
5.787	1%	1.000	Cr+PCr
6.632	2%	1.146	Glu+Gln
1.328	39%	0.230	Lip13a
0.000	999%	0.000	Lip13b
0.460	39%	8.0E-02	Lip09
4.451	11%	0.769	MM09
0.659	14%	0.114	Lip20
8.196	13%	1.416	MM20
1.621	17%	0.280	MM12
6.442	14%	1.113	MM14
2.041	21%	0.353	MM17
1.328	39%	0.230	Lip13a+Lip13b
9.391	12%	1.623	MM14+Lip13a+L
4.911	8%	0.849	MM09+Lip09
8.855	12%	1.530	MM20+Lip20

DIAGNOSTICS

1 info STARTV 24
1 info RFALSI 12
2 info's RFALSI 11
Doing Water-Scaling

MISCELLANEOUS OUTPUT

FWHM = 0.040 ppm S/N = 59
Data shift = 0.025 ppm

MIGRAINE WITH AURA-42yo-Male-Pre-checkerboard

LCModel (Version 6.3-0B) Copyright: S.W. Provencher. *Ref.: Magn. Reson. Med. 30:672-679 (1993).* *31-July-2018 13:53*

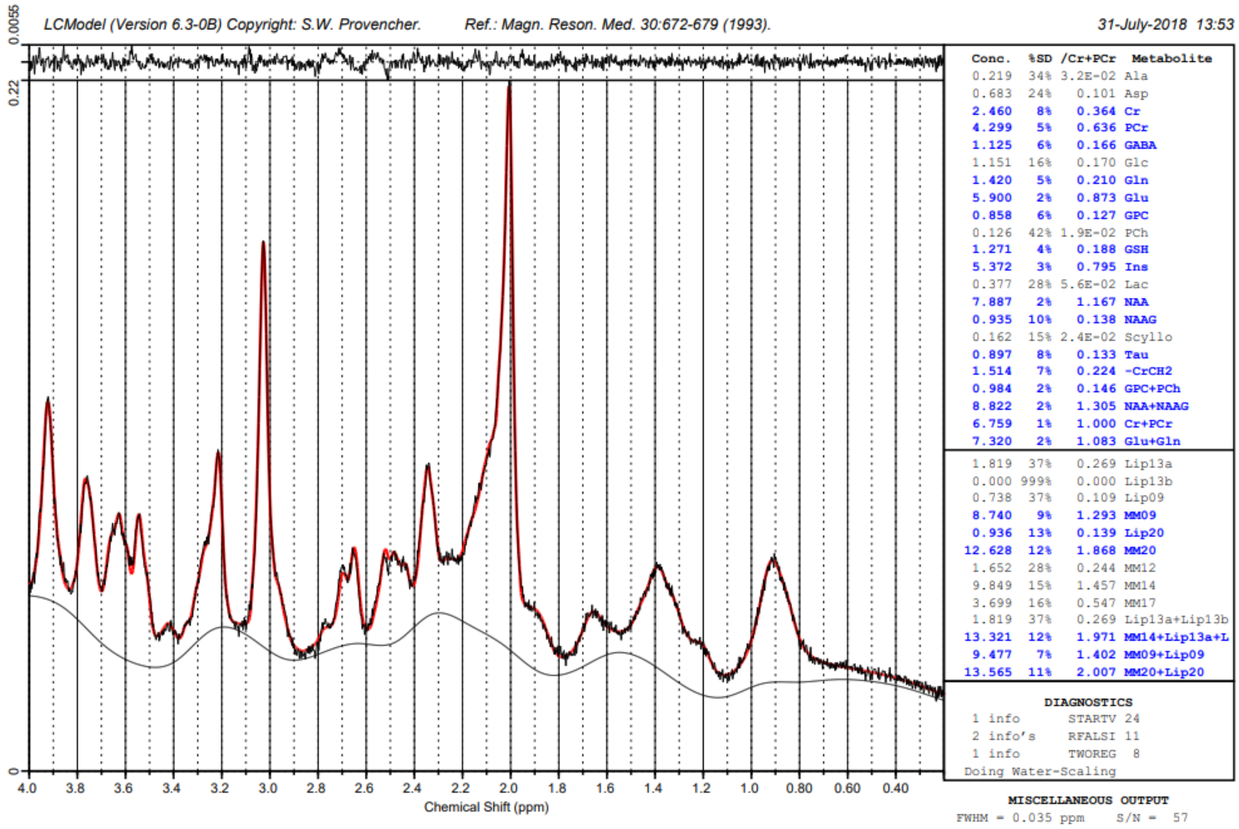

Conc.	%SD	/Cr+PCr	Metabolite
0.219	34%	3.2E-02	Ala
0.683	24%	0.101	Asp
2.460	8%	0.364	Cr
4.299	5%	0.636	PCr
1.125	6%	0.166	GABA
1.151	16%	0.170	Glc
1.420	5%	0.210	Gln
5.900	2%	0.873	Glu
0.858	6%	0.127	GPC
0.126	42%	1.9E-02	PCh
1.271	4%	0.188	GSH
5.372	3%	0.795	Ins
0.377	28%	5.6E-02	Lac
7.887	2%	1.167	NAA
0.935	10%	0.138	NAAG
0.162	15%	2.4E-02	Scyllo
0.897	8%	0.133	Tau
1.514	7%	0.224	-CrCH2
0.984	2%	0.146	GPC+PCh
8.822	2%	1.305	NAA+NAAG
6.759	1%	1.000	Cr+PCr
7.320	2%	1.083	Glu+Gln
1.819	37%	0.269	Lip13a
0.000	999%	0.000	Lip13b
0.738	37%	0.109	Lip09
8.740	9%	1.293	MM09
0.936	13%	0.139	Lip20
12.628	12%	1.868	MM20
1.652	28%	0.244	MM12
9.849	15%	1.457	MM14
3.699	16%	0.547	MM17
1.819	37%	0.269	Lip13a+Lip13b
13.321	12%	1.971	MM14+Lip13a+L
9.477	7%	1.402	MM09+Lip09
13.565	11%	2.007	MM20+Lip20

DIAGNOSTICS

1 info STARTV 24
2 info's RFALSI 11
1 info TWOREG 8
Doing Water-Scaling

MISCELLANEOUS OUTPUT

FWHM = 0.035 ppm S/N = 57
Data shift = 0.020 ppm

MIGRAINE WITH AURA-42yo-Male-Post-checkerboard

LCModel (Version 6.3-0B) Copyright: S.W. Provencher. *Ref.: Magn. Reson. Med. 30:672-679 (1993).* *31-July-2018 13:53*

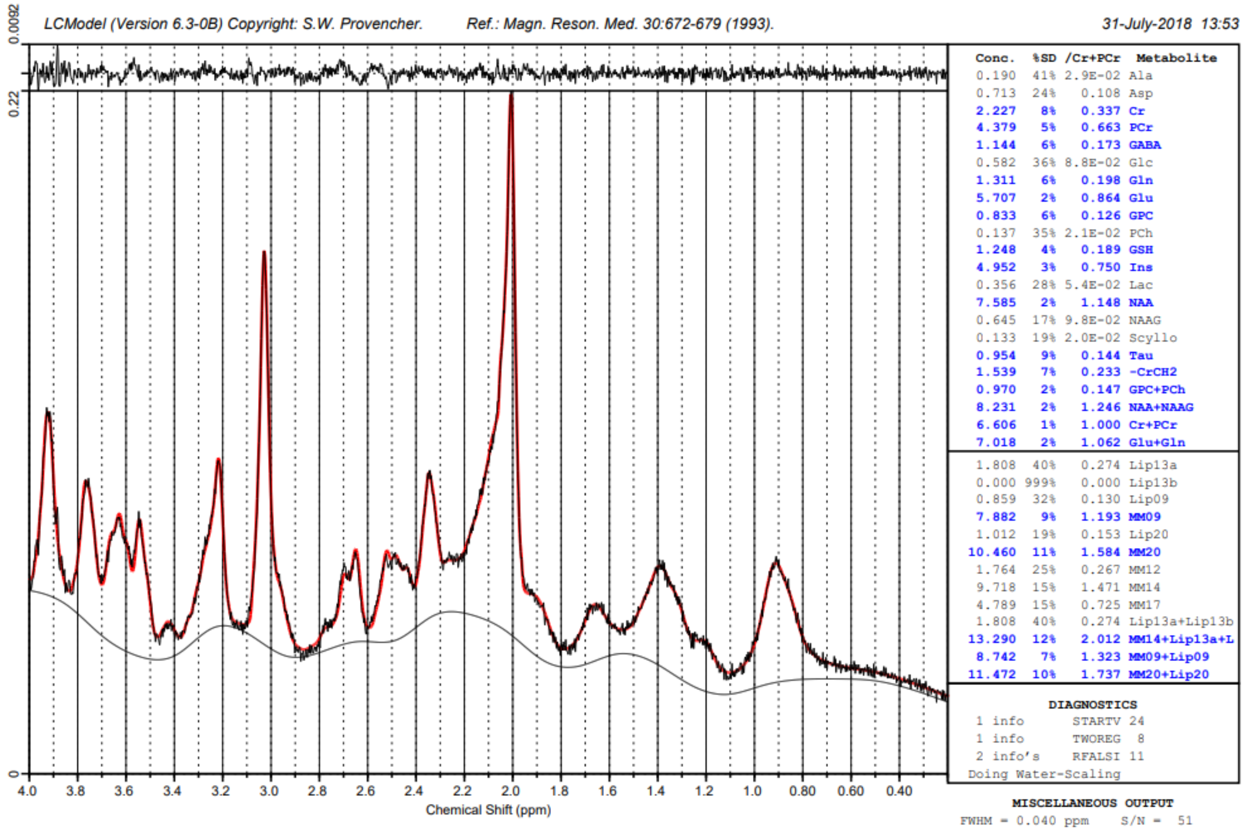

Conc.	%SD	/Cr+PCr	Metabolite
0.190	41%	2.9E-02	Ala
0.713	24%	0.108	Asp
2.227	8%	0.337	Cr
4.379	5%	0.663	PCr
1.144	6%	0.173	GABA
0.582	36%	8.8E-02	Glc
1.311	6%	0.198	Gln
5.707	2%	0.864	Glu
0.833	6%	0.126	GPC
0.137	35%	2.1E-02	PCh
1.248	4%	0.189	GSH
4.952	3%	0.750	Ins
0.356	28%	5.4E-02	Lac
7.585	2%	1.148	NAA
0.645	17%	9.8E-02	NAAG
0.133	19%	2.0E-02	Scyllo
0.954	9%	0.144	Tau
1.539	7%	0.233	-CrCH2
0.970	2%	0.147	GPC+PCh
8.231	2%	1.246	NAA+NAAG
6.606	1%	1.000	Cr+PCr
7.018	2%	1.062	Glu+Gln
1.808	40%	0.274	Lip13a
0.000	999%	0.000	Lip13b
0.859	32%	0.130	Lip09
7.882	9%	1.193	MM09
1.012	19%	0.153	Lip20
10.460	11%	1.584	MM20
1.764	25%	0.267	MM12
9.718	15%	1.471	MM14
4.789	15%	0.725	MM17
1.808	40%	0.274	Lip13a+Lip13b
13.290	12%	2.012	MM14+Lip13a+L
8.742	7%	1.323	MM09+Lip09
11.472	10%	1.737	MM20+Lip20

DIAGNOSTICS

1 info	STARTV 24
1 info	TWOREG 8
2 info's	RFALSI 11

Doing Water-Scaling

MISCELLANEOUS OUTPUT

FWHM = 0.040 ppm S/N = 51

Data shift = 0.022 ppm

MIGRAINE WITH AURA-42yo-Male-Pre-checkerboard

LCModel (Version 6.3-0B) Copyright: S.W. Provencher. Ref.: Magn. Reson. Med. 30:672-679 (1993). 31-July-2018 13:53

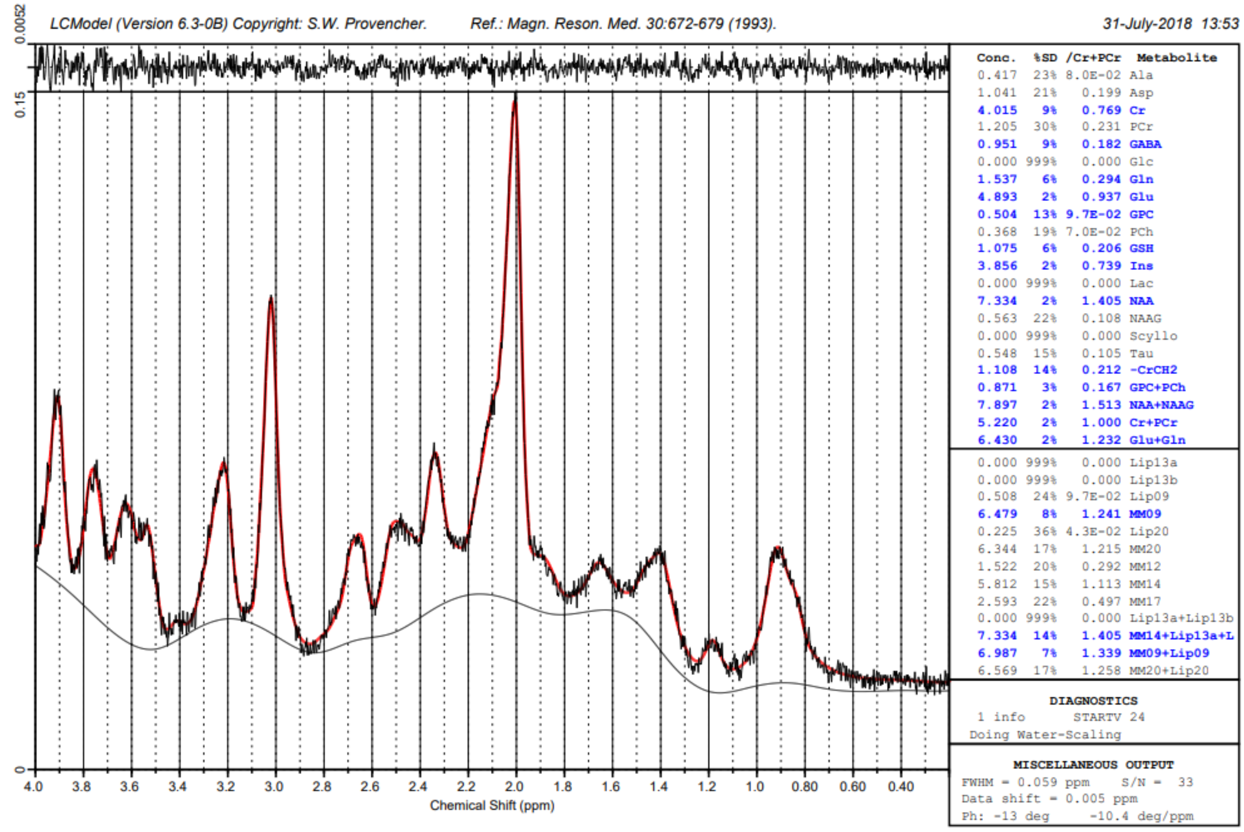

Conc.	%SD	/Cr+PCr	Metabolite
0.417	23%	8.0E-02	Ala
1.041	21%	0.199	Asp
**4.015**	**9%**	**0.769**	**Cr**
1.205	30%	0.231	PCr
**0.951**	**9%**	**0.182**	**GABA**
0.000	999%	0.000	Glc
**1.537**	**6%**	**0.294**	**Gln**
**4.893**	**2%**	**0.937**	**Glu**
**0.504**	**13%**	**9.7E-02**	**GPC**
0.368	19%	7.0E-02	PCh
**1.075**	**6%**	**0.206**	**GSH**
**3.856**	**2%**	**0.739**	**Ins**
0.000	999%	0.000	Lac
**7.334**	**2%**	**1.405**	**NAA**
0.563	22%	0.108	NAAG
0.000	999%	0.000	Scyllo
0.548	15%	0.105	Tau
**1.108**	**14%**	**0.212**	**-CrCH2**
**0.871**	**3%**	**0.167**	**GPC+PCh**
**7.897**	**2%**	**1.513**	**NAA+NAAG**
**5.220**	**2%**	**1.000**	**Cr+PCr**
**6.430**	**2%**	**1.232**	**Glu+Gln**
0.000	999%	0.000	Lip13a
0.000	999%	0.000	Lip13b
0.508	24%	9.7E-02	Lip09
**6.479**	**8%**	**1.241**	**MM09**
0.225	36%	4.3E-02	Lip20
6.344	17%	1.215	MM20
1.522	20%	0.292	MM12
5.812	15%	1.113	MM14
2.593	22%	0.497	MM17
0.000	999%	0.000	Lip13a+Lip13b
**7.334**	**14%**	**1.405**	**MM14+Lip13a+L**
**6.987**	**7%**	**1.339**	**MM09+Lip09**
6.569	17%	1.258	MM20+Lip20

DIAGNOSTICS

1 info STARTV 24

Doing Water-Scaling

MISCELLANEOUS OUTPUT

FWHM = 0.059 ppm S/N = 33

Data shift = 0.005 ppm

Ph: -13 deg -10.4 deg/ppm

MIGRAINE WITH AURA-42yo-Male-Post-checkerboard

LCModel (Version 6.3-0B) Copyright: S.W. Provencher. *Ref.: Magn. Reson. Med. 30:672-679 (1993).* *31-July-2018 13:53*

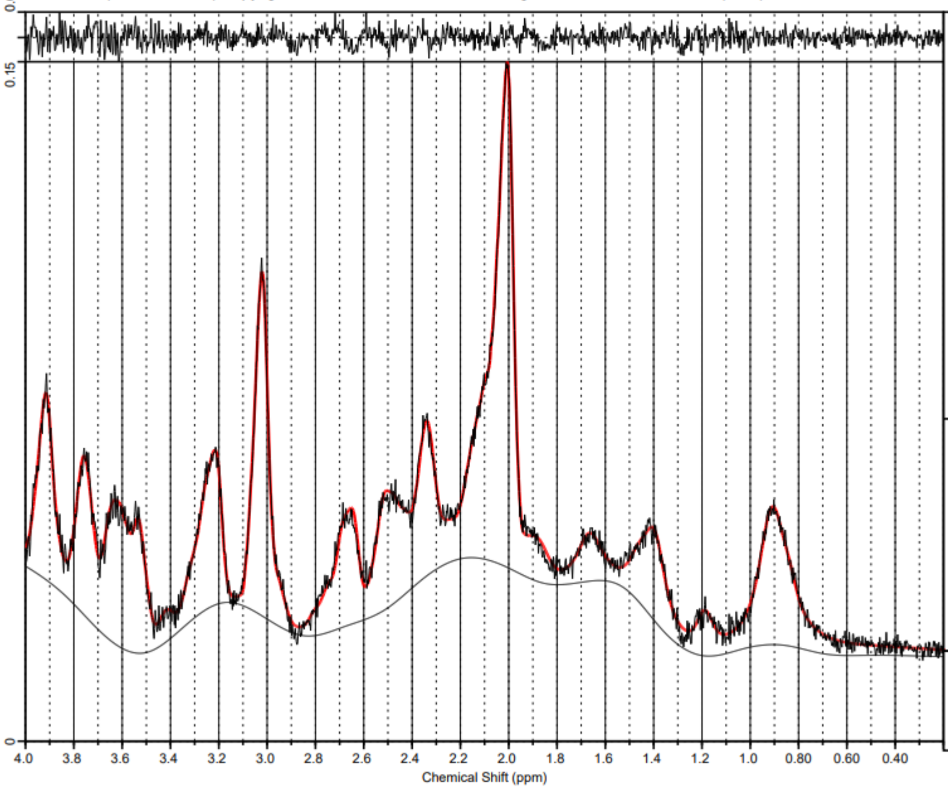

Conc.	%SD	/Cr+PCr	Metabolite
0.461	22%	8.2E-02	Ala
**1.925**	**12%**	**0.342**	**Asp**
1.640	25%	0.292	Cr
**3.985**	**11%**	**0.708**	**PCr**
**0.837**	**10%**	**0.149**	**GABA**
0.000	999%	0.000	Glc
**1.508**	**6%**	**0.268**	**Gln**
**5.018**	**2%**	**0.892**	**Glu**
**0.729**	**12%**	**0.130**	**GPC**
7.30E-02	130%	1.3E-02	PCh
**1.138**	**6%**	**0.202**	**GSH**
**4.211**	**2%**	**0.748**	**Ins**
0.000	999%	0.000	Lac
**7.504**	**2%**	**1.334**	**NAA**
0.681	18%	0.121	NAAG
0.185	22%	3.3E-02	Scyllo
**0.947**	**13%**	**0.168**	**Tau**
**1.665**	**11%**	**0.296**	**-CrCH2**
**0.802**	**3%**	**0.143**	**GPC+PCh**
**8.185**	**2%**	**1.455**	**NAA+NAAG**
**5.625**	**2%**	**1.000**	**Cr+PCr**
**6.526**	**2%**	**1.160**	**Glu+Gln**
0.000	999%	0.000	Lip13a
0.000	999%	0.000	Lip13b
0.373	33%	6.6E-02	Lip09
**6.747**	**8%**	**1.199**	**MM09**
0.430	*19%*	*7.6E-02*	*+Lip20*
3.070	24%	0.546	MM20
1.401	22%	0.249	MM12
4.608	15%	0.819	MM14
2.736	21%	0.486	MM17
0.000	999%	0.000	Lip13a+Lip13b
**6.010**	**14%**	**1.068**	**MM14+Lip13a+L**
**7.121**	**7%**	**1.266**	**MM09+Lip09**
3.499	21%	0.622	MM20+Lip20

DIAGNOSTICS

1 info STARTV 24
1 info RFALSI 11
1 info TWOREG 8
Doing Water-Scaling

MISCELLANEOUS OUTPUT

FWHM = 0.059 ppm S/N = 32
Data shift = 0.005 ppm

CONTROL-35yo-Male-Pre-checkerboard

LCModel (Version 6.3-0B) Copyright: S.W. Provencher. *Ref.: Magn. Reson. Med. 30:672-679 (1993).* *31-July-2018 13:54*

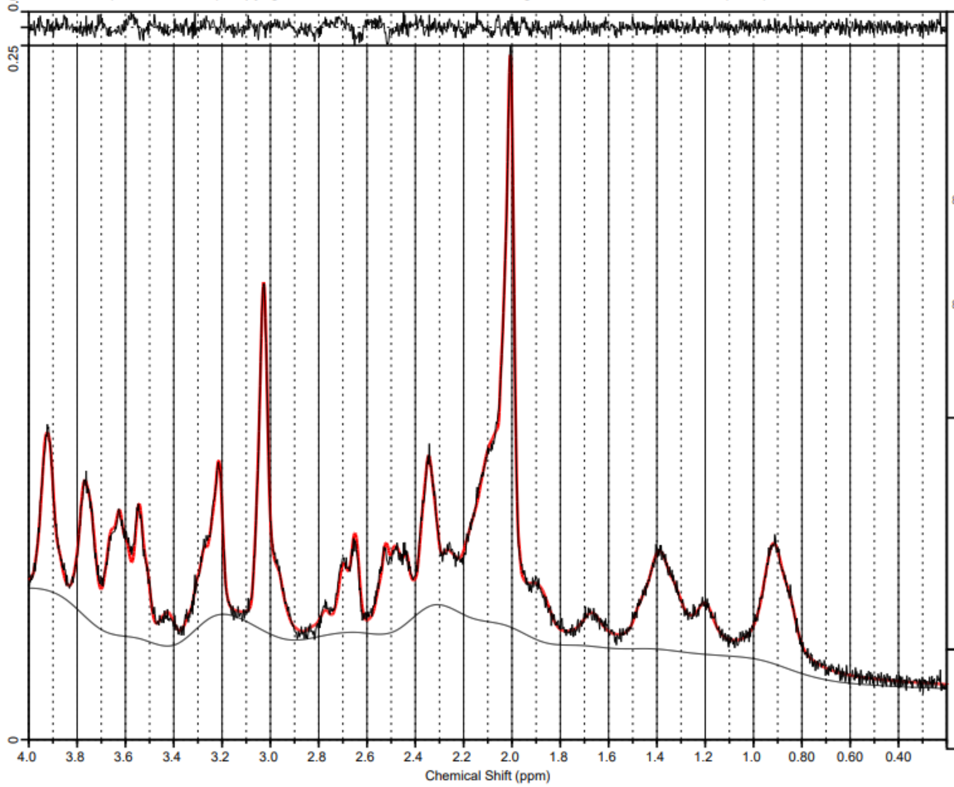

Conc.	%SD	/Cr+PCr	Metabolite
0.328	23%	5.4E-02	Ala
0.963	18%	0.160	Asp
2.410	8%	0.399	Cr
3.625	6%	0.601	PCr
1.264	6%	0.210	GABA
0.783	27%	0.130	Glc
1.533	5%	0.254	Gln
6.289	2%	1.042	Glu
0.846	6%	0.140	GPC
8.21E-02	65%	1.4E-02	PCh
1.282	4%	0.212	GSH
5.188	3%	0.860	Ins
0.335	29%	5.6E-02	Lac
8.248	2%	1.367	NAA
0.709	15%	0.117	NAAG
8.98E-02	29%	1.5E-02	Scyllo
0.855	10%	0.142	Tau
1.197	10%	0.198	-CrCH2
0.928	3%	0.154	GPC+PCh
8.957	2%	1.484	NAA+NAAG
6.035	1%	1.000	Cr+PCr
7.822	2%	1.296	Glu+Gln

0.916	57%	0.152	Lip13a
0.000	999%	0.000	Lip13b
0.658	34%	0.109	Lip09
7.609	9%	1.261	MM09
1.013	23%	0.168	Lip20
13.877	12%	2.299	MM20
2.632	18%	0.436	MM12
9.949	13%	1.649	MM14
2.251	24%	0.373	MM17
0.916	57%	0.152	Lip13a+Lip13b
13.497	12%	2.237	MM14+Lip13a+L
8.267	7%	1.370	MM09+Lip09
14.889	11%	2.467	MM20+Lip20

DIAGNOSTICS

1 info	STARTV	24
1 info	TWOREG	8
1 info	RFALSI	12

Doing Water-Scaling

MISCELLANEOUS OUTPUT

FWHM = 0.035 ppm S/N = 52

Data shift = 0.027 ppm

CONTROL-35yo-Male-Post-checkerboard

LCModel (Version 6.3-0B) Copyright: S.W. Provencher. Ref.: Magn. Reson. Med. 30:672-679 (1993). 31-July-2018 13:54

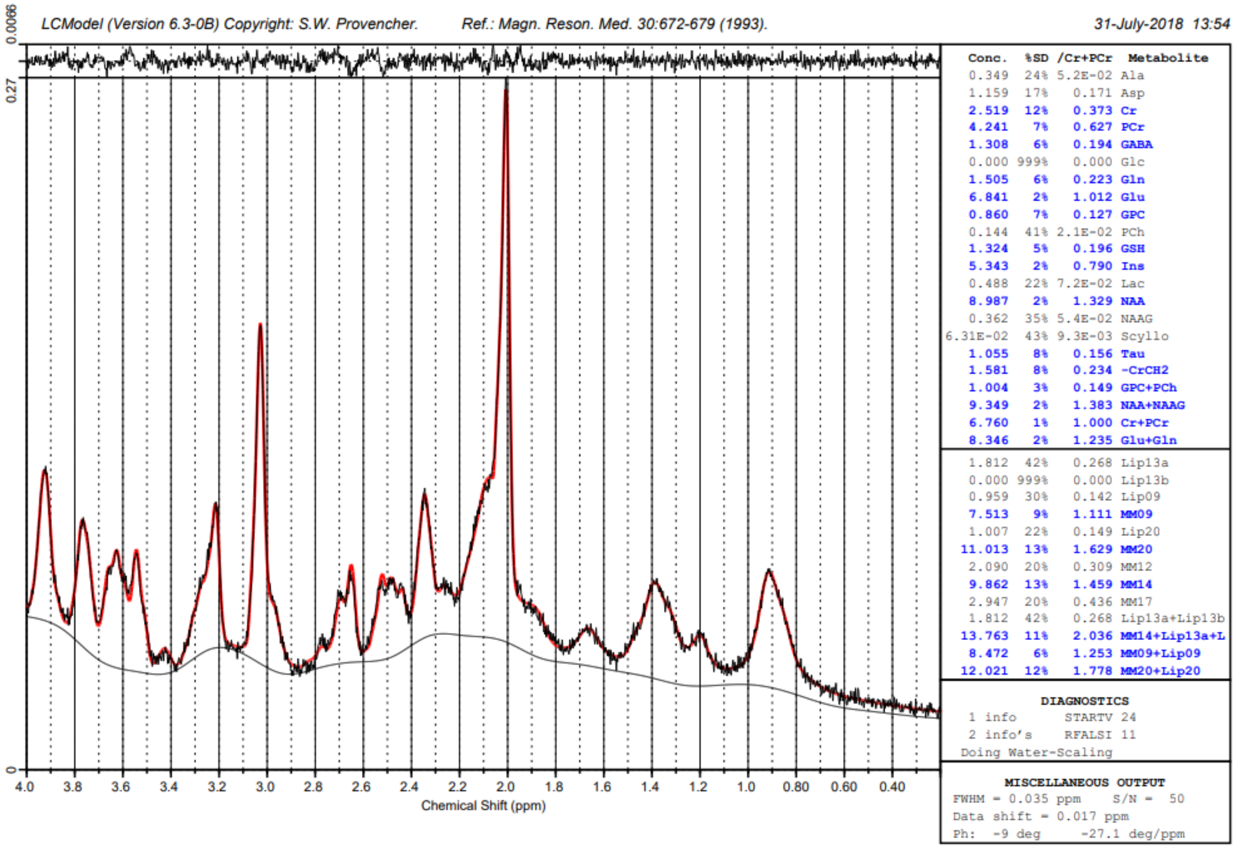

Conc.	%SD	/Cr+PCr	Metabolite
0.349	24%	5.2E-02	Ala
1.159	17%	0.171	Asp
2.519	12%	0.373	Cr
4.241	7%	0.627	PCr
1.308	6%	0.194	GABA
0.000	999%	0.000	Glc
1.505	6%	0.223	Gln
6.841	2%	1.012	Glu
0.860	7%	0.127	GPC
0.144	41%	2.1E-02	PCh
1.324	5%	0.196	GSH
5.343	2%	0.790	Ins
0.488	22%	7.2E-02	Lac
8.987	2%	1.329	NAA
0.362	35%	5.4E-02	NAAG
6.31E-02	43%	9.3E-03	Scyllo
1.055	8%	0.156	Tau
1.581	8%	0.234	-CrCH2
1.004	3%	0.149	GPC+PCh
9.349	2%	1.383	NAA+NAAG
6.760	1%	1.000	Cr+PCr
8.346	2%	1.235	Glu+Gln
1.812	42%	0.268	Lip13a
0.000	999%	0.000	Lip13b
0.959	30%	0.142	Lip09
7.513	9%	1.111	MM09
1.007	22%	0.149	Lip20
11.013	13%	1.629	MM20
2.090	20%	0.309	MM12
9.862	13%	1.459	MM14
2.947	20%	0.436	MM17
1.812	42%	0.268	Lip13a+Lip13b
13.763	11%	2.036	MM14+Lip13a+L
8.472	6%	1.253	MM09+Lip09
12.021	12%	1.778	MM20+Lip20

DIAGNOSTICS

1 info STARTV 24
2 info's RFALSI 11
Doing Water-Scaling

MISCELLANEOUS OUTPUT

FWHM = 0.035 ppm S/N = 50
Data shift = 0.017 ppm
Ph: -9 deg -27.1 deg/ppm

CONTROL-20yo-Male-Pre-checkerboard

LCModel (Version 6.3-0B) Copyright: S.W. Provencher. Ref.: Magn. Reson. Med. 30:672-679 (1993). 31-July-2018 13:55

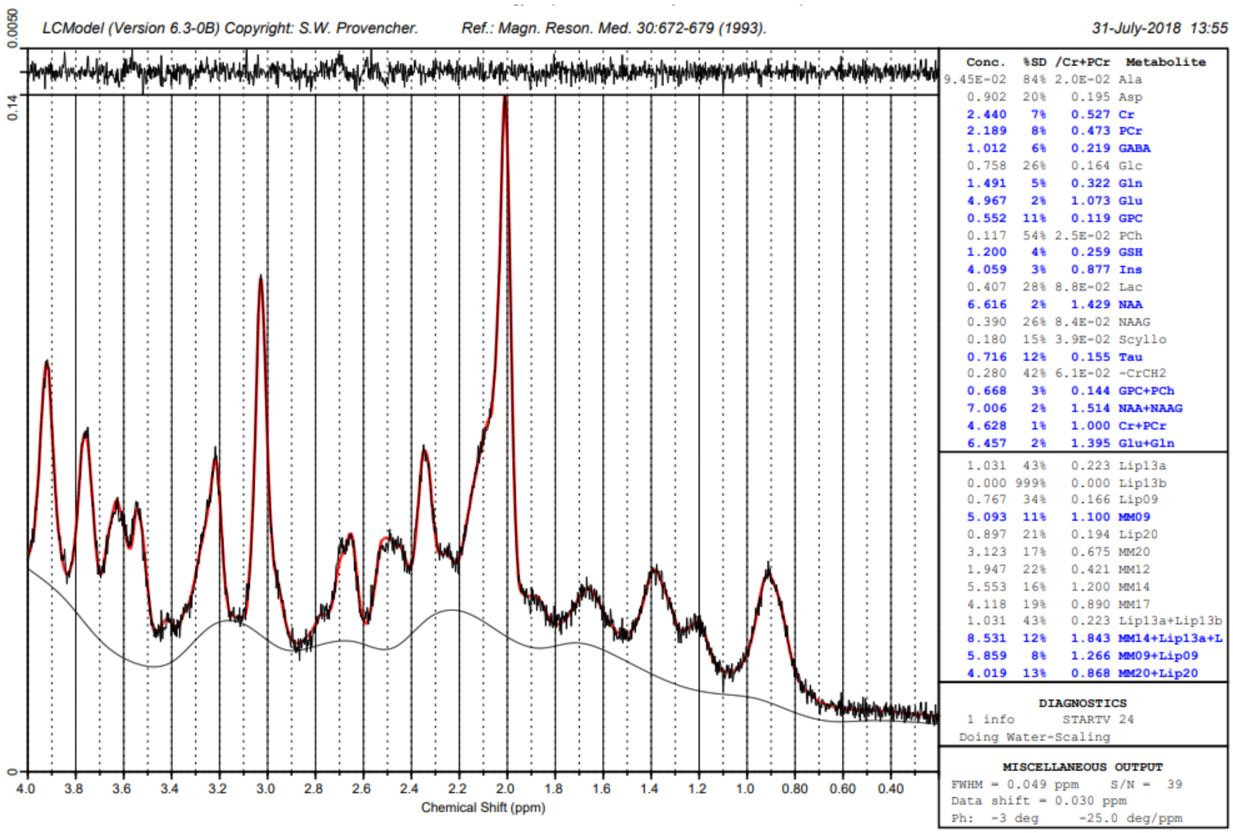

Conc.	%SD	/Cr+PCr	Metabolite
9.45E-02	84%	2.0E-02	Ala
0.902	20%	0.195	Asp
2.440	7%	0.527	Cr
2.189	8%	0.473	PCr
1.012	6%	0.219	GABA
0.758	26%	0.164	Glc
1.491	5%	0.322	Gln
4.967	2%	1.073	Glu
0.552	11%	0.119	GPC
0.117	54%	2.5E-02	PCh
1.200	4%	0.259	GSH
4.059	3%	0.877	Ins
0.407	28%	8.8E-02	Lac
6.616	2%	1.429	NAA
0.390	26%	8.4E-02	NAAG
0.180	15%	3.9E-02	Scyllo
0.716	12%	0.155	Tau
0.280	42%	6.1E-02	-CrCH2
0.668	3%	0.144	GPC+PCh
7.006	2%	1.514	NAA+NAAG
4.628	1%	1.000	Cr+PCr
6.457	2%	1.395	Glu+Gln
1.031	43%	0.223	Lip13a
0.000	999%	0.000	Lip13b
0.767	34%	0.166	Lip09
5.093	11%	1.100	MM09
0.897	21%	0.194	Lip20
3.123	17%	0.675	MM20
1.947	22%	0.421	MM12
5.553	16%	1.200	MM14
4.118	19%	0.890	MM17
1.031	43%	0.223	Lip13a+Lip13b
8.531	12%	1.843	MM14+Lip13a+L
5.859	8%	1.266	MM09+Lip09
4.019	13%	0.868	MM20+Lip20

DIAGNOSTICS

1 info STARTV 24

Doing Water-Scaling

MISCELLANEOUS OUTPUT

FWHM = 0.049 ppm S/N = 39

Data shift = 0.030 ppm

Ph: -3 deg -25.0 deg/ppm

CONTROL-20yo-Male-Post-checkerboard

LCModel (Version 6.3-0B) Copyright: S.W. Provencher. *Ref.: Magn. Reson. Med. 30:672-679 (1993).* *31-July-2018 13:55*

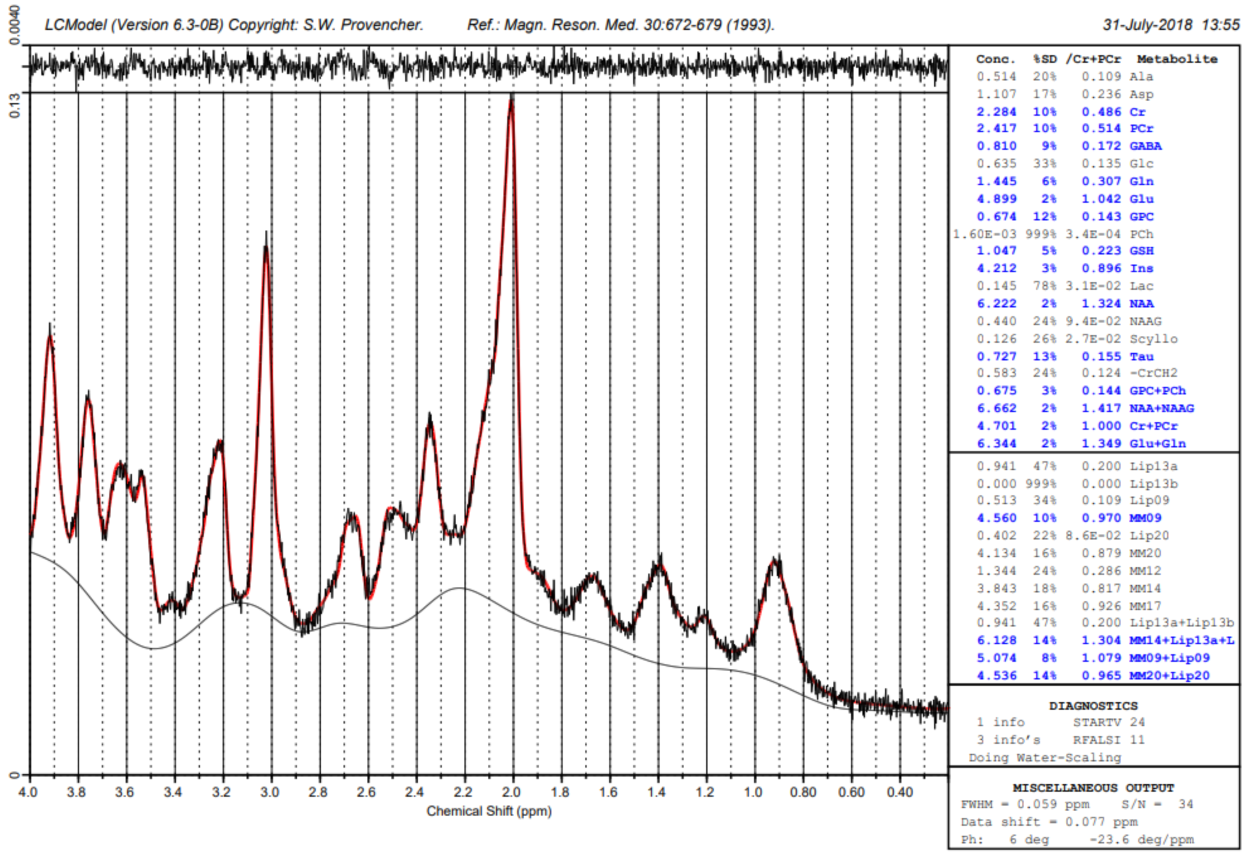

Conc.	%SD	/Cr+PCr	Metabolite
0.514	20%	0.109	Ala
1.107	17%	0.236	Asp
2.284	10%	0.486	Cr
2.417	10%	0.514	PCr
0.810	9%	0.172	GABA
0.635	33%	0.135	Glc
1.445	6%	0.307	Gln
4.899	2%	1.042	Glu
0.674	12%	0.143	GPC
1.60E-03	999%	3.4E-04	PCh
1.047	5%	0.223	GSH
4.212	3%	0.896	Ins
0.145	78%	3.1E-02	Lac
6.222	2%	1.324	NAA
0.440	24%	9.4E-02	NAAG
0.126	26%	2.7E-02	Scyllo
0.727	13%	0.155	Tau
0.583	24%	0.124	-CrCH2
0.675	3%	0.144	GPC+PCh
6.662	2%	1.417	NAA+NAAG
4.701	2%	1.000	Cr+PCr
6.344	2%	1.349	Glu+Gln
0.941	47%	0.200	Lip13a
0.000	999%	0.000	Lip13b
0.513	34%	0.109	Lip09
4.560	10%	0.970	MM09
0.402	22%	8.6E-02	Lip20
4.134	16%	0.879	MM20
1.344	24%	0.286	MM12
3.843	18%	0.817	MM14
4.352	16%	0.926	MM17
0.941	47%	0.200	Lip13a+Lip13b
6.128	14%	1.304	MM14+Lip13a+L
5.074	8%	1.079	MM09+Lip09
4.536	14%	0.965	MM20+Lip20

DIAGNOSTICS

1 info STARTV 24
3 info's RFALSI 11
Doing Water-Scaling

MISCELLANEOUS OUTPUT

FWHM = 0.059 ppm S/N = 34
Data shift = 0.077 ppm
Ph: 6 deg -23.6 deg/ppm

MIGRAINE WITHOUT AURA-30yo-Female-Pre-Checkerboard

LCModel (Version 6.3-0B) Copyright: S.W. Provencher. Ref.: Magn. Reson. Med. 30:672-679 (1993). 31-July-2018 13:55

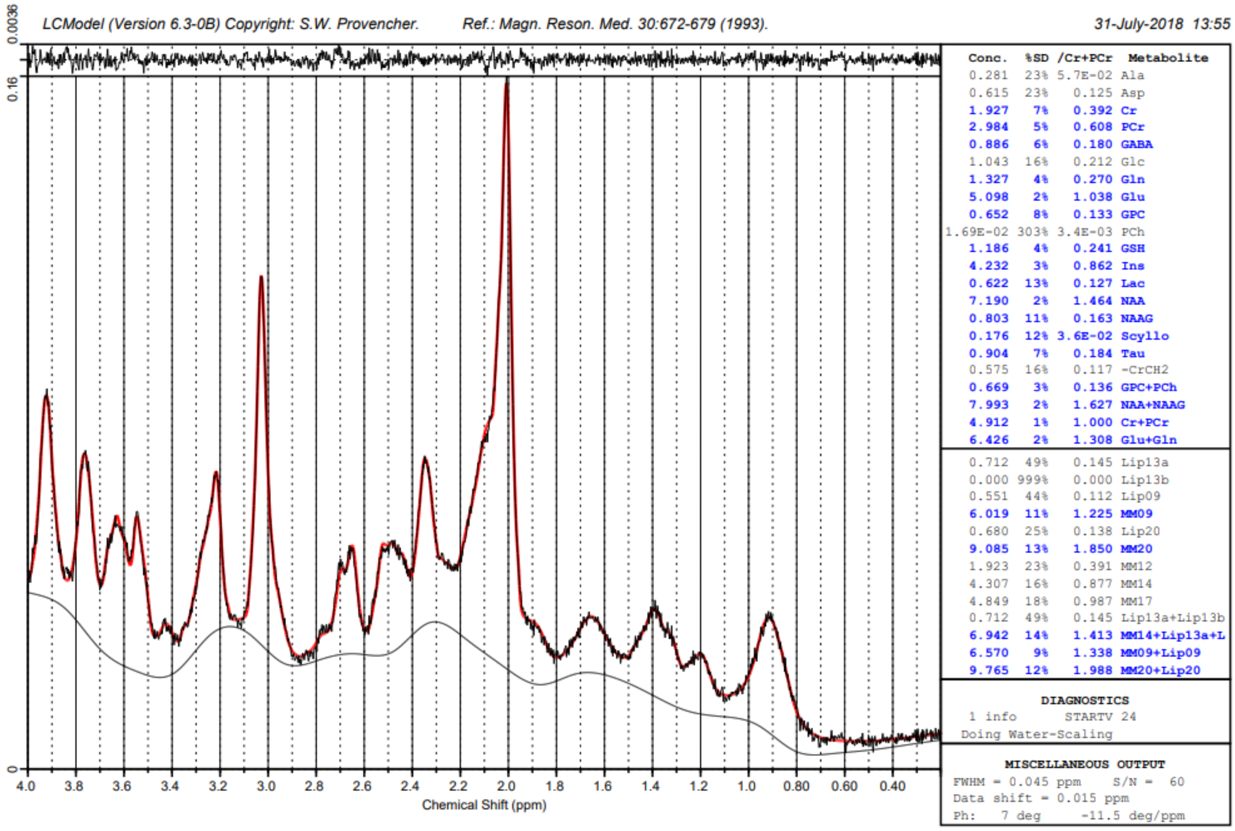

Conc.	%SD	/Cr+PCr	Metabolite
0.281	23%	5.7E-02	Ala
0.615	23%	0.125	Asp
1.927	7%	0.392	Cr
2.984	5%	0.608	PCr
0.886	6%	0.180	GABA
1.043	16%	0.212	Glc
1.327	4%	0.270	Gln
5.098	2%	1.038	Glu
0.652	8%	0.133	GPC
1.69E-02	303%	3.4E-03	PCh
1.186	4%	0.241	GSH
4.232	3%	0.862	Ins
0.622	13%	0.127	Lac
7.190	2%	1.464	NAA
0.803	11%	0.163	NAAG
0.176	12%	3.6E-02	Scyllo
0.904	7%	0.184	Tau
0.575	16%	0.117	-CrCH2
0.669	3%	0.136	GPC+PCh
7.993	2%	1.627	NAA+NAAG
4.912	1%	1.000	Cr+PCr
6.426	2%	1.308	Glu+Gln
0.712	49%	0.145	Lip13a
0.000	999%	0.000	Lip13b
0.551	44%	0.112	Lip09
6.019	11%	1.225	MM09
0.680	25%	0.138	Lip20
9.085	13%	1.850	MM20
1.923	23%	0.391	MM12
4.307	16%	0.877	MM14
4.849	18%	0.987	MM17
0.712	49%	0.145	Lip13a+Lip13b
6.942	14%	1.413	MM14+Lip13a+L
6.570	9%	1.338	MM09+Lip09
9.765	12%	1.988	MM20+Lip20

DIAGNOSTICS

1 info STARTV 24

Doing Water-Scaling

MISCELLANEOUS OUTPUT

FWHM = 0.045 ppm S/N = 60

Data shift = 0.015 ppm

Ph: 7 deg -11.5 deg/ppm

MIGRAINE WITHOUT AURA-30yo-Female-Post-Checkerboard

LCModel (Version 6.3-0B) Copyright: S.W. Provencher. Ref.: Magn. Reson. Med. 30:672-679 (1993). 31-July-2018 13:55

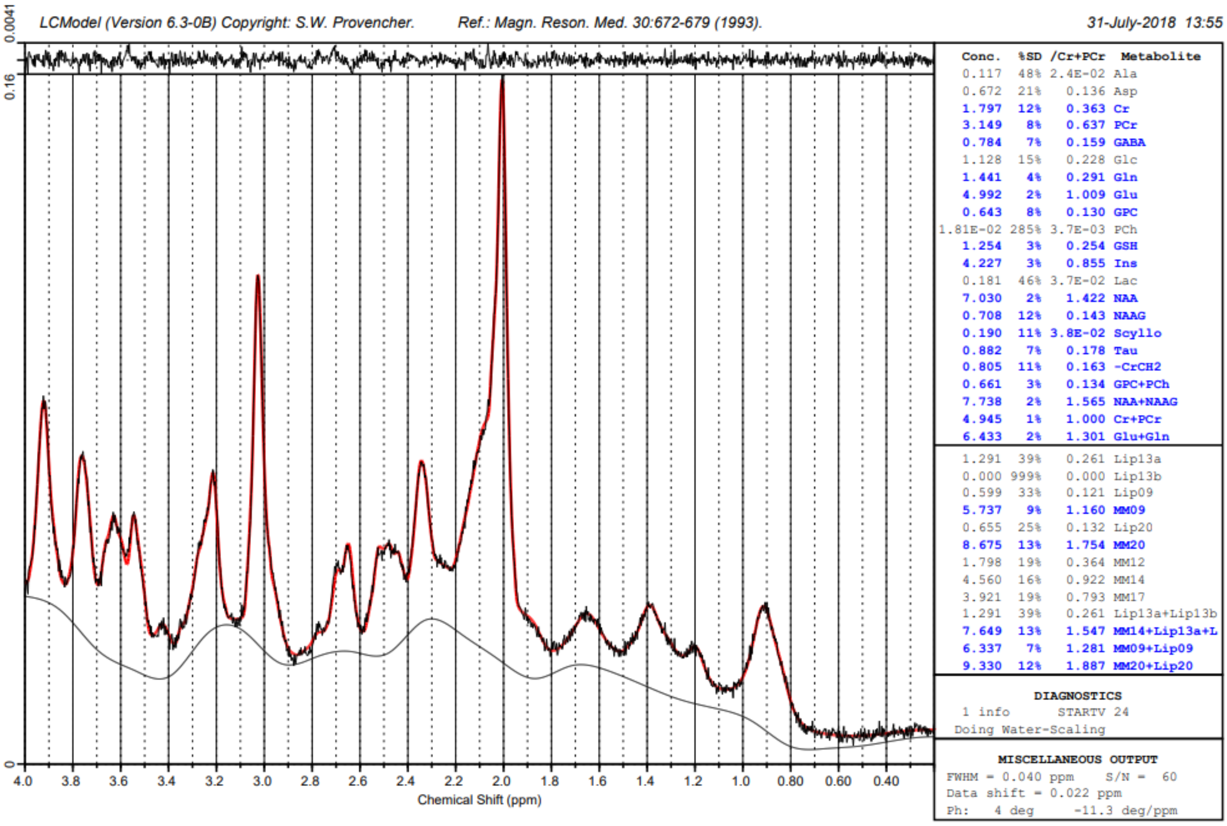

Conc.	%SD	/Cr+PCr	Metabolite
0.117	48%	2.4E-02	Ala
0.672	21%	0.136	Asp
1.797	12%	0.363	Cr
3.149	8%	0.637	PCr
0.784	7%	0.159	GABA
1.128	15%	0.228	Glc
1.441	4%	0.291	Gln
4.992	2%	1.009	Glu
0.643	8%	0.130	GPC
1.81E-02	285%	3.7E-03	PCh
1.254	3%	0.254	GSH
4.227	3%	0.855	Ins
0.181	46%	3.7E-02	Lac
7.030	2%	1.422	NAA
0.708	12%	0.143	NAAG
0.190	11%	3.8E-02	Scyllo
0.882	7%	0.178	Tau
0.805	11%	0.163	-CrCH2
0.661	3%	0.134	GPC+PCh
7.738	2%	1.565	NAA+NAAG
4.945	1%	1.000	Cr+PCr
6.433	2%	1.301	Glu+Gln
1.291	39%	0.261	Lip13a
0.000	999%	0.000	Lip13b
0.599	33%	0.121	Lip09
5.737	9%	1.160	MM09
0.655	25%	0.132	Lip20
8.675	13%	1.754	MM20
1.798	19%	0.364	MM12
4.560	16%	0.922	MM14
3.921	19%	0.793	MM17
1.291	39%	0.261	Lip13a+Lip13b
7.649	13%	1.547	MM14+Lip13a+L
6.337	7%	1.281	MM09+Lip09
9.330	12%	1.887	MM20+Lip20

DIAGNOSTICS

1 info STARTV 24

Doing Water-Scaling

MISCELLANEOUS OUTPUT

FWHM = 0.040 ppm S/N = 60

Data shift = 0.022 ppm

Ph: 4 deg -11.3 deg/ppm

MIGRAINE WITH AURA-28yo-Male-Pre-checkerboard

LCModel (Version 6.3-0B) Copyright: S.W. Provencher.   *Ref.: Magn. Reson. Med. 30:672-679 (1993).*   *31-July-2018 13:56*

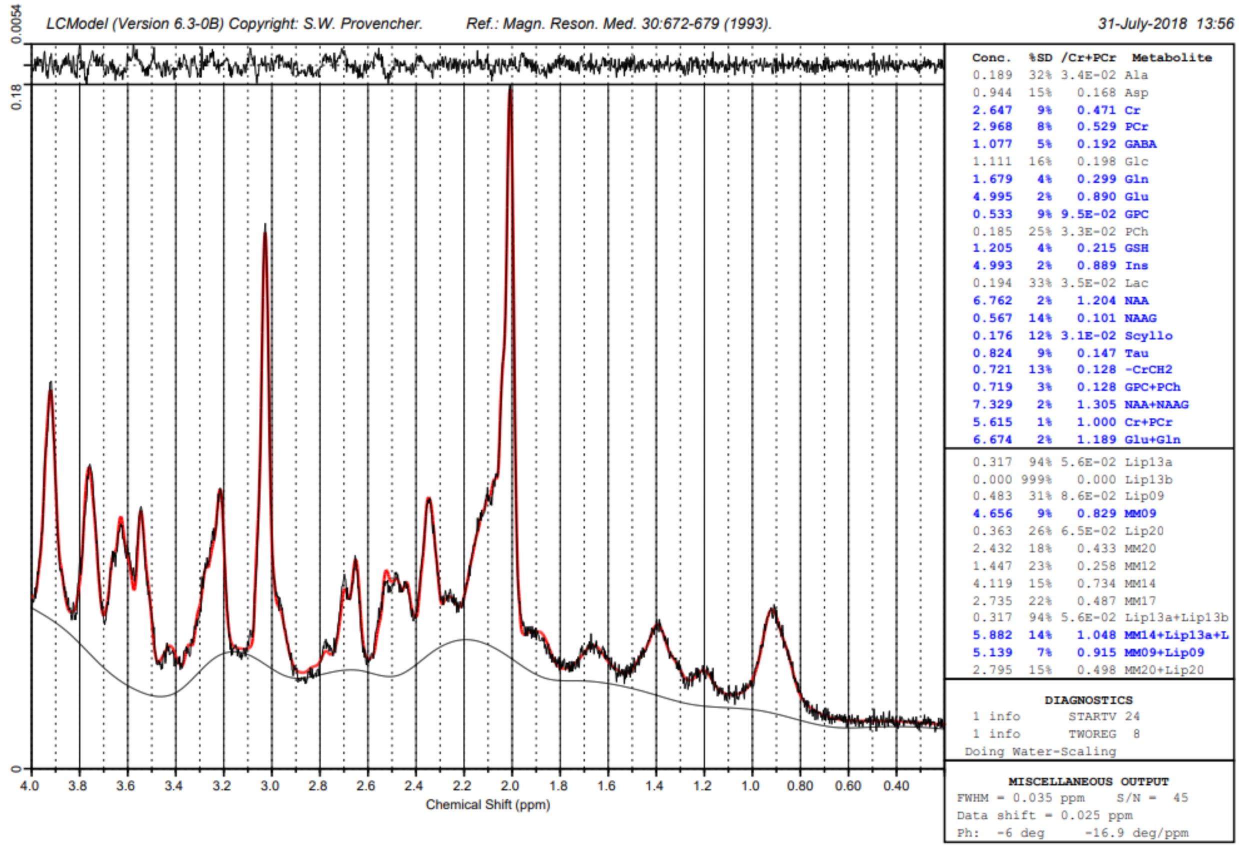

Conc.	%SD	/Cr+PCr	Metabolite
0.189	32%	3.4E-02	Ala
0.944	15%	0.168	Asp
2.647	9%	0.471	Cr
2.968	8%	0.529	PCr
1.077	5%	0.192	GABA
1.111	16%	0.198	Glc
1.679	4%	0.299	Gln
4.995	2%	0.890	Glu
0.533	9%	9.5E-02	GPC
0.185	25%	3.3E-02	PCh
1.205	4%	0.215	GSH
4.993	2%	0.889	Ins
0.194	33%	3.5E-02	Lac
6.762	2%	1.204	NAA
0.567	14%	0.101	NAAG
0.176	12%	3.1E-02	Scyllo
0.824	9%	0.147	Tau
0.721	13%	0.128	-CrCH2
0.719	3%	0.128	GPC+PCh
7.329	2%	1.305	NAA+NAAG
5.615	1%	1.000	Cr+PCr
6.674	2%	1.189	Glu+Gln
0.317	94%	5.6E-02	Lip13a
0.000	999%	0.000	Lip13b
0.483	31%	8.6E-02	Lip09
4.656	9%	0.829	MM09
0.363	26%	6.5E-02	Lip20
2.432	18%	0.433	MM20
1.447	23%	0.258	MM12
4.119	15%	0.734	MM14
2.735	22%	0.487	MM17
0.317	94%	5.6E-02	Lip13a+Lip13b
5.882	14%	1.048	MM14+Lip13a+L
5.139	7%	0.915	MM09+Lip09
2.795	15%	0.498	MM20+Lip20

DIAGNOSTICS

1 info   STARTV 24
1 info   TWOREG 8
Doing Water-Scaling

MISCELLANEOUS OUTPUT

FWHM = 0.035 ppm   S/N = 45
Data shift = 0.025 ppm
Ph: -6 deg   -16.9 deg/ppm

MIGRAINE WITH AURA-28yo-Male-Post-checkerboard

LCModel (Version 6.3-0B) Copyright: S.W. Provencher. *Ref.: Magn. Reson. Med. 30:672-679 (1993).* *31-July-2018 13:55*

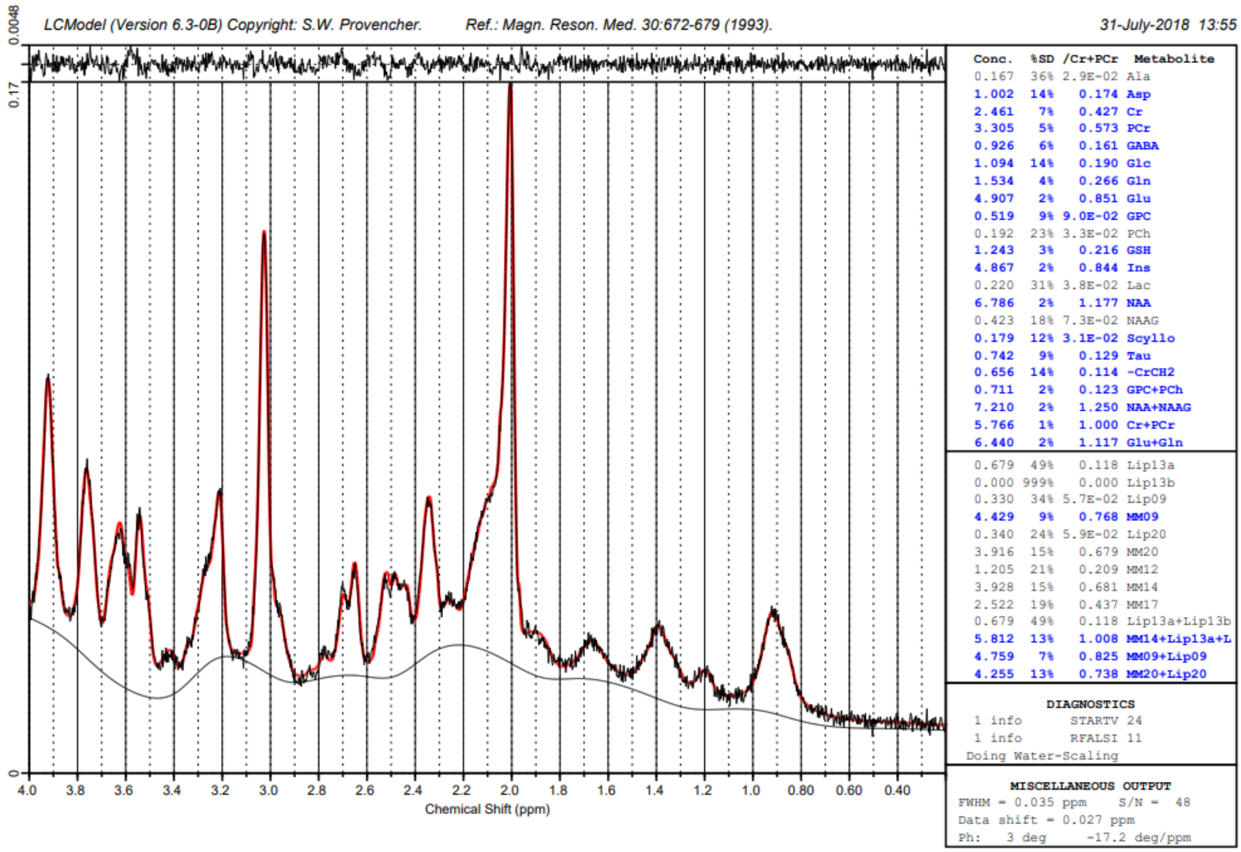

Conc.	%SD	/Cr+PCr	Metabolite
0.167	36%	2.9E-02	Ala
1.002	14%	0.174	Asp
2.461	7%	0.427	Cr
3.305	5%	0.573	PCr
0.926	6%	0.161	GABA
1.094	14%	0.190	Glc
1.534	4%	0.266	Gln
4.907	2%	0.851	Glu
0.519	9%	9.0E-02	GPC
0.192	23%	3.3E-02	PCh
1.243	3%	0.216	GSH
4.867	2%	0.844	Ins
0.220	31%	3.8E-02	Lac
6.786	2%	1.177	NAA
0.423	18%	7.3E-02	NAAG
0.179	12%	3.1E-02	Scyllo
0.742	9%	0.129	Tau
0.656	14%	0.114	-CrCH2
0.711	2%	0.123	GPC+PCh
7.210	2%	1.250	NAA+NAAG
5.766	1%	1.000	Cr+PCr
6.440	2%	1.117	Glu+Gln
0.679	49%	0.118	Lip13a
0.000	999%	0.000	Lip13b
0.330	34%	5.7E-02	Lip09
4.429	9%	0.768	MM09
0.340	24%	5.9E-02	Lip20
3.916	15%	0.679	MM20
1.205	21%	0.209	MM12
3.928	15%	0.681	MM14
2.522	19%	0.437	MM17
0.679	49%	0.118	Lip13a+Lip13b
5.812	13%	1.008	MM14+Lip13a+L
4.759	7%	0.825	MM09+Lip09
4.255	13%	0.738	MM20+Lip20

DIAGNOSTICS

1 info STARTV 24

1 info RFALSI 11

Doing Water-Scaling

MISCELLANEOUS OUTPUT

FWHM = 0.035 ppm S/N = 48

Data shift = 0.027 ppm

Ph: 3 deg -17.2 deg/ppm

CONTROL-24yo-Female-Pre-checkerboard

(2362) Series/Acq=21/1 (2018.05.29 15:05) Pre Checkerboard TR/TE/NS=8500/6/32, 1.500E+01mL (F 023Y, 48kg) MBCIU PROTOCOLS 7T-2017.007 Migraine MRS (University of Melbourne - Brain Institute) _c_32

Data of: Radiology Department, The Royal Melbourne Hospital

LCModel (Version 6.3-0B) Copyright: S.W. Provencher. Ref.: Magn. Reson. Med. 30:672-679 (1993). *31-July-2018 13:56*

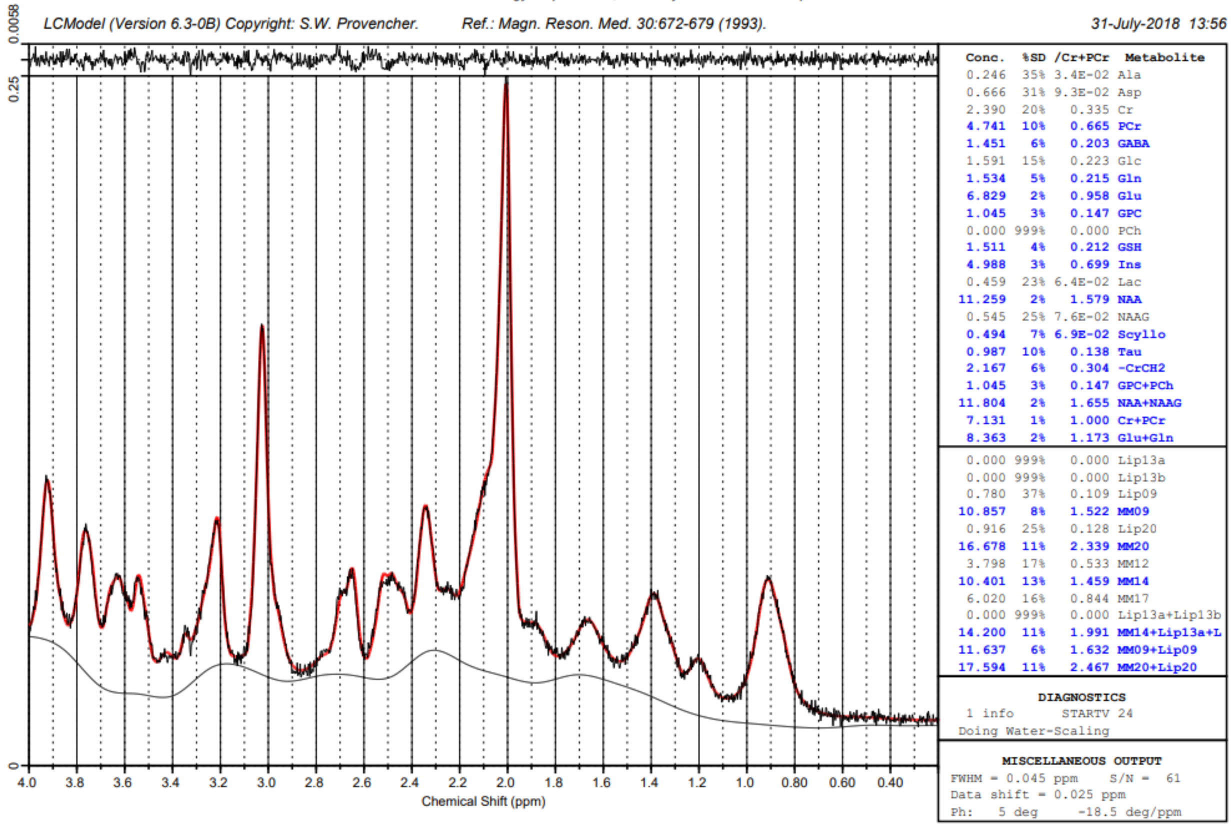

Conc.	%SD	/Cr+PCr	Metabolite
0.246	35%	3.4E-02	Ala
0.666	31%	9.3E-02	Asp
2.390	20%	0.335	Cr
4.741	10%	0.665	PCr
1.451	6%	0.203	GABA
1.591	15%	0.223	Glc
1.534	5%	0.215	Gln
6.829	2%	0.958	Glu
1.045	3%	0.147	GPC
0.000	999%	0.000	PCh
1.511	4%	0.212	GSH
4.988	3%	0.699	Ins
0.459	23%	6.4E-02	Lac
11.259	2%	1.579	NAA
0.545	25%	7.6E-02	NAAG
0.494	7%	6.9E-02	Scyllo
0.987	10%	0.138	Tau
2.167	6%	0.304	-CrCH2
1.045	3%	0.147	GPC+PCh
11.804	2%	1.655	NAA+NAAG
7.131	1%	1.000	Cr+PCr
8.363	2%	1.173	Glu+Gln
0.000	999%	0.000	Lip13a
0.000	999%	0.000	Lip13b
0.780	37%	0.109	Lip09
10.857	8%	1.522	MM09
0.916	25%	0.128	Lip20
16.678	11%	2.339	MM20
3.798	17%	0.533	MM12
10.401	13%	1.459	MM14
6.020	16%	0.844	MM17
0.000	999%	0.000	Lip13a+Lip13b
14.200	11%	1.991	MM14+Lip13a+L
11.637	6%	1.632	MM09+Lip09
17.594	11%	2.467	MM20+Lip20

DIAGNOSTICS

1 info STARTV 24

Doing Water-Scaling

MISCELLANEOUS OUTPUT

FWHM = 0.045 ppm S/N = 61

Data shift = 0.025 ppm

Ph: 5 deg -18.5 deg/ppm

CONTROL-24yo-Female-Post-checkerboard

LCModel (Version 6.3-0B) Copyright: S.W. Provencher. *Ref.: Magn. Reson. Med. 30:672-679 (1993).* *31-July-2018 13:56*

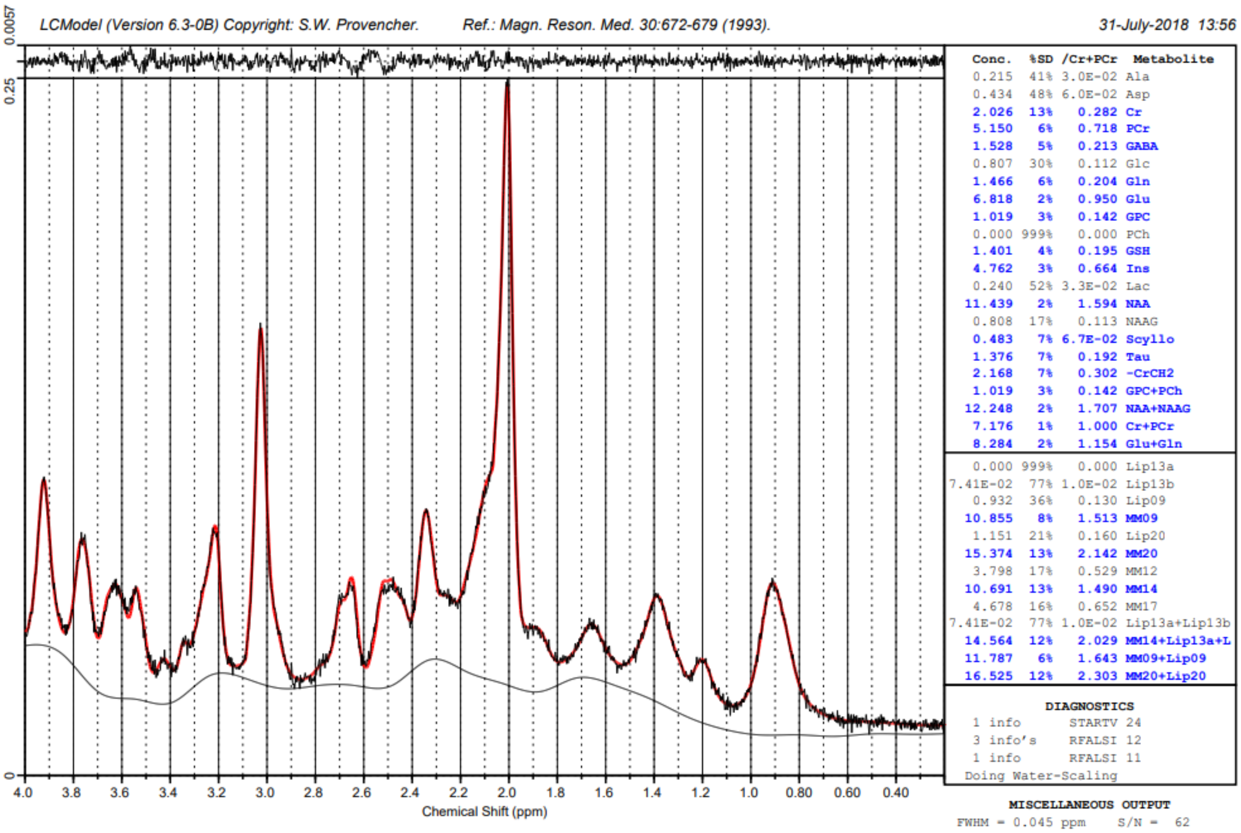

Conc.	%SD	/Cr+PCr	Metabolite
0.215	41%	3.0E-02	Ala
0.434	48%	6.0E-02	Asp
2.026	13%	0.282	Cr
5.150	6%	0.718	PCr
1.528	5%	0.213	GABA
0.807	30%	0.112	Glc
1.466	6%	0.204	Gln
6.818	2%	0.950	Glu
1.019	3%	0.142	GPC
0.000	999%	0.000	PCh
1.401	4%	0.195	GSH
4.762	3%	0.664	Ins
0.240	52%	3.3E-02	Lac
11.439	2%	1.594	NAA
0.808	17%	0.113	NAAG
0.483	7%	6.7E-02	Scyllo
1.376	7%	0.192	Tau
2.168	7%	0.302	-CrCH2
1.019	3%	0.142	GPC+PCh
12.248	2%	1.707	NAA+NAAG
7.176	1%	1.000	Cr+PCr
8.284	2%	1.154	Glu+Gln
0.000	999%	0.000	Lip13a
7.41E-02	77%	1.0E-02	Lip13b
0.932	36%	0.130	Lip09
10.855	8%	1.513	MM09
1.151	21%	0.160	Lip20
15.374	13%	2.142	MM20
3.798	17%	0.529	MM12
10.691	13%	1.490	MM14
4.678	16%	0.652	MM17
7.41E-02	77%	1.0E-02	Lip13a+Lip13b
14.564	12%	2.029	MM14+Lip13a+L
11.787	6%	1.643	MM09+Lip09
16.525	12%	2.303	MM20+Lip20

DIAGNOSTICS

1 info	STARTV 24
3 info's	RFALSI 12
1 info	RFALSI 11

Doing Water-Scaling

MISCELLANEOUS OUTPUT

FWHM = 0.045 ppm S/N = 62

Data shift = 0.030 ppm

MIGRAINE WITHOUT AURA-26yo-Female-Pre-checkerboard

LCModel (Version 6.3-0B) Copyright: S.W. Provencher. Ref.: Magn. Reson. Med. 30:672-679 (1993). 31-July-2018 13:56

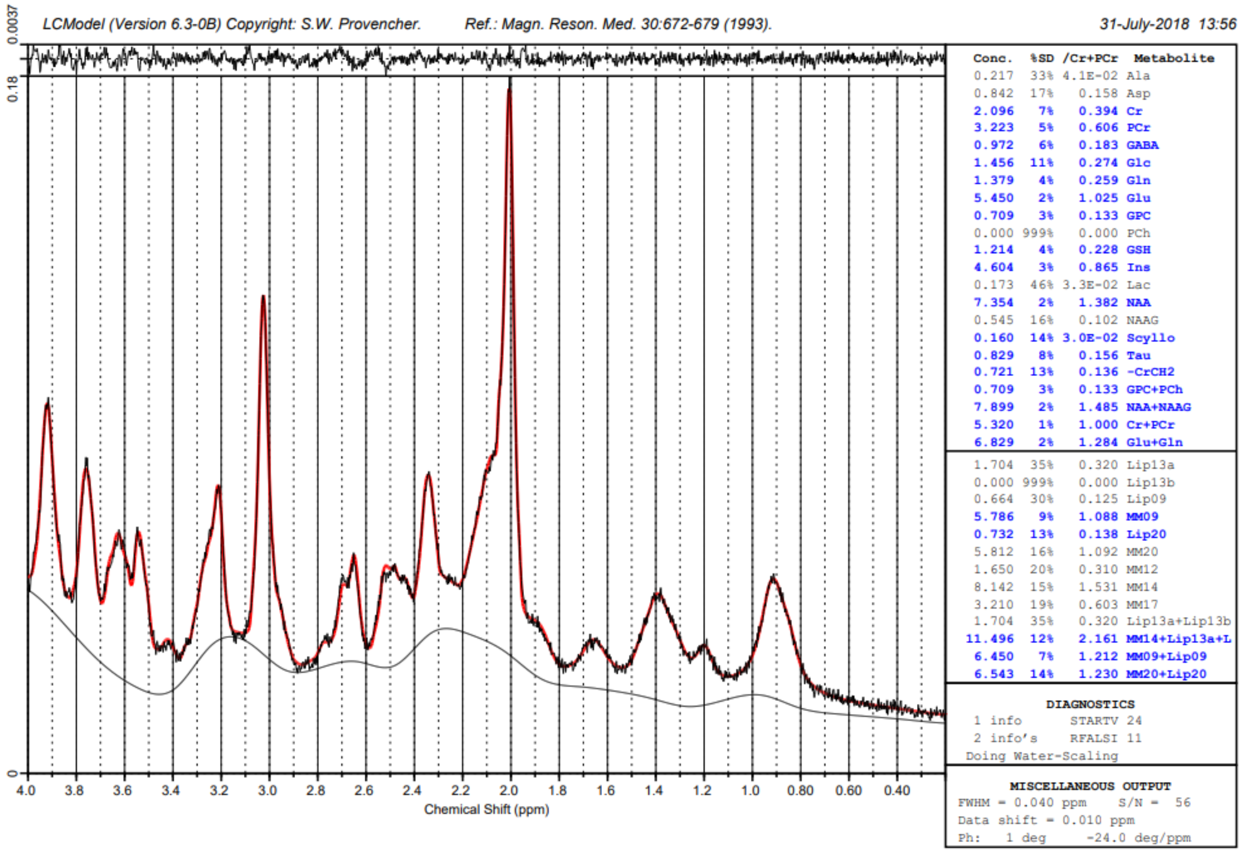

Conc.	%SD	/Cr+PCr	Metabolite
0.217	33%	4.1E-02	Ala
0.842	17%	0.158	Asp
2.096	7%	0.394	Cr
3.223	5%	0.606	PCr
0.972	6%	0.183	GABA
1.456	11%	0.274	Glc
1.379	4%	0.259	Gln
5.450	2%	1.025	Glu
0.709	3%	0.133	GPC
0.000	999%	0.000	PCh
1.214	4%	0.228	GSH
4.604	3%	0.865	Ins
0.173	46%	3.3E-02	Lac
7.354	2%	1.382	NAA
0.545	16%	0.102	NAAG
0.160	14%	3.0E-02	Scyllo
0.829	8%	0.156	Tau
0.721	13%	0.136	-CrCH2
0.709	3%	0.133	GPC+PCh
7.899	2%	1.485	NAA+NAAG
5.320	1%	1.000	Cr+PCr
6.829	2%	1.284	Glu+Gln
1.704	35%	0.320	Lip13a
0.000	999%	0.000	Lip13b
0.664	30%	0.125	Lip09
5.786	9%	1.088	MM09
0.732	13%	0.138	Lip20
5.812	16%	1.092	MM20
1.650	20%	0.310	MM12
8.142	15%	1.531	MM14
3.210	19%	0.603	MM17
1.704	35%	0.320	Lip13a+Lip13b
11.496	12%	2.161	MM14+Lip13a+L
6.450	7%	1.212	MM09+Lip09
6.543	14%	1.230	MM20+Lip20

DIAGNOSTICS

1 info STARTV 24
2 info's RFALSI 11
Doing Water-Scaling

MISCELLANEOUS OUTPUT

FWHM = 0.040 ppm S/N = 56
Data shift = 0.010 ppm
Ph: 1 deg -24.0 deg/ppm

MIGRAINE WITHOUT AURA-26yo-Female-Post-checkerboard

LCModel (Version 6.3-0B) Copyright: S.W. Provencher. *Ref.: Magn. Reson. Med. 30:672-679 (1993).* *31-July-2018 13:56*

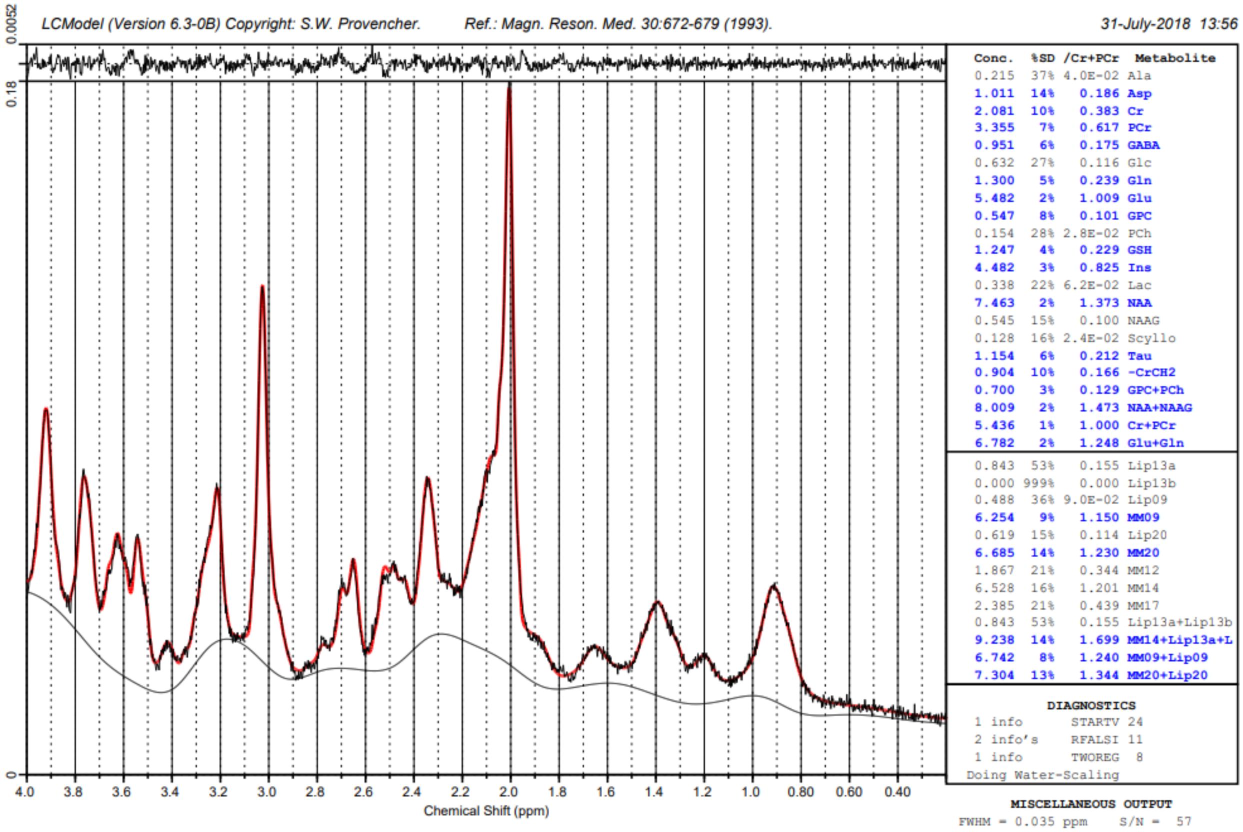

Conc.	%SD	/Cr+PCr	Metabolite
0.215	37%	4.0E-02	Ala
1.011	14%	0.186	Asp
2.081	10%	0.383	Cr
3.355	7%	0.617	PCr
0.951	6%	0.175	GABA
0.632	27%	0.116	Glc
1.300	5%	0.239	Gln
5.482	2%	1.009	Glu
0.547	8%	0.101	GPC
0.154	28%	2.8E-02	PCh
1.247	4%	0.229	GSH
4.482	3%	0.825	Ins
0.338	22%	6.2E-02	Lac
7.463	2%	1.373	NAA
0.545	15%	0.100	NAAG
0.128	16%	2.4E-02	Scyllo
1.154	6%	0.212	Tau
0.904	10%	0.166	-CrCH2
0.700	3%	0.129	GPC+PCh
8.009	2%	1.473	NAA+NAAG
5.436	1%	1.000	Cr+PCr
6.782	2%	1.248	Glu+Gln
0.843	53%	0.155	Lip13a
0.000	999%	0.000	Lip13b
0.488	36%	9.0E-02	Lip09
6.254	9%	1.150	MM09
0.619	15%	0.114	Lip20
6.685	14%	1.230	MM20
1.867	21%	0.344	MM12
6.528	16%	1.201	MM14
2.385	21%	0.439	MM17
0.843	53%	0.155	Lip13a+Lip13b
9.238	14%	1.699	MM14+Lip13a+L
6.742	8%	1.240	MM09+Lip09
7.304	13%	1.344	MM20+Lip20

DIAGNOSTICS

1 info	STARTV 24
2 info's	RFALSI 11
1 info	TWOREG 8

Doing Water-Scaling

MISCELLANEOUS OUTPUT

FWHM = 0.035 ppm S/N = 57

Data shift = 0.020 ppm

MIGRAINE WITHOUT AURA-40yo-Female-Pre-checkerboard

LCModel (Version 6.3-0B) Copyright: S.W. Provencher. *Ref.: Magn. Reson. Med. 30:672-679 (1993).* *31-July-2018 13:57*

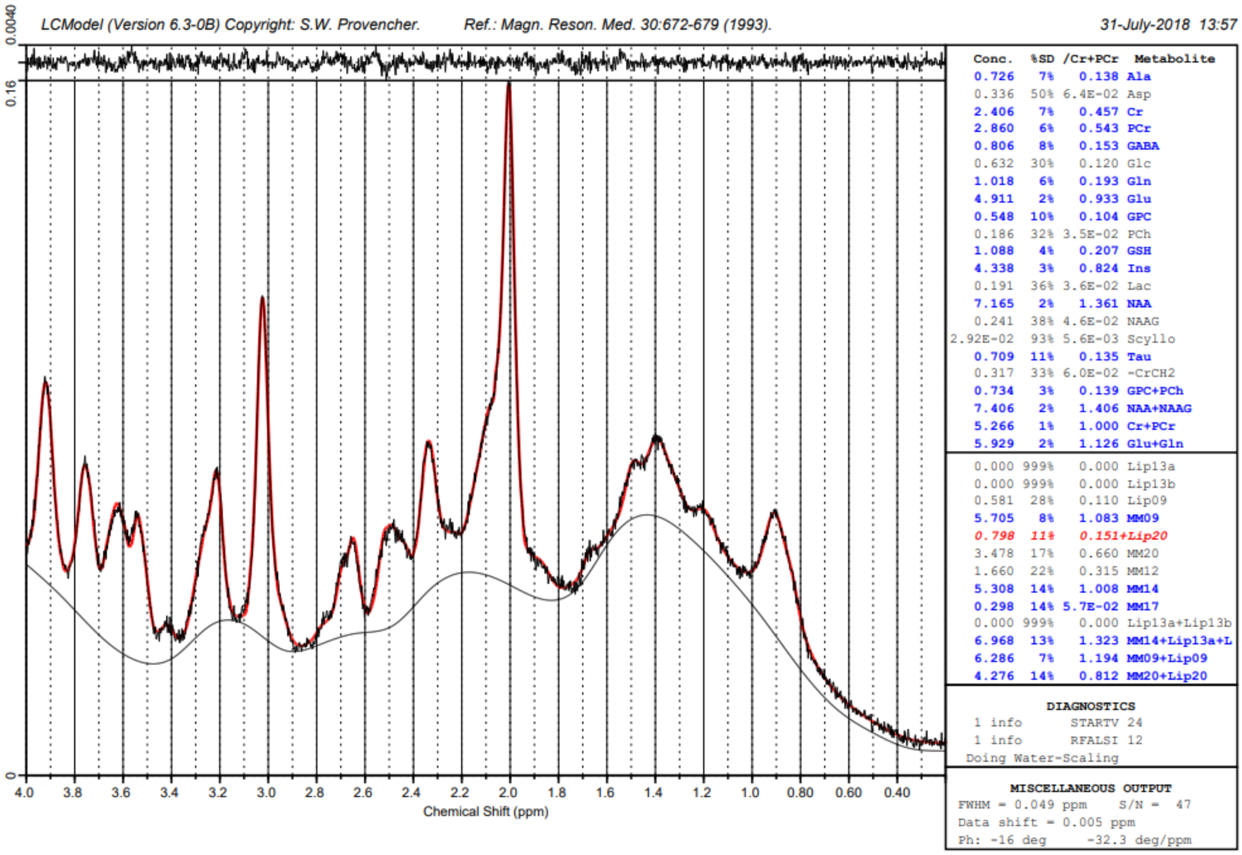

Conc.	%SD	/Cr+PCr	Metabolite
0.726	7%	0.138	Ala
0.336	50%	6.4E-02	Asp
2.406	7%	0.457	Cr
2.860	6%	0.543	PCr
0.806	8%	0.153	GABA
0.632	30%	0.120	Glc
1.018	6%	0.193	Gln
4.911	2%	0.933	Glu
0.548	10%	0.104	GPC
0.186	32%	3.5E-02	PCh
1.088	4%	0.207	GSH
4.338	3%	0.824	Ins
0.191	36%	3.6E-02	Lac
7.165	2%	1.361	NAA
0.241	38%	4.6E-02	NAAG
2.92E-02	93%	5.6E-03	Scyllo
0.709	11%	0.135	Tau
0.317	33%	6.0E-02	-CrCH2
0.734	3%	0.139	GPC+PCh
7.406	2%	1.406	NAA+NAAG
5.266	1%	1.000	Cr+PCr
5.929	2%	1.126	Glu+Gln
0.000	999%	0.000	Lip13a
0.000	999%	0.000	Lip13b
0.581	28%	0.110	Lip09
5.705	8%	1.083	MM09
0.798	*11%*	*0.151*	*+Lip20*
3.478	17%	0.660	MM20
1.660	22%	0.315	MM12
5.308	14%	1.008	MM14
0.298	14%	5.7E-02	MM17
0.000	999%	0.000	Lip13a+Lip13b
6.968	13%	1.323	MM14+Lip13a+L
6.286	7%	1.194	MM09+Lip09
4.276	14%	0.812	MM20+Lip20

DIAGNOSTICS

1 info STARTV 24
1 info RFALSI 12
Doing Water-Scaling

MISCELLANEOUS OUTPUT

FWHM = 0.049 ppm S/N = 47
Data shift = 0.005 ppm
Ph: -16 deg -32.3 deg/ppm

MIGRAINE WITHOUT AURA-40yo-Female-Post-checkerboard

LCModel (Version 6.3-0B) Copyright: S.W. Provencher. *Ref.: Magn. Reson. Med. 30:672-679 (1993).* *31-July-2018 13:57*

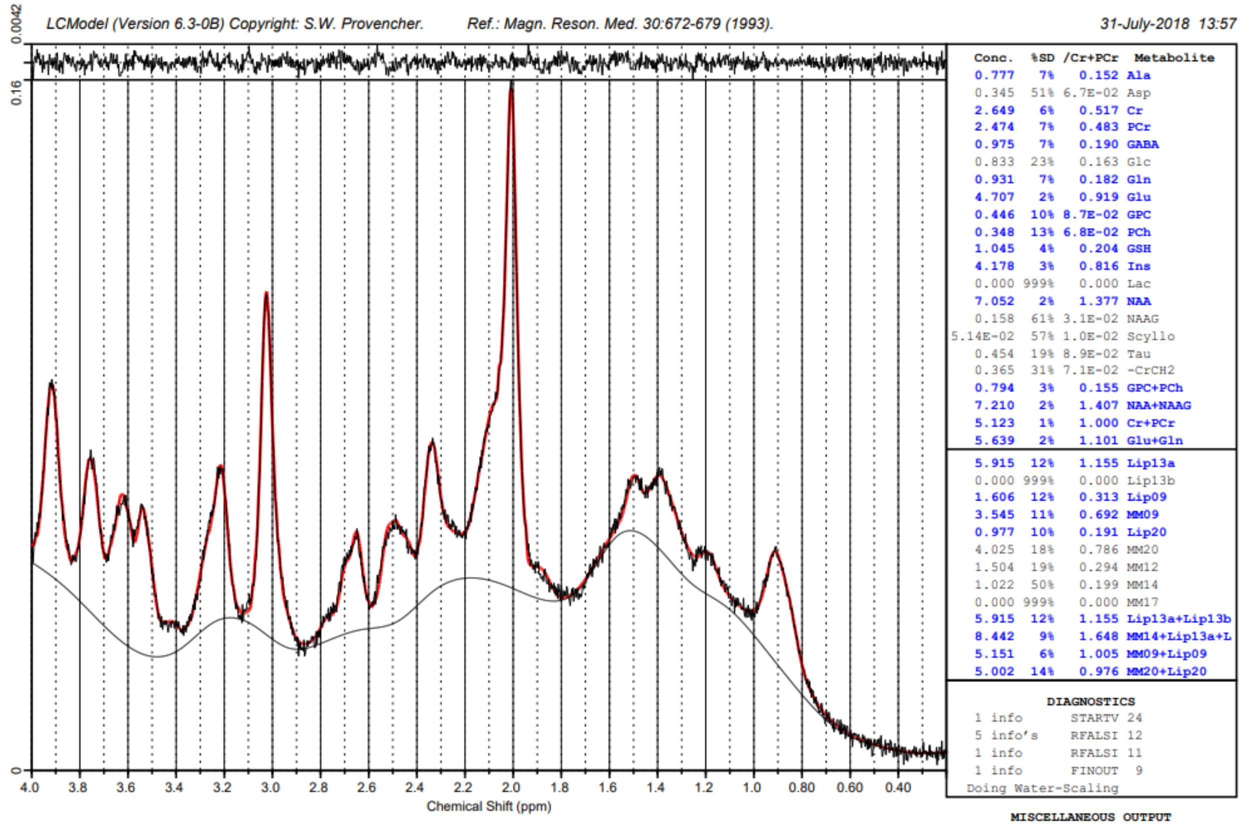

Conc.	%SD	/Cr+PCr	Metabolite
0.777	7%	0.152	Ala
0.345	51%	6.7E-02	Asp
2.649	6%	0.517	Cr
2.474	7%	0.483	PCr
0.975	7%	0.190	GABA
0.833	23%	0.163	Glc
0.931	7%	0.182	Gln
4.707	2%	0.919	Glu
0.446	10%	8.7E-02	GPC
0.348	13%	6.8E-02	PCh
1.045	4%	0.204	GSH
4.178	3%	0.816	Ins
0.000	999%	0.000	Lac
7.052	2%	1.377	NAA
0.158	61%	3.1E-02	NAAG
5.14E-02	57%	1.0E-02	Scyllo
0.454	19%	8.9E-02	Tau
0.365	31%	7.1E-02	-CrCH2
0.794	3%	0.155	GPC+PCh
7.210	2%	1.407	NAA+NAAG
5.123	1%	1.000	Cr+PCr
5.639	2%	1.101	Glu+Gln
5.915	12%	1.155	Lip13a
0.000	999%	0.000	Lip13b
1.606	12%	0.313	Lip09
3.545	11%	0.692	MM09
0.977	10%	0.191	Lip20
4.025	18%	0.786	MM20
1.504	19%	0.294	MM12
1.022	50%	0.199	MM14
0.000	999%	0.000	MM17
5.915	12%	1.155	Lip13a+Lip13b
8.442	9%	1.648	MM14+Lip13a+L
5.151	6%	1.005	MM09+Lip09
5.002	14%	0.976	MM20+Lip20

DIAGNOSTICS

1 info	STARTV 24
5 info's	RFALSI 12
1 info	RFALSI 11
1 info	FINOUT 9

Doing Water-Scaling

MISCELLANEOUS OUTPUT

FWHM = 0.049 ppm S/N = 42

MIGRAINE WITH AURA-29yo-Female-Pre-checkerboard

LCModel (Version 6.3-0B) Copyright: S.W. Provencher. *Ref.: Magn. Reson. Med. 30:672-679 (1993).* *31-July-2018 13:57*

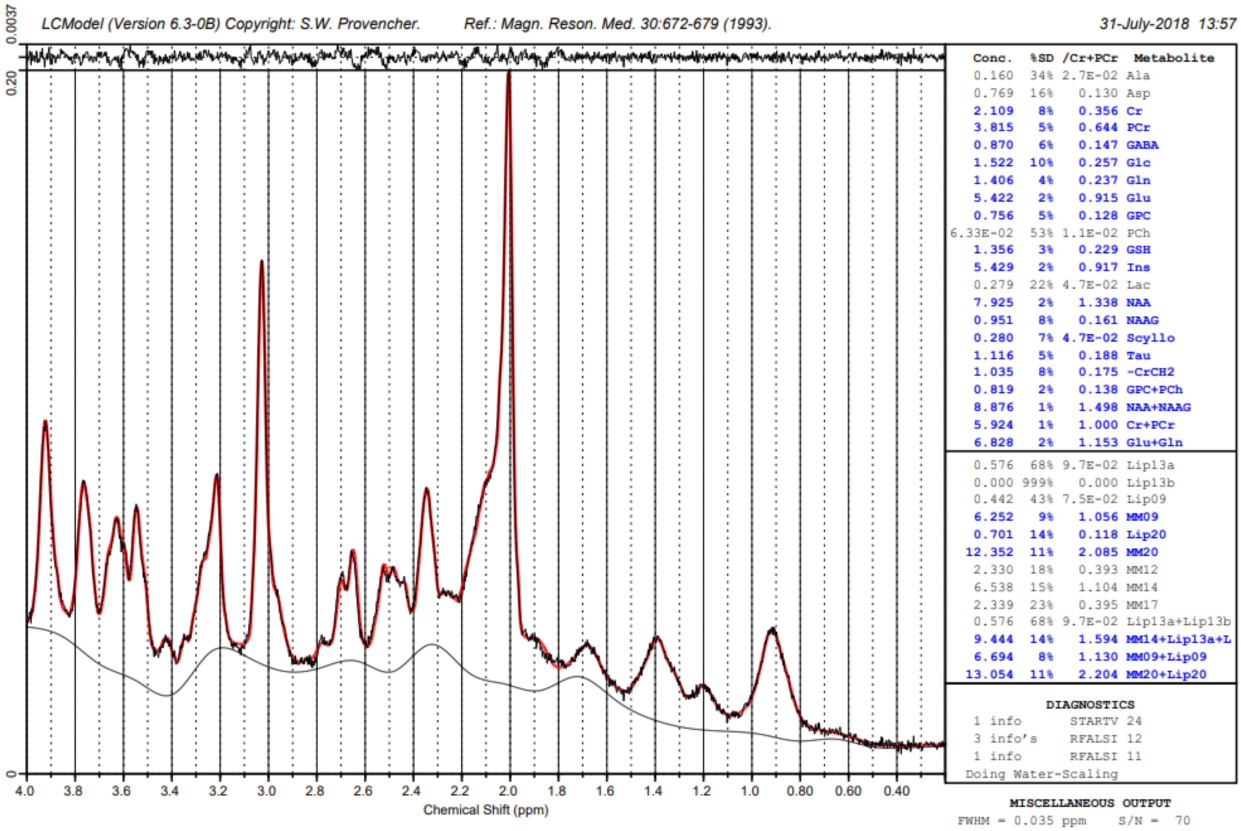

Conc.	%SD	/Cr+PCr	Metabolite
0.160	34%	2.7E-02	Ala
0.769	16%	0.130	Asp
**2.109**	**8%**	**0.356**	**Cr**
**3.815**	**5%**	**0.644**	**PCr**
**0.870**	**6%**	**0.147**	**GABA**
**1.522**	**10%**	**0.257**	**Glc**
**1.406**	**4%**	**0.237**	**Gln**
**5.422**	**2%**	**0.915**	**Glu**
**0.756**	**5%**	**0.128**	**GPC**
6.33E-02	53%	1.1E-02	PCh
**1.356**	**3%**	**0.229**	**GSH**
**5.429**	**2%**	**0.917**	**Ins**
0.279	22%	4.7E-02	Lac
**7.925**	**2%**	**1.338**	**NAA**
**0.951**	**8%**	**0.161**	**NAAG**
**0.280**	**7%**	**4.7E-02**	**Scyllo**
**1.116**	**5%**	**0.188**	**Tau**
**1.035**	**8%**	**0.175**	**-CrCH2**
**0.819**	**2%**	**0.138**	**GPC+PCh**
**8.876**	**1%**	**1.498**	**NAA+NAAG**
**5.924**	**1%**	**1.000**	**Cr+PCr**
**6.828**	**2%**	**1.153**	**Glu+Gln**
0.576	68%	9.7E-02	Lip13a
0.000	999%	0.000	Lip13b
0.442	43%	7.5E-02	Lip09
**6.252**	**9%**	**1.056**	**MM09**
**0.701**	**14%**	**0.118**	**Lip20**
**12.352**	**11%**	**2.085**	**MM20**
2.330	18%	0.393	MM12
6.538	15%	1.104	MM14
2.339	23%	0.395	MM17
0.576	68%	9.7E-02	Lip13a+Lip13b
**9.444**	**14%**	**1.594**	**MM14+Lip13a+L**
**6.694**	**8%**	**1.130**	**MM09+Lip09**
**13.054**	**11%**	**2.204**	**MM20+Lip20**

DIAGNOSTICS

1 info	STARTV	24
3 info's	RFALSI	12
1 info	RFALSI	11

Doing Water-Scaling

MISCELLANEOUS OUTPUT

FWHM = 0.035 ppm S/N = 70
Data shift = 0.022 ppm

MIGRAINE WITH AURA-29yo-Female-Post-checkerboard

LCModel (Version 6.3-0B) Copyright: S.W. Provencher. Ref.: Magn. Reson. Med. 30:672-679 (1993). 31-July-2018 13:57

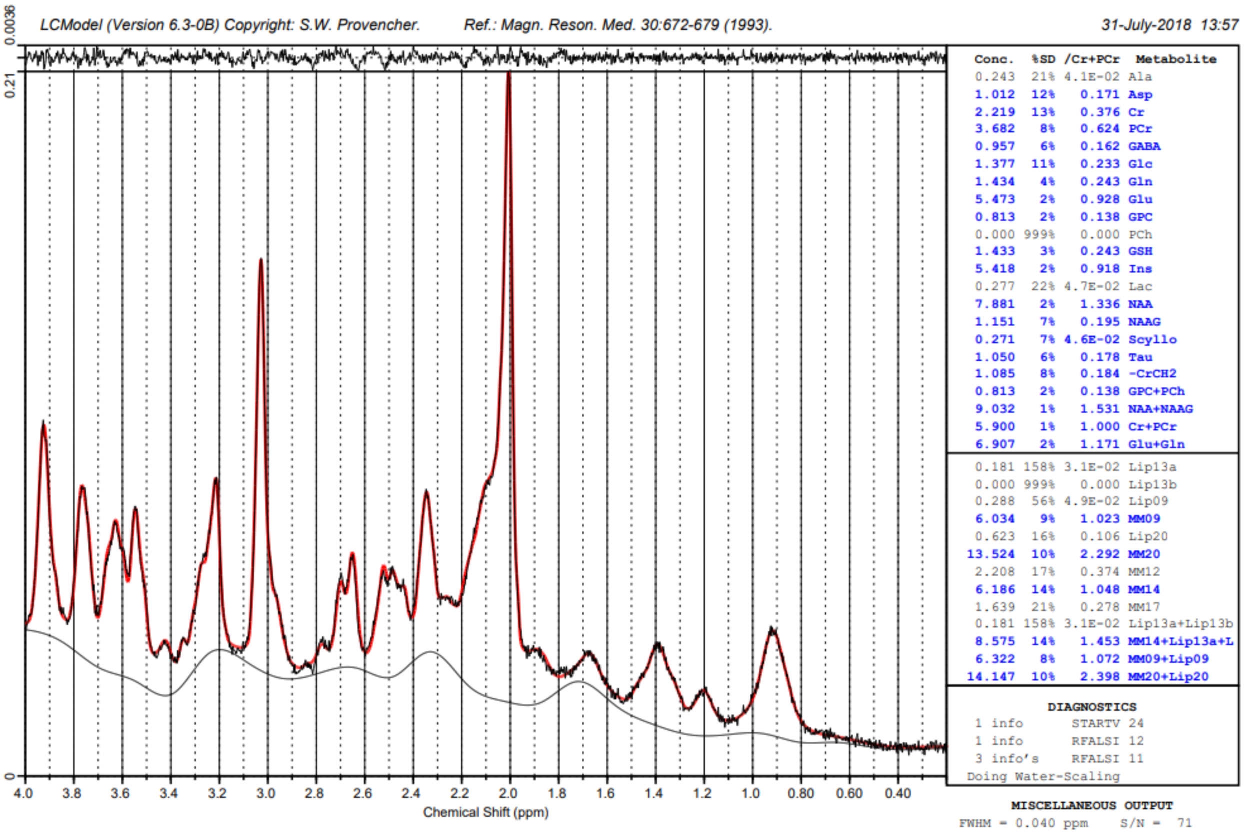

Conc.	%SD	/Cr+PCr	Metabolite
0.243	21%	4.1E-02	Ala
1.012	12%	0.171	Asp
2.219	13%	0.376	Cr
3.682	8%	0.624	PCr
0.957	6%	0.162	GABA
1.377	11%	0.233	Glc
1.434	4%	0.243	Gln
5.473	2%	0.928	Glu
0.813	2%	0.138	GPC
0.000	999%	0.000	PCh
1.433	3%	0.243	GSH
5.418	2%	0.918	Ins
0.277	22%	4.7E-02	Lac
7.881	2%	1.336	NAA
1.151	7%	0.195	NAAG
0.271	7%	4.6E-02	Scyllo
1.050	6%	0.178	Tau
1.085	8%	0.184	-CrCH2
0.813	2%	0.138	GPC+PCh
9.032	1%	1.531	NAA+NAAG
5.900	1%	1.000	Cr+PCr
6.907	2%	1.171	Glu+Gln
0.181	158%	3.1E-02	Lip13a
0.000	999%	0.000	Lip13b
0.288	56%	4.9E-02	Lip09
6.034	9%	1.023	MM09
0.623	16%	0.106	Lip20
13.524	10%	2.292	MM20
2.208	17%	0.374	MM12
6.186	14%	1.048	MM14
1.639	21%	0.278	MM17
0.181	158%	3.1E-02	Lip13a+Lip13b
8.575	14%	1.453	MM14+Lip13a+L
6.322	8%	1.072	MM09+Lip09
14.147	10%	2.398	MM20+Lip20

DIAGNOSTICS

1 info STARTV 24
1 info RFALSI 12
3 info's RFALSI 11
Doing Water-Scaling

MISCELLANEOUS OUTPUT

FWHM = 0.040 ppm S/N = 71
Data shift = 0.030 ppm

MIGRAINE WITHOUT AURA-24yo-Female-Pre-checkerboard

LCModel (Version 6.3-0B) Copyright: S.W. Provencher. *Ref.: Magn. Reson. Med. 30:672-679 (1993).* *31-July-2018 13:58*

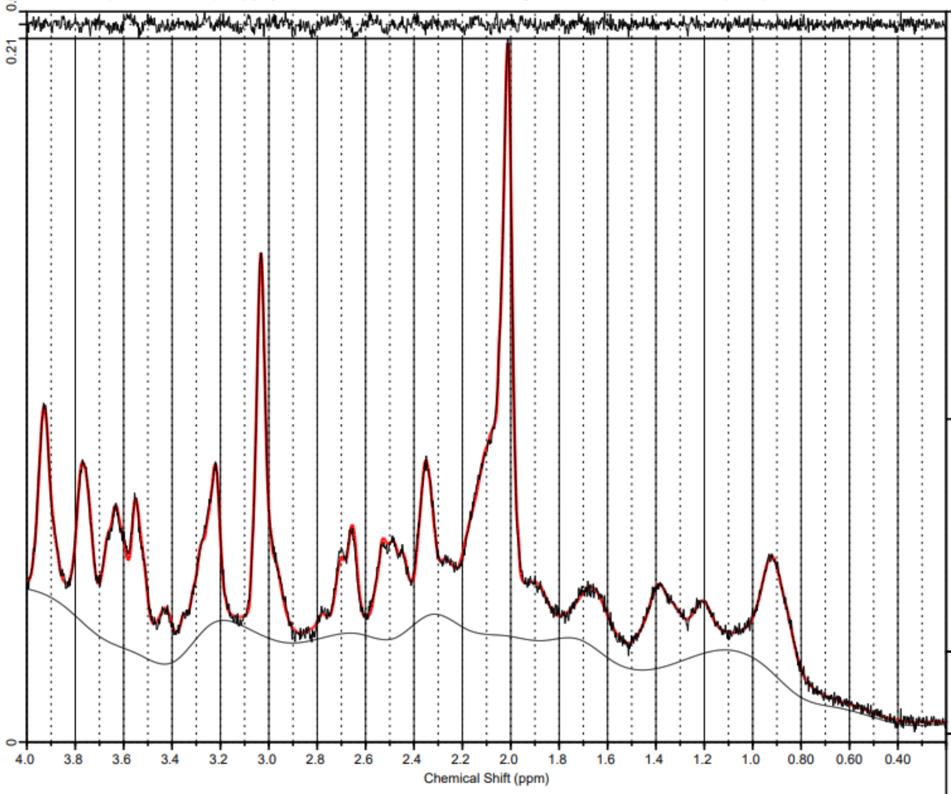

Conc.	%SD	/Cr+PCr	Metabolite
0.105	57%	1.8E-02	Ala
0.929	16%	0.159	Asp
1.819	13%	0.311	Cr
4.037	6%	0.689	PCr
1.051	6%	0.179	GABA
1.415	13%	0.242	Glc
1.387	5%	0.237	Gln
5.666	2%	0.968	Glu
0.708	6%	0.121	GPC
7.02E-02	55%	1.2E-02	PCh
1.368	4%	0.234	GSH
4.968	3%	0.849	Ins
0.288	27%	4.9E-02	Lac
8.120	2%	1.387	NAA
0.623	15%	0.106	NAAG
0.238	9%	4.1E-02	Scyllo
1.123	6%	0.192	Tau
1.233	8%	0.211	-CrCH2
0.778	3%	0.133	GPC+PCh
8.743	2%	1.493	NAA+NAAG
5.855	1%	1.000	Cr+PCr
7.053	2%	1.205	Glu+Gln
0.843	56%	0.144	Lip13a
0.000	999%	0.000	Lip13b
0.611	37%	0.104	Lip09
6.799	9%	1.161	MM09
0.596	20%	0.102	Lip20
11.714	12%	2.001	MM20
2.564	18%	0.438	MM12
6.232	15%	1.064	MM14
6.779	16%	1.158	MM17
0.843	56%	0.144	Lip13a+Lip13b
9.639	13%	1.646	MM14+Lip13a+L
7.410	7%	1.266	MM09+Lip09
12.309	11%	2.102	MM20+Lip20

DIAGNOSTICS

1 info STARTV 24
2 info's RFALSI 12
Doing Water-Scaling

MISCELLANEOUS OUTPUT

FWHM = 0.040 ppm S/N = 60
Data shift = 0.035 ppm
Ph: 8 deg -24.8 deg/ppm

MIGRAINE WITHOUT AURA-24yo-Female-Post-checkerboard

LCModel (Version 6.3-0B) Copyright: S.W. Provencher. *Ref.: Magn. Reson. Med. 30:672-679 (1993).* *31-July-2018 13:58*

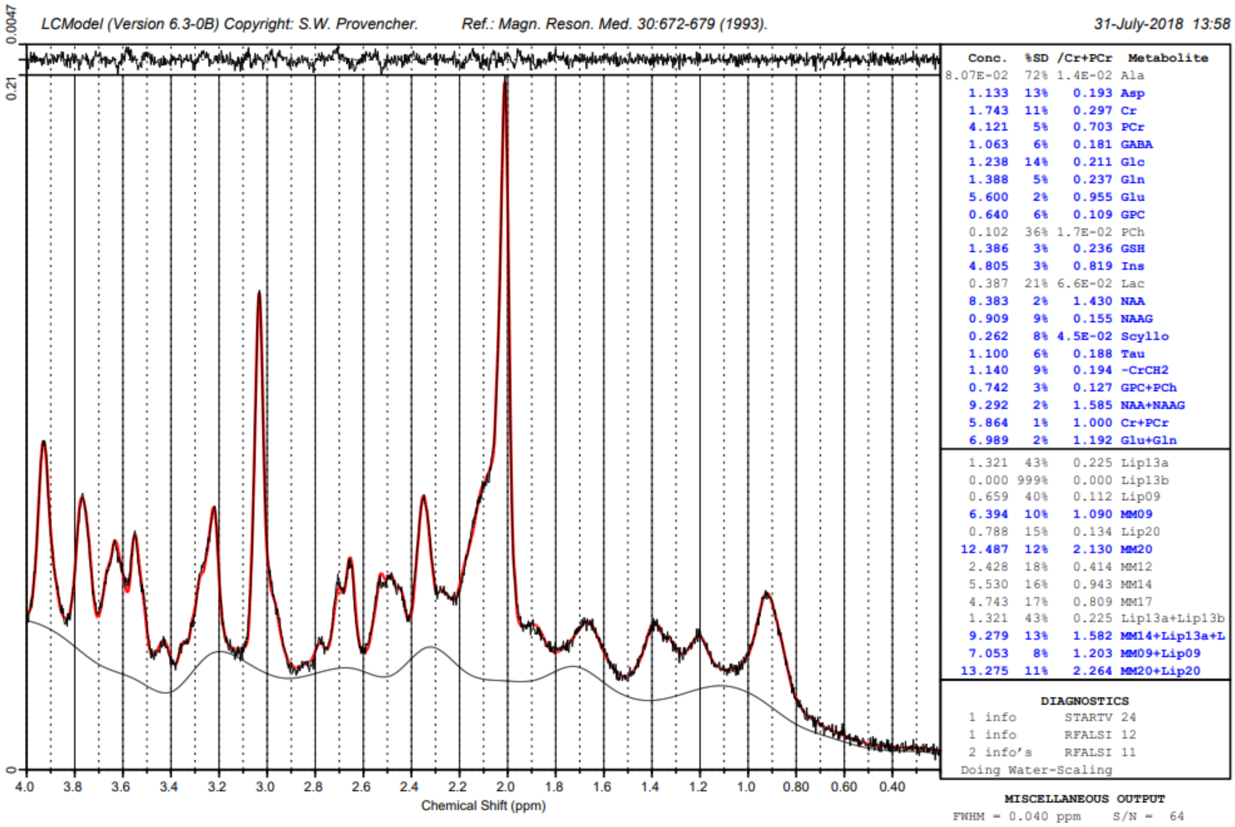

Conc.	%SD	/Cr+PCr	Metabolite
8.07E-02	72%	1.4E-02	Ala
1.133	13%	0.193	Asp
1.743	11%	0.297	Cr
4.121	5%	0.703	PCr
1.063	6%	0.181	GABA
1.238	14%	0.211	Glc
1.388	5%	0.237	Gln
5.600	2%	0.955	Glu
0.640	6%	0.109	GPC
0.102	36%	1.7E-02	PCh
1.386	3%	0.236	GSH
4.805	3%	0.819	Ins
0.387	21%	6.6E-02	Lac
8.383	2%	1.430	NAA
0.909	9%	0.155	NAAG
0.262	8%	4.5E-02	Scyllo
1.100	6%	0.188	Tau
1.140	9%	0.194	-CrCH2
0.742	3%	0.127	GPC+PCh
9.292	2%	1.585	NAA+NAAG
5.864	1%	1.000	Cr+PCr
6.989	2%	1.192	Glu+Gln
1.321	43%	0.225	Lip13a
0.000	999%	0.000	Lip13b
0.659	40%	0.112	Lip09
6.394	10%	1.090	MM09
0.788	15%	0.134	Lip20
12.487	12%	2.130	MM20
2.428	18%	0.414	MM12
5.530	16%	0.943	MM14
4.743	17%	0.809	MM17
1.321	43%	0.225	Lip13a+Lip13b
9.279	13%	1.582	MM14+Lip13a+L
7.053	8%	1.203	MM09+Lip09
13.275	11%	2.264	MM20+Lip20

DIAGNOSTICS

1 info STARTV 24
1 info RFALSI 12
2 info's RFALSI 11
Doing Water-Scaling

MISCELLANEOUS OUTPUT

FWHM = 0.040 ppm S/N = 64
Data shift = 0.037 ppm

MIGRAINE WITHOUT AURA-35yo-Male-Pre-Checkerboard

LCModel (Version 6.3-0B) Copyright: S.W. Provencher. *Ref.: Magn. Reson. Med. 30:672-679 (1993).* *31-July-2018 13:58*

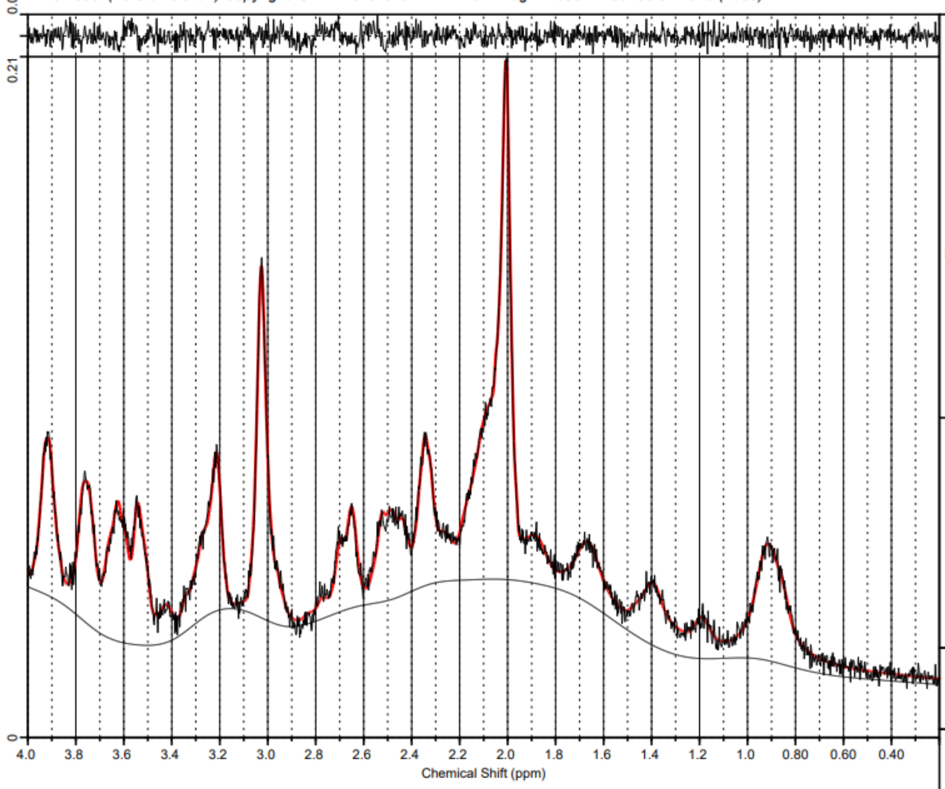

Conc.	%SD	/Cr+PCr	Metabolite
0.173	57%	3.0E-02	Ala
1.254	17%	0.215	Asp
2.225	10%	0.381	Cr
3.610	7%	0.619	PCr
1.173	7%	0.201	GABA
0.971	24%	0.166	Glc
1.618	6%	0.277	Gln
5.808	2%	0.995	Glu
0.605	11%	0.104	GPC
0.322	21%	5.5E-02	PCh
1.145	5%	0.196	GSH
5.076	3%	0.870	Ins
5.70E-02	169%	9.8E-03	Lac
7.908	2%	1.355	NAA
0.682	17%	0.117	NAAG
0.220	15%	3.8E-02	Scyllo
0.804	13%	0.138	Tau
1.642	9%	0.281	-CrCH2
0.927	3%	0.159	GPC+PCh
8.590	2%	1.472	NAA+NAAG
5.835	2%	1.000	Cr+PCr
7.426	2%	1.273	Glu+Gln
0.729	58%	0.125	Lip13a
0.000	999%	0.000	Lip13b
0.532	32%	9.1E-02	Lip09
7.528	9%	1.290	MM09
0.224	53%	3.8E-02	Lip20
7.008	16%	1.201	MM20
1.829	23%	0.314	MM12
4.337	19%	0.743	MM14
5.799	14%	0.994	MM17
0.729	58%	0.125	Lip13a+Lip13b
6.895	16%	1.182	MM14+Lip13a+L
8.060	7%	1.381	MM09+Lip09
7.232	15%	1.240	MM20+Lip20

DIAGNOSTICS

1 info STARTV 24
1 info RFALSI 11
Doing Water-Scaling

MISCELLANEOUS OUTPUT

FWHM = 0.035 ppm S/N = 36
Data shift = 0.010 ppm
Ph: -5 deg -18.6 deg/ppm

MIGRAINE WITHOUT AURA-35yo-Male-Pre-Checkerboard

LCModel (Version 6.3-0B) Copyright: S.W. Provencher. Ref.: Magn. Reson. Med. 30:672-679 (1993). 31-July-2018 13:58

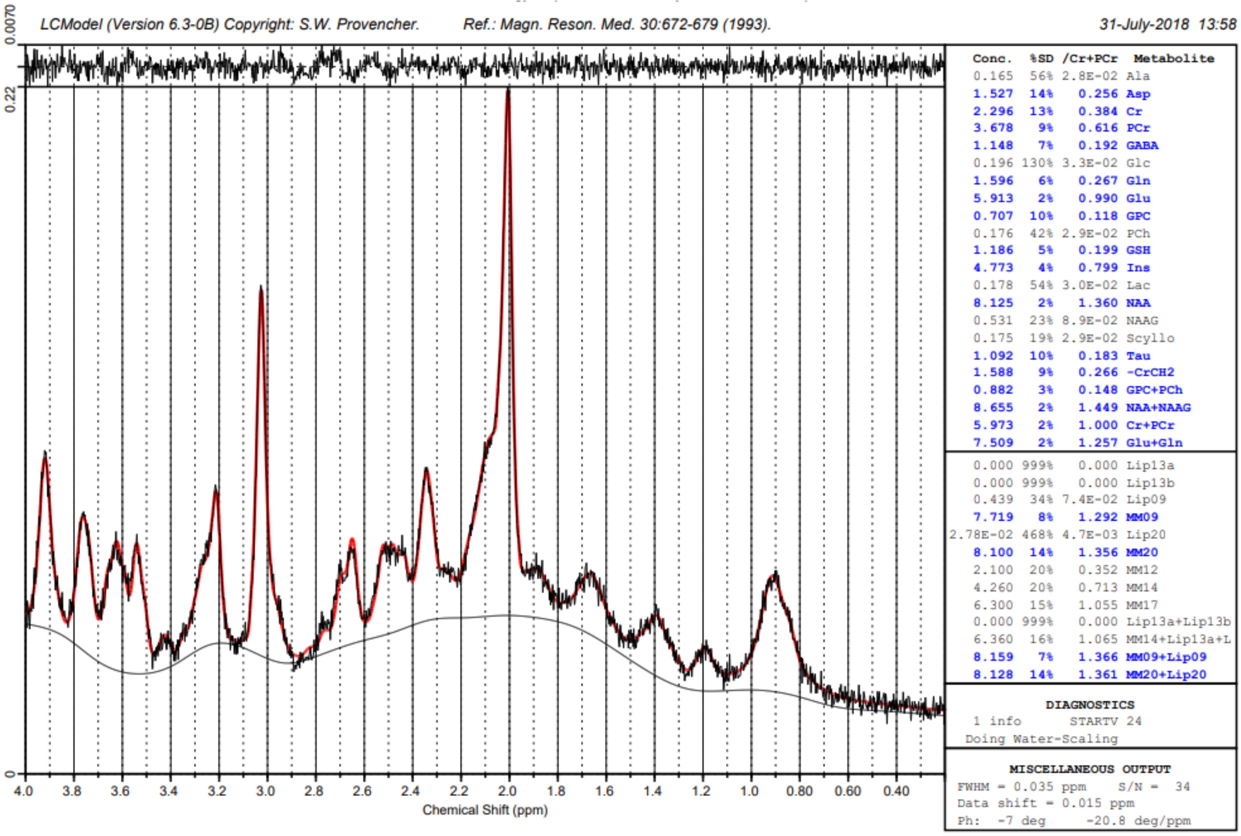

Conc.	%SD	/Cr+PCr	Metabolite
0.165	56%	2.8E-02	Ala
1.527	14%	0.256	Asp
2.296	13%	0.384	Cr
3.678	9%	0.616	PCr
1.148	7%	0.192	GABA
0.196	130%	3.3E-02	Glc
1.596	6%	0.267	Gln
5.913	2%	0.990	Glu
0.707	10%	0.118	GPC
0.176	42%	2.9E-02	PCh
1.186	5%	0.199	GSH
4.773	4%	0.799	Ins
0.178	54%	3.0E-02	Lac
8.125	2%	1.360	NAA
0.531	23%	8.9E-02	NAAG
0.175	19%	2.9E-02	Scyllo
1.092	10%	0.183	Tau
1.588	9%	0.266	-CrCH2
0.882	3%	0.148	GPC+PCh
8.655	2%	1.449	NAA+NAAG
5.973	2%	1.000	Cr+PCr
7.509	2%	1.257	Glu+Gln
0.000	999%	0.000	Lip13a
0.000	999%	0.000	Lip13b
0.439	34%	7.4E-02	Lip09
7.719	8%	1.292	MM09
2.78E-02	468%	4.7E-03	Lip20
8.100	14%	1.356	MM20
2.100	20%	0.352	MM12
4.260	20%	0.713	MM14
6.300	15%	1.055	MM17
0.000	999%	0.000	Lip13a+Lip13b
6.360	16%	1.065	MM14+Lip13a+L
8.159	7%	1.366	MM09+Lip09
8.128	14%	1.361	MM20+Lip20

DIAGNOSTICS

1 info STARTV 24

Doing Water-Scaling

MISCELLANEOUS OUTPUT

FWHM = 0.035 ppm S/N = 34

Data shift = 0.015 ppm

Ph: -7 deg -20.8 deg/ppm

MIGRAINE WITHOUT AURA-36yo-Male-Pre-checkerboard

LCModel (Version 6.3-0B) Copyright: S.W. Provencher. *Ref.: Magn. Reson. Med. 30:672-679 (1993).* *31-July-2018 13:59*

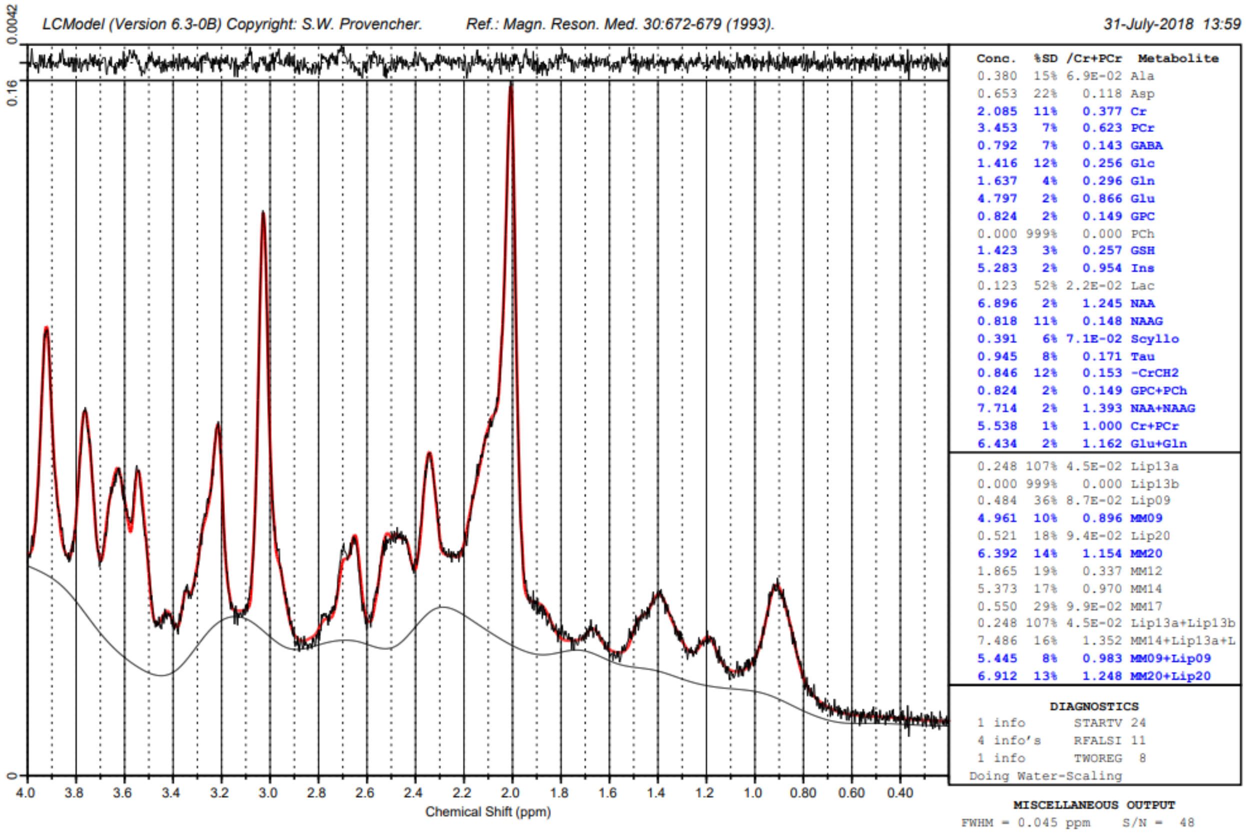

Conc.	%SD	/Cr+PCr	Metabolite
0.380	15%	6.9E-02	Ala
0.653	22%	0.118	Asp
**2.085**	**11%**	**0.377**	**Cr**
**3.453**	**7%**	**0.623**	**PCr**
**0.792**	**7%**	**0.143**	**GABA**
**1.416**	**12%**	**0.256**	**Glc**
**1.637**	**4%**	**0.296**	**Gln**
**4.797**	**2%**	**0.866**	**Glu**
**0.824**	**2%**	**0.149**	**GPC**
0.000	999%	0.000	PCh
**1.423**	**3%**	**0.257**	**GSH**
**5.283**	**2%**	**0.954**	**Ins**
0.123	52%	2.2E-02	Lac
**6.896**	**2%**	**1.245**	**NAA**
**0.818**	**11%**	**0.148**	**NAAG**
**0.391**	**6%**	**7.1E-02**	**Scyllo**
**0.945**	**8%**	**0.171**	**Tau**
**0.846**	**12%**	**0.153**	**-CrCH2**
**0.824**	**2%**	**0.149**	**GPC+PCh**
**7.714**	**2%**	**1.393**	**NAA+NAAG**
**5.538**	**1%**	**1.000**	**Cr+PCr**
**6.434**	**2%**	**1.162**	**Glu+Gln**
0.248	107%	4.5E-02	Lip13a
0.000	999%	0.000	Lip13b
0.484	36%	8.7E-02	Lip09
**4.961**	**10%**	**0.896**	**MM09**
0.521	18%	9.4E-02	Lip20
**6.392**	**14%**	**1.154**	**MM20**
1.865	19%	0.337	MM12
5.373	17%	0.970	MM14
0.550	29%	9.9E-02	MM17
0.248	107%	4.5E-02	Lip13a+Lip13b
7.486	16%	1.352	MM14+Lip13a+L
**5.445**	**8%**	**0.983**	**MM09+Lip09**
**6.912**	**13%**	**1.248**	**MM20+Lip20**

DIAGNOSTICS

1 info	STARTV 24
4 info's	RFALSI 11
1 info	TWOREG 8

Doing Water-Scaling

MISCELLANEOUS OUTPUT

FWHM = 0.045 ppm S/N = 48

Data shift = 0.030 ppm

MIGRAINE WITHOUT AURA-36yo-Male-Post-checkerboard

LCModel (Version 6.3-0B) Copyright: S.W. Provencher. *Ref.: Magn. Reson. Med. 30:672-679 (1993).* *31-July-2018 13:58*

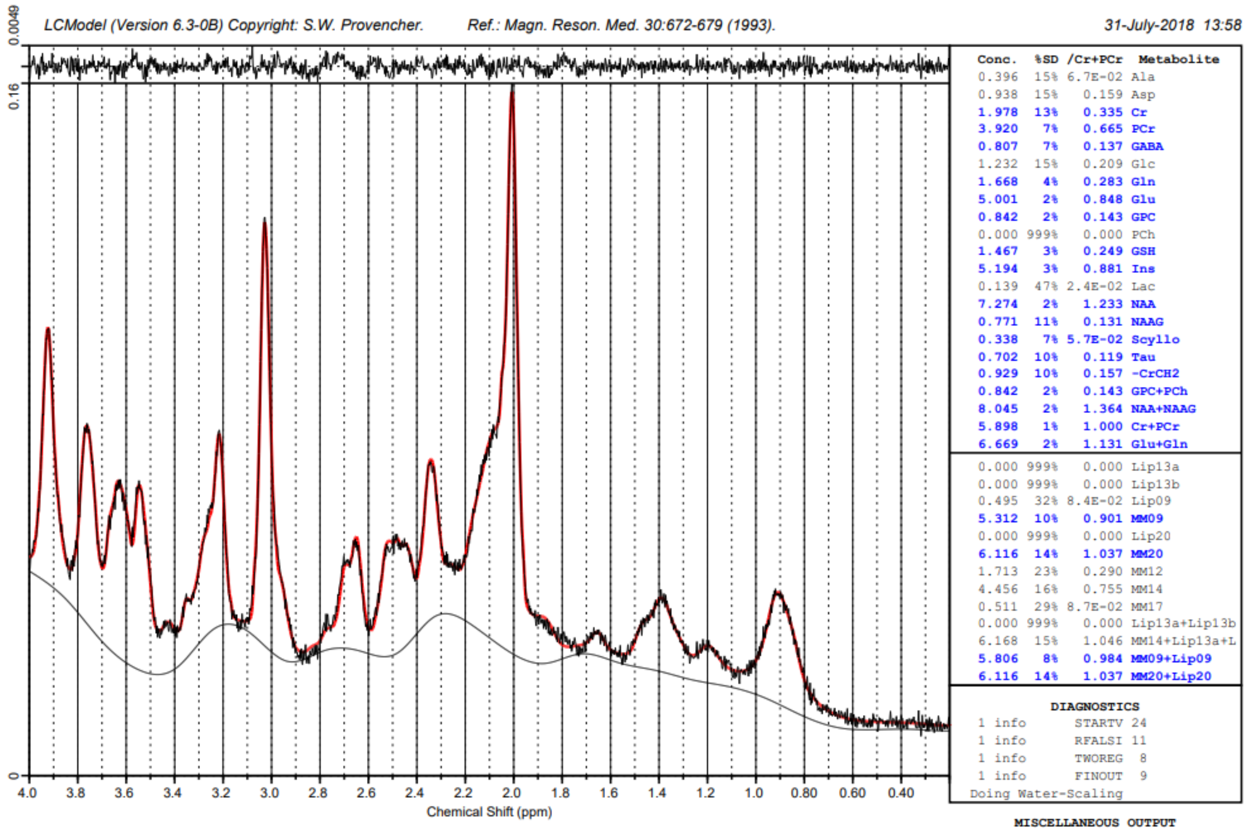

Conc.	%SD	/Cr+PCr	Metabolite
0.396	15%	6.7E-02	Ala
0.938	15%	0.159	Asp
1.978	13%	0.335	Cr
3.920	7%	0.665	PCr
0.807	7%	0.137	GABA
1.232	15%	0.209	Glc
1.668	4%	0.283	Gln
5.001	2%	0.848	Glu
0.842	2%	0.143	GPC
0.000	999%	0.000	PCh
1.467	3%	0.249	GSH
5.194	3%	0.881	Ins
0.139	47%	2.4E-02	Lac
7.274	2%	1.233	NAA
0.771	11%	0.131	NAAG
0.338	7%	5.7E-02	Scyllo
0.702	10%	0.119	Tau
0.929	10%	0.157	-CrCH2
0.842	2%	0.143	GPC+PCh
8.045	2%	1.364	NAA+NAAG
5.898	1%	1.000	Cr+PCr
6.669	2%	1.131	Glu+Gln
0.000	999%	0.000	Lip13a
0.000	999%	0.000	Lip13b
0.495	32%	8.4E-02	Lip09
5.312	10%	0.901	MM09
0.000	999%	0.000	Lip20
6.116	14%	1.037	MM20
1.713	23%	0.290	MM12
4.456	16%	0.755	MM14
0.511	29%	8.7E-02	MM17
0.000	999%	0.000	Lip13a+Lip13b
6.168	15%	1.046	MM14+Lip13a+L
5.806	8%	0.984	MM09+Lip09
6.116	14%	1.037	MM20+Lip20

DIAGNOSTICS

1 info	STARTV	24
1 info	RFALSI	11
1 info	TWOREG	8
1 info	FINOUT	9

Doing Water-Scaling

MISCELLANEOUS OUTPUT

FWHM = 0.045 ppm   S/N = 48
