# Supplementary material for: Relating the cortical visual contrast gain response to spectroscopy-measured excitatory and inhibitory metabolites in people who experience migraine
Source: PLoS One. 2022 Apr 7;17(4):e0266130. doi: 10.1371/journal.pone.0266130 (PMC8989360; doi:10.1371/journal.pone.0266130)
Supplement: S1 Appendix — (DOCX) [file pone.0266130.s003.docx]

# Results for metabolite levels referenced to creatine

## Did the inhibitory and excitatory MRS measures change with checkerboard exposure?

GABA levels did not differ between groups (no main effect of group: F(1,34) = 0.01, p = 0.92) and there was no significant interaction between group and timepoint (pre- and post-checkerboard): F(1,34) = 0.32, p = 0.58). There was also no main effect of timepoint on GABA levels (F(1,34) = 1.25, p = 0.27). Similarly, Glx levels did not differ between groups (no main effect of group: F(1,34) = 0.23, p = 0.64) and there was no significant interaction between group and timepoint: F(1,34) = 0.55, p = 0.46). There was also no main effect of timepoint on Glx levels: F(1,34) = 0.89, p = 0.35).

Glutamine and glutamate levels were not different between groups (no main effect of group: glutamine, F(1,34) = 0.63, p = 0.43; glutamate F(1,34) = 0.10, p = 0.75) and the interaction between group and timepoint was also not significant (glutamine: F(1,34) = 0.31, p = 0.59; glutamate: F(1,34) = 0.77, p = 0.39). These two metabolites were also not significantly changed due to exposure to the flickering checkerboard in both groups (no main effect of timepoint: glutamine, F(1,34) = 1.35, p = 0.25; glutamate, F(1,34) = 0.34, p = 0.57).

These results are consistent with the outcomes derived from metabolite levels referenced to unsuppressed water signal.

## Did metabolites related to neural energy demand change with exposure to the flickering checkerboard?

Glucose levels did not differ between groups (no main effect of group: F(1,34) = 3.10, p = 0.09), and there was no significant interaction between group and timepoint: F(1,34) = 0.47, p = 0.50). However, comparing pre- to post-checkerboard, glucose levels were significantly reduced (main effect of timepoint: F(1,34) = 12.10, p = 0.002).

The levels of the remaining key metabolites relating to neural energy demand, aspartate, lactate and glutamate, did not differ between groups (no main effect of group: Asp: F(1,34) = 0.11, p = 0.75; Lac: F(1,34) = 0.005, p = 0.95; Glu: F(1,34) = 0.10, p = 0.75) nor timepoint (Asp: Lac: F(1,34) = 3.43, p = 0.07; Lac: F(1,34) = 0.008, p = 0.93; Glu: F(1,34) = 1.35, p = 0.25). There was also no significant interaction between group and timepoint for any of these three metabolites (Asp: F(1,34) = 0.67, p = 0.42; Lac: F(1,34) = 0.006, p = 0.94, Glu: F(1,34) = 0.77, p = 0.39).

These results are consistent with the outcomes derived from metabolite levels referenced to the unsuppressed water signal.

## Did the individual change in pre-post checkerboard levels of MRS-measured metabolites correlate with EEG contrast gain estimates?

Upon correcting for multiple comparisons (Bonferroni correction; p<0.008), no significant correlation was present between the change in EEG response amplitude (R97-R0) with GABA, glutamine, glutamate, glucose, aspartate nor lactate levels (see Table below detailed statistics).

These are consistent with the outcomes derived from metabolite levels referenced to unsuppressed water signal.

**Table 2. Pearson’s correlation outcomes for the correlations between the change in MRS-measured metabolites with the change in EEG response amplitude (R97-R0).**

|  | GABA | Glutamine | Glutamate | Glucose | Aspartate | Lactate |
| --- | --- | --- | --- | --- | --- | --- |
| Pearson’s r | 0.10 | 0.00 | -0.13 | 0.07 | 0.18 | 0.51 |
| *p*-value | 0.65 | 0.99 | 0.57 | 0.77 | 0.94 | 0.02 |
